# Current and Future Climate Extremes Over Latin America and Caribbean: Assessing Earth System Models from High Resolution Model Intercomparison Project (HighResMIP)

**DOI:** 10.1007/s41748-022-00337-7

**Published:** 2022-12-19

**Authors:** Alvaro Avila-Diaz, Roger Rodrigues Torres, Cristian Felipe Zuluaga, Wilmar L. Cerón, Lais Oliveira, Victor Benezoli, Irma Ayes Rivera, Jose Antonio Marengo, Aaron B. Wilson, Felipe Medeiros

**Affiliations:** 1 Universidad de Ciencias Aplicadas y Ambientales - UDCA, Bogotá, Colombia; 2 Natural Resources Institute, Universidade Federal de Itajubá, Itajubá, MG Brazil; 3 Department of Agricultural Science, UNISARC - Corporación Universitaria Santa Rosa de Cabal, Santa Rosa de Cabal, Risaralda Colombia; 4 Departamento de Geografía, Facultad de Humanidades, Universidad del Valle, Cali, 760032 Colombia; 5 Department of Agricultural Engineering, Universidade Federal de Viçosa, Viçosa, MG Brazil; 6 Programa de Pós-Gradução em Clima e Ambiente, Instituto Nacional de Pesquisa da Amazônia/Universidade do Estado do Amazonas, Manaus, Brazil; 7 Alliance Bioversity, International Center for Tropical Agriculture (CIAT), Tegucigalpa, Honduras; 8 National Center for Monitoring and Early Warning of Natural Disasters - CEMADEN, São Jose dos Campos, Brazil; 9 Byrd Polar and Climate Research Center, The Ohio State University, Columbus, OH USA; 10 Department of Extension, College of Food, Agricultural, and Environmental Sciences, The Ohio State University, Columbus, OH USA; 11 Graduate Program in Climate Sciences, Federal University of Rio Grande do Norte, Natal, RN Brazil

**Keywords:** CHIRPS, CMIP6, ERA5, GMFD, IPCC

## Abstract

Extreme temperature and precipitation events are the primary triggers of hazards, such as heat waves, droughts, floods, and landslides, with localized impacts. In this sense, the finer grids of Earth System models (ESMs) could play an essential role in better estimating extreme climate events. The performance of High Resolution Model Intercomparison Project (HighResMIP) models is evaluated using the Expert Team on Climate Change Detection and Indices (ETCCDI) over the 1981–2014 period and future changes (2021–2050) under Shared Socioeconomic Pathway SSP5–8.5, over ten regions in Latin America and the Caribbean. The impact of increasing the horizontal resolution in estimating extreme climate variability on a regional scale is first compared against reference gridded datasets, including reanalysis, satellite, and merging products. We used three different groups based on the resolution of the model’s grid (*sg*): (i) low (0.8° ≤ *sg* ≤ 1.87°), (ii) intermediate (0.5° ≤ *sg* ≤ 0.7°), and (iii) high (0.23° ≥ *sg* ≤ 0.35°). Our analysis indicates that there was no clear evidence to support the posit that increasing horizontal resolution improves model performance. The ECMWF-IFS family of models appears to be a plausible choice to represent climate extremes, followed by the ensemble mean of HighResMIP in their intermediate resolution. For future climate, the projections indicate a consensus of temperature and precipitation climate extremes increase across most of the ten regions. Despite the uncertainties presented in this study, climate models have been and will continue to be an important tool for assessing risk in the face of extreme events.

**Supplementary Information:**

The online version contains supplementary material available at 10.1007/s41748-022-00337-7.

## Introduction

Recent reports bolster the connections between climate change and high-impact extreme events such as heavy rainfall, droughts, heat waves, cold waves, and tropical cyclones, which have triggered numerous floods, landslides, wildfires, and avalanches across Latin America and the Caribbean (IPCC 2022; WMO 2021a, b). These regions are considered highly vulnerable to current and future climate extremes due to many factors, including low socio-economic development (Collins et al. 2016; Reyer et al. 2017; Seneviratne et al. 2021) and economic dependence on agricultural commodities (Marengo et al. 2014). According to Nagy et al. (2018), between 2000 and 2015, approximately 74 million people in South America were affected by floods, storms, landslides, and extreme temperatures. For example, the extreme drought in the Brazilian Pantanal between 2019 and 2020 (Libonati et al. 2022; Marengo et al. 2021b) and the drought in the Parana Plata basin (Naumann et al. 2021) not only affected human activities over southern South America but may have exacerbated fire activity that affected the natural biodiversity in Bolivia during 2019 and Pantanal in 2020 (Baxter et al. 2020; Marengo et al. 2021a). Likewise, the flood of 2021 in Manaus, Brazil, has been reported as one of the largest Amazon River flood events of the twenty-first century (Espinoza et al. 2022).

The story is similar to Central America and the Caribbean. Between 2015 and 2019, a prolonged rainfall deficit over most of Central America resulted in severe droughts and crop losses (Depsky and Pons 2021; Pascale et al. 2021; WMO 2021a). The number of tropical cyclones globally was above average in 2020, with 96 events across the 2020 Northern Hemisphere and 2019–20 Southern Hemisphere seasons. Two major hurricanes made landfall in quick succession in Central America (Category 4 Eta and Category 5 Iota), causing severe flooding in the region and the first Category 5 system to strike the Nicaraguan coast. The Honduran Government estimated that 53,000 hectares of crops were devastated by Hurricane Eta, and more than 2.8 million people were affected. Combined with the COVID-19 pandemic and pre-existing humanitarian crises, these extreme compound events left incredible loss and suffering (Shultz et al. 2021), bringing our response to these events into focus.

According to the WMO in its recent report on the State of the Climate in Latin America and the Caribbean (WMO 2022), in the period 1981–2010, the trends indicate an increase in the intensity and frequency of hot extremes and decrease in the intensity and frequency of cold extremes, as well as a significant intensification of total and heavy precipitation in south-eastern South America. As for droughts and dry spells, the report identified mixed trends in different subregions of the Caribbean and Central America, while in Mexico, central Chile, and the Paraná–La Plata Basin, there is some evidence of increased frequency and severity of meteorological droughts. On the other hand, the Seneviratne et al. (2021) project, throughout the twenty-first century, increases in the frequency, duration, and magnitude of warm daily temperature extremes and decreases in cold extremes, as well as an increase in heavy precipitation or the proportion of total rainfall from heavy rainfalls, mainly in tropical regions.

Understanding the dynamics and trends in extreme climate events provides vital information to help policymakers establish actions necessary to combat climate change and its impacts. Providing reliable information regarding historical and future projections of such events represents an enormous challenge for climate researchers (McPhillips et al. 2018; Medeiros and Oliveira 2022; Mistry 2019; Mysiak et al. 2018; Santos et al. 2017). Previous studies have investigated the historical evolution of climate extremes in different parts of the world, including Latin America and the Caribbean, using the set of indices established by the Expert Team on Climate Change Detection and Indices (ETCCDI) (Aguilar et al. 2005; Avila-Diaz et al. 2020b; Cornes and Jones 2013; Donat et al. 2013, 2016; Dunn et al. 2020; Gouveia et al. 2022; Kitoh and Endo 2016; Nakaegawa et al. 2014; Skansi et al. 2013; Zilli et al. 2017). Those studies have indicated two components linked to uncertainties of gridded or observational datasets (e.g., reanalyses and satellite products) and climate simulations (e.g., Earth system models—ESMs) concerning estimates of climate extremes events at local and regional scales: (i) these events have large temporal and spatial variability (Akinsanola et al. 2020; Avila-Diaz et al. 2020b; Campozano et al. 2016; Na et al. 2020); (ii) the assessments of climate extremes in the Northern Hemisphere are more abundant and reliable when compared to the Southern Hemisphere (Donat et al. 2016; Lehmann et al. 2015; Sillmann et al. 2013), due to the fact that in the latter there is a greater lack of climatic data for an analysis of long periods and low spatial distribution of meteorological stations (Condom et al. 2020; Liebmann and Allured 2006; Pabón-Caicedo et al. 2020; Solman 2013). However, despite the deficiencies mentioned above, information from reanalyses, satellites, and the combinations of both, are useful and reliable datasets to evaluate and validate the ESMs (Beck et al. 2019; Contractor et al. 2020; Sun et al. 2018; Yin et al. 2013).

Natural (e.g., El Niño-Southern Oscillation) and anthropogenic (e.g., land–use/land—cover change, fossil fuel burning) forcings can influence the frequency and intensity of climate extreme events, leading to an intensification of hazards such as floods, droughts, fires, cold/heat waves, and landslides (AghaKouchak et al. 2020; Changnon et al. 2000; Chen and Sun, 2021). To improve the representation of the climate extremes patterns and variability, climate scientists have been applied new physical parameterizations to the ESMs (e.g., to improve biosphere–atmosphere interaction processes), increased the horizontal spatial resolution, and used large Multi-Model Ensembles (MME) with a large number of simulations (Bador et al. 2020; Lehner et al. 2020). In this sense, recent studies have shown the effects of the improvements on the parameterizations in the last two generations of ESMs from the CMIP (CMIP5 and CMIP6) (Brown et al. 2020; Fan et al. 2020; Lun et al. 2021; Ortega et al. 2021; Thorarinsdottir et al. 2019; Wehner 2020). However, all the studies agree that the improvements in the simulation of temperature and precipitation climate extremes are small and statistically not significant.

Moreover, studies analyzing the sources of ESMs’ uncertainties from CMIP experiments are focused on extensive areas, like continental regions (Almazroui et al. 2021c; Kim et al. 2020; Na et al. 2020; Sillmann et al. 2013). In a recent study, Akinsanola et al. (2020) found that MME from CMIP6 performs better than most individual models in capturing precipitation extremes at a seasonal scale over the United States. On the other hand, most regional studies indicated that to assess the ability of CMIP models to capture the variability of climate extremes, it is necessary to evaluate the performance of individual models to identify the shortcomings (Akinsanola et al. 2020; Avila-Diaz et al. 2020b; Medeiros and Oliveira 2022; Rivera and Arnould 2020).

Finally, to increase the spatial resolution, Regional Climate Models (RCMs) have traditionally been used through dynamical downscaling of ESM outputs to obtain finer climate information for a particular region (Ban et al. 2021; Vichot-Llano et al. 2021). However, Denis et al. (2002) show that although RCMs provide a more detailed representation of the complex topography and the continent–ocean contrast, they introduce new sources of uncertainty (Giorgi 2005; Giorgi and Francisco 2001) like closure problems in lateral boundary conditions (Ambrizzi et al. 2019; de Medeiros et al. 2020). To counteract this drawback, high-resolution ESMs are being developed, which have the potential to provide relevant regional and global climate information and include more climate processes than RCMs (Demory et al. 2020). An example is the new High Resolution Model Intercomparison Project—HighResMIP (Haarsma et al. 2016), which provides an evaluation framework for ESM simulations in horizontal grid spacings ranging from 0.18º to 2.5º. In this way, it is possible to understand the role of increasing horizontal resolution in climate simulations (mean and extreme values, variability, etc.). This increase in spatial resolution has shown considerable improvements in the simulations of the magnitude and frequency of meteorological systems of different scales, such as tropical cyclones (Roberts et al. 2020; Vannière et al. 2020) and atmospheric blocking events (Schiemann et al. 2020).

The main objective of this study is to assess the performance of a sub-set of HighResMIP models, which are members of the CMIP6, in simulating daily temperature and precipitation climate extremes events (as represented by the indices recommended by the ETCCDI) over Latin America and the Caribbean regions during 1981–2014. Additionally, we evaluate the impact of the increase in the horizontal spatial resolution in the HighResMIP models in estimating extreme climate variability on a local/regional scale. Finally, we analyze climate projections for the 2021–2050 period under the new Shared Socioeconomic Pathways (SSP) scenario SSP5-8.5.

## Data and Methodology

### Climate Extremes Indices

Table 1 shows the temperature (8) and precipitation (8) extremes indices chosen as the most relevant for the studied region from the 27 indices proposed by the ETCCDI (http://etccdi.pacificclimate.org/). The selected ETCCDI indices have been widely used for monitoring changes in daily extremes of temperature and precipitation in Latin America and the Caribbean regions (Aguilar et al. 2005; Almazroui et al. 2021c; Avila-Diaz et al. 2020b; Collins et al. 2016; Heidinger et al. 2018; Skansi et al. 2013; Valverde and Marengo 2014). These indices are calculated from daily maximum (TX) and minimum (TN) temperature and precipitation (PR) data. The capital “X” and “N” stand for the daily maximum and minimum temperature, respectively. Indices can be classified into four groups: (1) absolute indices such as hottest day (TXx) and coldest night (TNn) or daily and 5-day maximum PR (RX1day and RX5day, respectively); (2) threshold indices that represent the number of days exceeding a fixed threshold, as the number of days with PR greater than 20 mm (R20mm); (3) percentile-based threshold indices, that indicate the number of days that is surpassing rates below 10th percentile (cold nights—TN10p and cold days—TX10p) or above 90th percentile (warm nights—TN90p and warm days—TX90p); and (4) duration indices, that display the warm spell duration (WSDI, based on percentile thresholds), dry spell (consecutive dry days—CDD) and wet spell (consecutive wet days -CWD), based on an absolute threshold. In the case of the absolute indices, the lower case “x” and “n” means the annual maximum and minimum value, respectively. We used the same reference period (1981–2014) for all ESMs to calculate the percentile-based threshold indices. Moreover, we refer to the interested reader to see Zhang et al. (2011) for further details about ETCCDI indices.

**Table 1:** List of the temperature and precipitation indices applied in this study

Index	Indicator name	Indicator definitions	Units
1. TXx	Hottest day	Let ${Tx}_{kj}$ be the daily maximum temperatures for the interval $k$, period $j$. The maximum daily maximum temperature in the period, then: ${TXx}_{kj}=\mathrm{max}({Tx}_{kj})$	ºC
2. TNn	Coldest nights	Let ${Tn}_{kj}$ be the daily minimum temperatures in for the interval $k$, period $j$. The minimum daily minimum temperature in the period, then:${TNn}_{kj}=\mathrm{min}({Tn}_{kj})$	ºC
3. DTR	Diurnal temperature range	Let ${Tx}_{ij}$ and ${Tn}_{ij}$ be the daily maximum and minimum temperature, respectively, on day $i$ in period $j$. If $I$ represents the number of days in $j$, then:${DTR}_{j}={\sum }_{{\varvec{i}}=1}^{{\varvec{I}}}\left({Tx}_{ij} -{Tn}_{ij}\right)/I$	ºC
4. TN10p	Cold nights	Let ${Tn}_{ij}$ be the daily minimum temperature on day $i$ in period $j$ and let ${Tn}_{in}10$ be the calendar day 10th percentile centerd on a 5-day window. The percentage of time is determined where:${Tn}_{ij} <{Tn}_{in}10$	% of days
5. TN90p	C	Let ${Tn}_{ij}$ be the daily minimum temperature on day $i$ in period $j$ and let ${Tn}_{in}90$ be the calendar day 90th percentile centered on a 5-day window. The percentage of time is determined where:${Tn}_{ij}>{Tn}_{in}90$	% of days
6. TX10p	Cold days	Let ${Tx}_{ij}$ be the daily maximum temperature on day $i$ in period $j$ and let ${Tx}_{in}10$ be the calendar day 10th percentile centered on a 5-day window. The percentage of time is determined where:${Tx}_{ij}<{Tx}_{in}10$	% of days
7. TX90p	Warm days	Let ${Tx}_{ij}$ be the daily maximum temperature on day $i$ in period $j$ and let ${Tx}_{in}90$ be the calendar day 90^th^ percentile centered on a 5-day window. The percentage of time is determined where:${ Tx}_{ij} >{Tx}_{in}90$	% of days
8. WSDI	Warm spell duration indicator	Let ${Tx}_{ij}$ be the daily maximum temperature on day $i$ in period $j$ and let ${Tx}_{in}90$ be the calendar day 90th percentile centered on a 5-day window. Then the number of days per period is summed where, in intervals of at least 6 consecutive days:${Tx}_{ij} >{Tx}_{in}90$	days
9. PRCPTOT	Annual total wet-day precipitation	Let ${PR}_{ij}$ be the daily precipitation amount on day $i$ in period $j$. If $I$ represents the number of days in $j$, then $PRCPTOTj={\sum }_{i=1}^{I}{PR}_{ij}$	mm
10. RX1day	Max 1-day precipitation amount	Let ${PR}_{ij}$ be the daily precipitation amount on day $i$ in period $j$. Then maximum 1-day values for period $j$ are:${Rx1day}_{j}=\mathrm{max}({PR}_{ij})$	mm
11. RX5day	Max 5-day precipitation amount	Let ${PR}_{ij}$ be the precipitation amount for the 5-day interval ending $k$, period $j$. Then maximum 5-day values for period $j$ are: ${Rx5day}_{j}=\mathrm{max}({PR}_{ij})$	mm
12. R95p	Very wet days	Let ${PR}_{wj}$ be the daily precipitation amount on a wet day $w(PR\ge 1.0\mathrm{mm})$ in period $j$ and let ${PR}_{wj}95$ be the 95 percentile of precipitation on wet days in the 1981–2014 period. If $W$ represents the number of wet days in the period, then:$R95pj={\sum }_{\text{w=1}}^{W}{PR}_{wj}\text{ where }{PR}_{wj}>{PR}_{nj}95$	mm
13. SDII	Simple daily intensity index	Let ${PR}_{wj}$ be the daily precipitation amount on wet days,$w(RR\ge 1\mathrm{mm})$ in period $j$. If $W$ represents number of wet days in $j$, then:${SDII}_{j}=\frac{{\sum }_{w=1}^{W}{PR}_{wj} }{W}$	mm/day
14. R20mm	Number of very heavy precipitation days	Let ${PR}_{ij}$ be the daily precipitation amount on day $i$ in period $j$. Count the number of days where:${PR}_{ij}\ge 20\mathrm{mm}$	days
15. CWD	Consecutive wet days	Let ${PR}_{ij}$ be the daily precipitation amount on day $i$ in period $j$. Count the largest number of consecutive days where:${PR}_{ij} j\ge 1\mathrm{mm}$	days
16. CDD	Consecutive dry days	Let ${PR}_{ij}$ be the daily precipitation amount on day $i$ in period $j$. Count the largest number of consecutive days where:${PR}_{ij} <1\mathrm{mm}$	days

More details can be found in Zhang et al. (2011). Wet (dry) days are defined when the precipitation ≥ 1 mm (≤ 1 mm)

Some studies have already employed the ETCCDI indices to monitor changes in intensity, frequency, and duration of temperature and precipitation climate extremes over the study area using meteorological stations (Aguilar et al. 2005; Ávila et al. 2019; Ceron et al. 2020; Croitoru et al. 2016; Domínguez-Castro et al. 2020; Marengo et al. 2021a). Other works assess the skills of the different gridded datasets (e.g., ESMs, reanalyses, satellites products) to reproduce the spatial–temporal variability (Avila-Diaz et al. 2021; de Lima and Alcântara 2019; Kim et al. 2020; Ongoma et al. 2018). In this sense, we evaluated the performance of CMIP6 models in simulating the ETCCDI indices using annual summary values, similar to other studies (Aerenson et al. 2018; Gouveia et al. 2022; Thibeault and Seth 2014) that indicate that most of the impactful climate extremes can be described by annual indices. Additional details about the performance of CMIP6 models over North, Central, and South America on the annual cycle of total precipitation and mean temperature are discussed by Almazroui et al. (2021a, b).

### Climate Reference Datasets

The reference climate datasets used in this study are the European Centre for Medium-Range Weather Forecasts (ECMWF) Reanalysis v5—ERA5 (Hersbach et al. 2020), the Global Meteorological Forcing Dataset for Land Surface Modeling—GMFD (Sheffield et al. 2006), and the Climate Hazards Group InfraRed Precipitation with Stations—CHIRPS v.2.0 (Funk et al. 2015). We focus on these gridded datasets because they are available over our study region with a high horizontal resolution covering the reference period defined in this study (1981–2010) and are widely used in the literature. It must be stressed that the level of model performance in simulating climate extremes varies according to the reference datasets (Kim et al. 2020; Sillmann et al. 2013; Srivastava et al. 2020; Ortega et al. 2021). Moreover, some studies showed that using more than one reference dataset reduces the uncertainty of the observational climate gridded information (Avila-Diaz et al. 2021; Bador et al. 2020).

ERA5 data are available from 1950 to the present with a temporal resolution of 1 h and horizontal spatial resolution of 0.25° × 0.25° lat/lon (Hersbach et al. 2020). According to CDS (2020; https://confluence.ecmwf.int/display/CKB/ERA5), in ERA5, the minimum and maximum temperatures are forecast parameters, that is, they are available from the forecasts only, and they have a cold bias in the lower regions of the troposphere over most parts of the globe. Precipitation is a variable available from a combination of data analysis and forecasting, and due to the increased spatial resolution, it presents improved results in ERA5 (Jiao et al. 2021; Tarek et al. 2020). However, important uncertainties remain in tropical regions due to the lack of data available to analyze (Hersbach et al. 2020). In some studies, ERA5 has demonstrated reliable estimates of climate features in some regions of Central and South America (Avila-Diaz et al. 2020b; Balmaceda‐Huarte et al. 2021; Cerón et al. 2021; Zuluaga et al. 2021).

The CHIRPS (Funk et al. 2015) dataset provides daily precipitation outputs at a high horizontal resolution (0.05° × 0.05° lat/lon) with near-global coverage (50° S to 50° N, and 180° W to 180° E) from 1981 to near the present. CHIRPS precipitation combines interpolated station data with the Tropical Rainfall Measuring Mission Multi-Satellite Precipitation Analysis version 7 (TMPA 3B42 v7) to calibrate global Cold Cloud Duration (CCD) rainfall estimates (Funk et al. 2015). Previous studies show that CHIRPS can simulate spatiotemporal precipitation variability for particular regions of South America (Cerón et al. 2020, 2021; Espinoza et al. 2019; Nogueira et al. 2018; Rivera et al. 2018).

The GMFD data have a horizontal resolution of 0.25° × 0.25° lat/lon and provide precipitation and maximum and minimum temperature daily outputs available during the 1948–2016 period (Sheffield et al. 2006). It is constructed from the coupling of the NCEP-NCAR global datasets and reanalyses, daily precipitation from the Global Precipitation Climatology Project (GPCP), and monthly climate variables from the Climatic Research Unit (CRU), 3-hourly temporal resolution precipitation from TRMM, and the NASA Langley monthly surface radiation budget. GMFD has demonstrated reliable patterns for daily temperature extremes; however, it is inadequate to estimate daily precipitation extremes over South America (Avila-Diaz et al. 2020a; b). Therefore, we only used the daily outputs of maximum and minimum temperature.

### Earth System Models Simulations and Projections

According to Haarsma et al. (2016), to assess the historical period (1950–2014), the HighResMIP the following uses two experiments: (1) *hist-1950*, based on historical coupled ocean atmosphere simulations of the near past at high and standard resolution, and (2) *highresSST-present*, based on historical atmosphere-only simulations of the near past, driven by sea surface temperature and sea ice concentration data obtained from HadISST2.2 at 1/4 degree daily resolution.

To capture solely the impact of atmospheric resolution on temperature and precipitation extremes, this analysis focuses on *highresSST-present* experiment, which evaluates a set of HighResMIP simulations forced by observed daily sea surface temperature (SST) data from the HadISST.2.2.0.0 dataset, available for the 1950–2014 period at https://esgf-node.llnl.gov/search/cmip6/. The performance of the HighResMIP simulations is evaluated over the 1981–2014 period. We used the daily climate outputs (e.g., PR, TX, and TN) of 21 ESMs from 10 different meteorological modeling institutions, executed in (at least) two different spatial resolutions (Table 2). This study considered the first ensemble member of all ESMs (i.e., r1i1p1f1), except for the CNRM-CM6-1, that only is available for r1i1p1f2. For more details about HighResMIP experimental design, see Haarsma et al. (2016).

**Table 2:** List of selected CMIP6 Earth System Models in this study

Institution, Country	Model	Horizontal resolution	Group	PR	TX	TN
CMCC, Italy	CMCC-CM2-HR4	0.94° × 1.25°	1	**	**–**	**–**
	CMCC-CM2-VHR4	0.23° × 0.31°	3	**	**–**	**–**
CNRM-CERFACS, France	CNRM-CM6-1	1.40° × 1.40°	1	**	**	**
	CNRM-CM6-1-HR	0.50° × 0.50°	2	**	**	**
EC-Earth-Consortium, Europe	EC-Earth3P	0.70° × 0.70°	2	**	**	**
	EC-Earth3P-HR	0.35° × 0.35°	3	**	**	**
ECMWF, Europe	ECMWF-IFS-LR	1.00° × 1.00°	1	*	*	*
	ECMWF-IFS-HR	0.50° × 0.50°	2	*	*	*
IAP and CAS, China	FGOALS-f3-L	1.25° × 1.00°	1	**	**	**
	FGOALS-f3-H	0.25° × 0.25°	3	**	*	*
NERC and MOHC, United Kingdom	HadGEM3-GC31-LM	1.25° × 1.87°	1	**	**	**
	HadGEM3-GC31-MM	0.55° × 0.83°	2	**	**	**
	HadGEM3-GC31-HM	0.23° × 0.35°	3	**	**	**
IPSL, France	IPSL-CM6A-LR	1.25° × 2.50°	1	*	*	*
	IPSL-CM6A-ATM-HR	0.70° × 0.50°	2	*	–	*
MPI, Germany	MPI-ESM1-2-HR	0.93° × 0.93°	1	*	*	*
	MPI-ESM1-2-XR	0.46° × 0.46°	2	*	*	*
MRI, Japan	MRI-AGCM3-2-H	0.70° × 0.56°	2	**	**	**
	MRI-AGCM3-2-S	0.18° × 0.18°	3	**	**	**
MIROC, Japan	NICAM16-7S	0.56° × 0.56°	2	**	**	**
	NICAM16-8S	0.28° × 0.28°	3	**	**	**

The number of groups is related to classification based on horizontal spatial resolution (latitude × longitude): G1: 1.00° × 1.00°, G2: 0.50° × 0.50° and G3: 0.25° × 0.25°. All climate projections are available between 2015 and 2050, except for the EC-Earth3P and EC-Earth3P-HR models, which are available until 2049. The symbols “*” and “**” indicate the availability for the historical period and future periods, respectively. The character “–” specify unavailability for historical and/or future periods

For consistency with the *highresSST-present* experiment, the projections of future scenarios were evaluated with the data from the *highresSST-future* experiment. This last contemplates only a future simulation scenario based on a blend of variability from the 0.25° HadISST2-based dataset and the climate change signal from CMIP5 RCP8.5 simulations. (Haarsma et al. 2016; O’Neill et al. 2016). The CMIP5 RCP8.5 scenario represents the high end of the range of plausible future forcing pathways. It is consistent with high energy intensity, high dependence on fossil fuels, continuous growth in the population, heavy greenhouse gas emissions associated with slow technological development, and no implementation of climate policies (Bozkurt et al. 2018; Silveira et al. 2019).

### Model Performance Metrics

The Kling-Gupta efficiency (KGE) methodology (Gupta et al. 2009; Kling et al. 2012) has been widely adopted to compare diverse-based climate gridded against observed datasets (Avila-Diaz et al. 2021; Beck et al. 2019; Chaney et al. 2014; Nashwan and Shahid 2019; Stewart et al. 2022; Wilson et al. 2022; Zuluaga et al. 2022). The KGE is an index used in this research to compare the reference precipitation/temperature dataset with model estimates; the optimum value of KGE is one (1.0). The total performance of simulations is decomposed into three different metrics with the same weight (Eq. 1) as follows: (i) the linear correlation (CORR), which measures the temporal coherence of the precipitation and temperature indices (Eq. 2), where 1.0 is a perfect score, and 0.0 indicates the absence of correlation (Pearson 1895); (ii) the bias ratio (BR), that is used to measure the overestimation (BR > 1.0) or underestimation (BR < 1.0) compared to the observations (Eq. 3) (Ayehu et al. 2018); and (iii) the relative variability (RV), which is a relative measure of the dispersion (Eq. 3) and its optimal value at unity (1.0).

1 $$\mathrm{KGE}=1- \sqrt{{\left(1-\mathrm{CORR}\right)}^{2}+{\left(1-\mathrm{BR}\right)}^{2}+{\left(1-\mathrm{RV}\right)}^{2}}$$

2 $$\mathrm{Correlation }\,\,(\mathrm{CORR}) =\frac{\sum_{\mathrm{i}=1}^{\mathrm{n}}\left({\mathrm{O}}_{\mathrm{i}}-\overline{\mathrm{O} }\right)\left({\mathrm{S}}_{\mathrm{i}}-\overline{\mathrm{S} }\right)}{\sqrt{\sum_{\mathrm{i}=1}^{\mathrm{n}}{\left({\mathrm{O}}_{\mathrm{i}}-\mathrm{O}\right)}^{2}}\sqrt{\sum_{\mathrm{i}=1}^{\mathrm{n}}{\left({\mathrm{S}}_{\mathrm{i}}-\overline{\mathrm{S} }\right)}^{2}}}$$

3 $$\mathrm{Bias \,\,Ratio }\,\,\left(\mathrm{BR}\right)=\frac{\overline{\mathrm{S}} }{\overline{\mathrm{O}} }$$

4 $$\mathrm{Relative\,\, Variability }\,\,(\mathrm{RV})= \frac{{\mathrm{CV}}_{\mathrm{S}}}{{\mathrm{CV}}_{\mathrm{O}}}$$

The parameters ${\mathrm{O}}_{\mathrm{i}}$ and ${\mathrm{S}}_{\mathrm{i}}$ in Eqs. (2) and (3) are the observation and simulation values, and $\overline{\mathrm{O} }$ and $\overline{\mathrm{S} }$ are their means, respectively. In Eq. (4), ${\mathrm{CV}}_{\mathrm{O}}$ and ${\mathrm{CV}}_{\mathrm{S}}$ are the coefficient of variation of observed and simulated values, respectively.

We used a comprehensive ranking procedure to assess performance across models and resolutions (Wilson et al. 2022; Yang et al. 2020; You et al. 2018). Regional mean KGE values based on comparisons of extreme temperature and precipitation with the reference datasets (e.g., ERA5, GMFD, and CHIRPS) were used to rank the models and MMEs regardless of resolution group from 1 (best) to 21 (worst) (24 for precipitation). These rankings were then summed to produce a cumulative rank score for each model and MME. As KGE assesses both mean and variability, the score and, therefore, the ranking does not distinguish which component leads to a stronger KGE value. For a comparison of each component of the KGE values, see Supplemental Figs. S1 and S2.

### Data Processing

To study the performance and the impact of the increased horizontal resolution of CMIP6 models in capturing climate extremes, we defined the following three different groups based on the size of the grid (*sg*): (1) 0.8° ≤ *sg* ≤ 1.87° (G_1_L), (2) 0.5° ≤ *sg* ≤ 0.7° (G_2_I), and (3) 0.23° ≥ *sg* ≤ 0.35° (G_3_H). The capital “L”, “I”, and “H” means the group is classified as low, intermediate, or high resolution, respectively. We established the different resolution groups to conserve the statistical features of the extreme climate indices in the original resolution of ESMs, following Diaconescu et al. (2015). For intercomparison purposes, after calculating the ETCCDI indices, the ESMs and reference datasets (e.g., ERA5, GMFD, and CHIRPS) in the G_1_L, G_2_I, and G_3_H groups were regridded to a common resolution of 1º × 1º, 0.50° × 0.50° and 0.25° × 0.25°, respectively, using a first-order conservative remapping technique (Jones 1999). All reference climate datasets and CMIP6 models were studied during 1981–2014. Finally, a Multi-Model Ensemble (MME) mean was also calculated for each model group (G_1_L-MME, G_2_I-MME, and G_3_H-MME), and those were compared with the observational data sets.

**Fig. 1:**
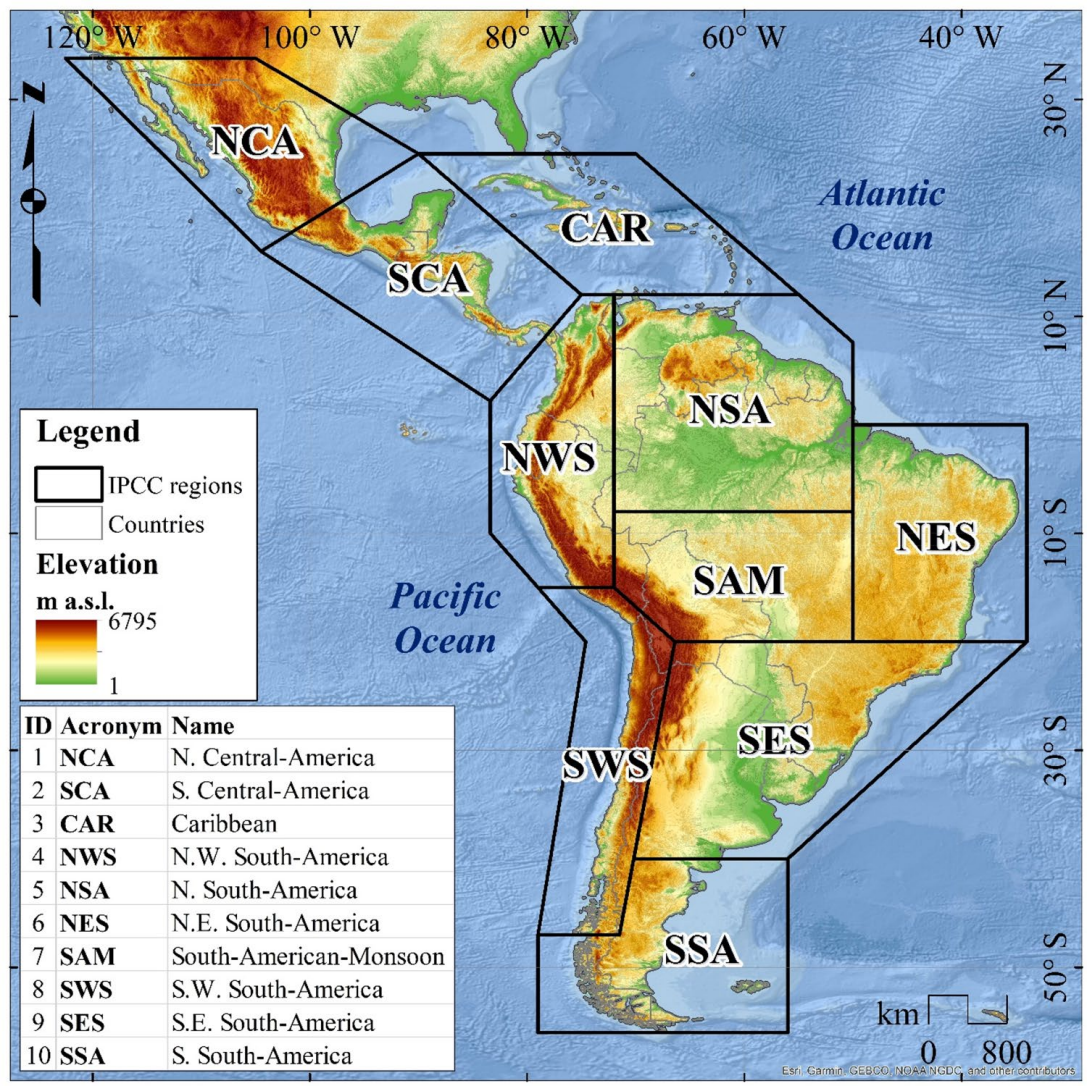
Sub-regional domains over Latin America and the Caribbean, according to Iturbide et al. (2020)

The performance and trend analyses were conducted over ten reference regions of Latin America and the Caribbean, defined by Iturbide et al. (2020) for the Intergovernmental Panel on Climate Change (IPCC) Sixth Assessment Report (AR6). The shapefiles and codes for these regions are available at https://github.com/SantanderMetGroup/ATLAS. The regional acronyms in Fig. 1 refer to (1) North Central America (NCA), (2) South Central America (SCA), (3) Caribbean (CAR), (4) Northwestern South America (NWS), (5) North–South America (NSA), (6) North Eastern South America (NES), (7) South American Monsoon (SAM), (8) South Western South-America (SWS), (9) South Eastern America (SES), and (10) South-South America (SSA).

To assess the future changes in HighResMIP climate projections, we established the relative change in each ETCCDI index using Eq. 5, adapted from Bador et al. (2018) as follows:

5 $$\mathrm{Relative\,\, change\,\, in\,\, a \,\,given\,\, ETCCDI\,\, index }\,\,\left(\mathrm{i}\right)= \frac{{\overline{\mathrm{ETCCDI}} }_{\mathrm{i}-\mathrm{future}}-{\overline{\mathrm{ETCCDI}} }_{\mathrm{i}-\mathrm{his}}}{{\overline{\mathrm{ETCCDI}} }_{\mathrm{i}-\mathrm{his}}},$$

where ${\overline{ETCCDI} }_{i-future}$ and ${\overline{ETCCDI} }_{i-his}$ are the 30-year averages in each ETCCDI index over the future interval (2021–2050) and historical period (1981–2010), respectively.

The agreement of climate change signal in the ETCCDI indices is considered robust if at least 66% of ESMs have the same directional change and more than 50% delivered a significant change using the Student’s *t*-test (*p* value < 0.05) between the historical (reference) and projections (Almazroui et al. 2021b; Avila-Diaz et al. 2020b; Dosio et al. 2019).

## Results

### Earth System Model Evaluation with Observations

#### Performance Evaluation in Temperature Indices

**Fig. 2:**
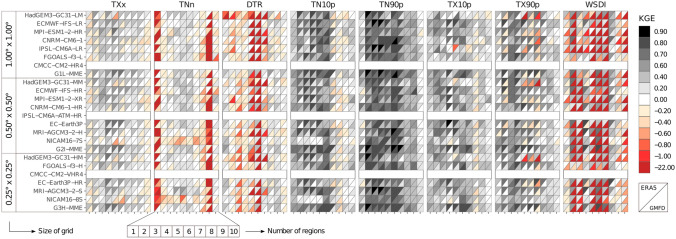
KGE Performance evaluation obtained of ESMs to estimate annual temperature indices compared to ERA5 and GMFD datasets during 1980–2014. The number of regions is the ID according to Fig. 1. The NA (white boxes) means no data value for the index in the CMCC-CM2-HR4, IPSL-CM6-1-ATM-HR, and CMCC-CM2-VHR4

The heat maps summarize the individual performance of the ESMs in simulating the climate extremes indices on the annual scale over Latin America and the Caribbean region from 1981 to 2014 compared to ERA5 and GMFD datasets (Fig. 2). In this sense, most ESMs display better performance for the TXx index and when compared with the ERA5 dataset than GMFD. Furthermore, the families of models, including HadGEM3-GC31, ECMWF-IFS, FGOALS-f3, and EC-Earth3P, show plausible performance over almost all regions, except for SSA regions where most models show KGE values close to 0.0 (Fig. 2, first column 1). Interestingly, increasing the horizontal resolution does not show a continuous improvement for the TXx index.

Figure 2 shows that the best models for capturing TNn patterns for those regions are MPI-ESM1-2-XR, EC-Earth3P, and MRI-AGCM3-2-S with KGE between 0.20 and 0.60. The ESMs presented difficulties in reproducing the minimum temperature (TNn) index for almost all regions, except for CAR, NWS, NSA, and NES regions in which BR and RV are close to 1.0, and a significant correlation above 0.4 is found (Fig. S1). The weakest performance is found in regions NCA, SWS, and SES with extremely negative KGE values (Fig. 2), especially when the ESMs are compared with GMFD, which can exceed − 10.0.

For the diurnal temperature range (DTR), the ESMs display KGE values above 0.4 when compared with ERA5, especially in the NCA, NES, SAM, and SWS regions (Fig. 2; third column). In contrast, in most regions, ESMs showed poor performance (KGE < 0.0) using GMFD as a reference dataset. Similarly, Avila-Diaz et al. (2020b) find this poorer representation, indicating that GMFD displays difficulties in representing observational magnitudes of the DTR index during 1980–2016 over Brazil. On the one hand, these results can be related to the DTR values from each reference dataset. GMFD uses CRU-derived DTR data (Sheffield et al. 2006), which shows low statistical performance (correlation and error) over northern South America and the Caribbean region (Harris et al. 2020). Meanwhile, the DTR values from ERA5 are influenced by the cold bias of TX and TN (Hersbach et al. 2020). On the other hand, Wang and Clow (2020) show that the latest generation of CMIP6 models continues to underestimate DTR climatology, mainly due to difficulties in estimating downwelling longwave radiation, which influences TN daily values.

For percentile indices such as cold nights (TN10p), warm nights (TN90), cold days (TX10p), and warm days (TX90p), most ESMs perform reasonably well compared to the reference datasets (Fig. 2, see fourth to the seventh column). The KGE values are above 0.4, BR and RV are variability close to 1.0, and the significance CORR is above 0.6 in almost all regions (Fig. S1 shows the values of the KGE components). However, less agreement is found in the southern regions of the study area for most ESMs (in the SES region, when used, the ERA5 dataset and the SSA region for GMFD). Therefore, simulations of those indices are overestimated in most regions concerning GMFD, except for TX10p in the NWS region. Furthermore, the bias of ESMs compared to ERA5 display mixed results of over/underestimating in the percentiles indices (Fig. S1).

When comparing the reference data sets, the KGE values between ESMs and ERA5 are better than between ESMs and GMFD in most regions for warm spell duration index (WSDI; Fig. 2 eighth column). Furthermore, according to GMFD, the weaker performance of ESMs is displayed for the NSA, SAM, and SES regions with negative KGE values (Fig. 2). In this sense, Avila-Diaz et al. (2020a) evaluated the performance of 21 statistically downscaled ESMs at high horizontal resolution (0.25° of latitude × longitude) over Brazil. They indicate that the worst performance across the ETCCDI indices is for WSDI. This shortcoming may be related to the fact that 21 ESMs use the GMFD data to downscale the CMIP5 models.

**Fig. 3:**
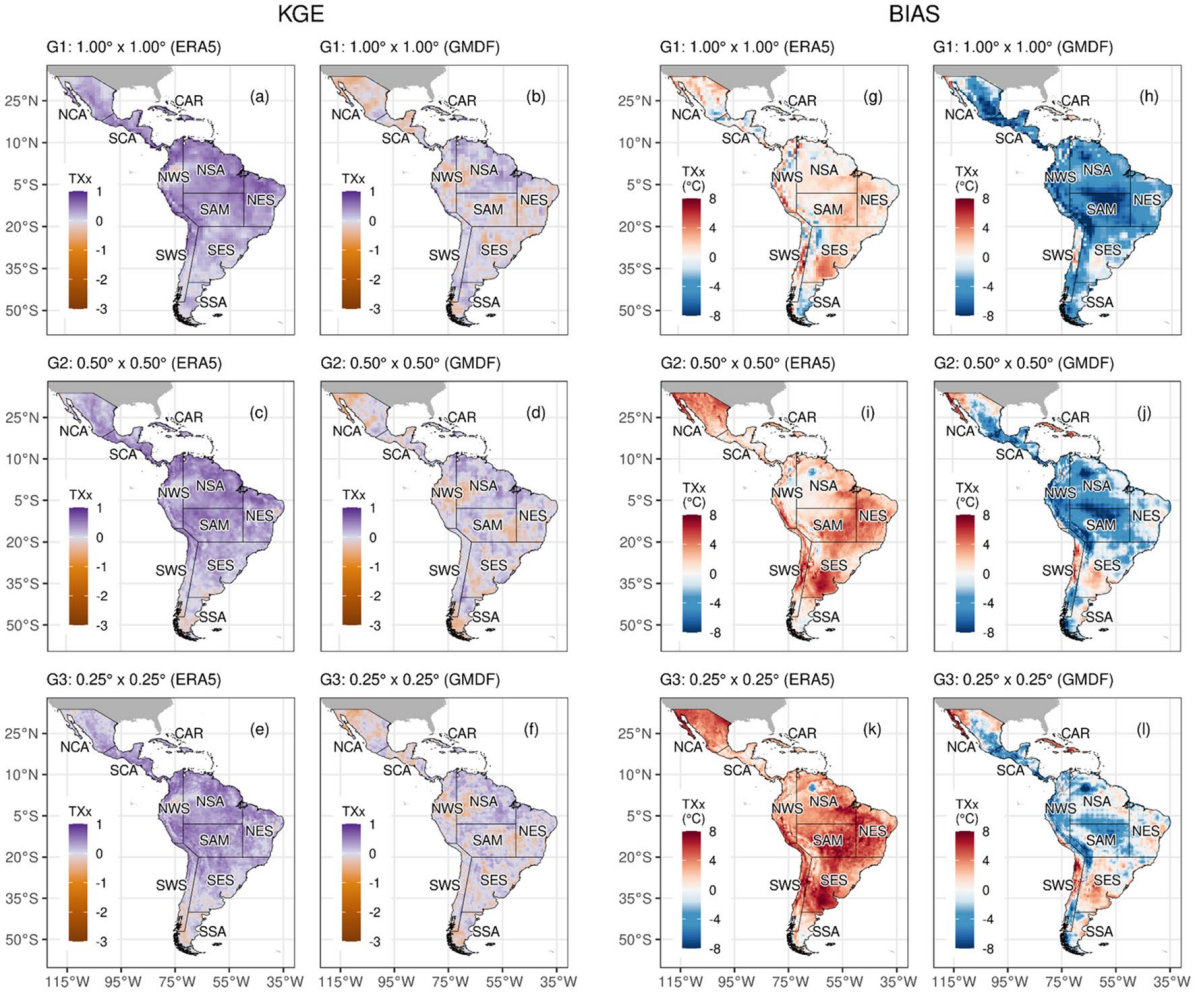
The KGE (**a**–**f**) and climatology bias (**g**–**l**) of multi-model ensemble (MME) for the TXx during 1981–2014 compared between the ERA5 (left side) and GMFD (right side). The G_1_L-MME, G_2_I-MME, G_3_H-MME are the groups based on the size of the grid (sg) of the MME: low (≥ 0.8° sg ≤ 1.87°), intermediate (≥ 0.5° sg ≤ 0.7°), and high resolution (≥ 0.23° sg ≤ 0.35°), respectively

To show the spatial performance and avoid many very similar results for temperature indices, we selected the performance of MME for the TXx index (Fig. 3). The MMEs at different resolutions have greater skill than individual models in most regions, especially compared to the ERA5 (Figs. 2 and 3). Similar results are found when comparing MMEs and GMFD over most indices. For instance, KGE values for HadGEM3-GC31 in TXx over the NWS region in their low (G_1_L-MME; ≥ 0.8° sg ≤ 1.87°), intermediate (G_2_I-MME; ≥ 0.5° sg ≤ 0.7°), and high resolution (G_3_H-MME; ≥ 0.23° sg ≤ 0.35°) are 0.69, 066, and 0.73, respectively, and for the MMEs are 0.67, 0.65, and 0.70.

Thus, there is no clear relationship that increasing horizontal resolution always generates better performance results. In the case of TXx, the HadGEM3-GC31 family of models displays KGE values of 0.26, 0.43, and 0.45 for low, intermediate, and high resolution, respectively. On the other hand, contrasting values are found over the SAM that displayed KGE values of 0.55, 0.43, and 0.39 for the same resolution and family. Furthermore, as observed in TXx, the higher-resolution models do not improve the TNN simulation. For example, in their low and intermediate resolution, the KGE values in the NWS region for the MPI-ESM1-2 family are 0.55 and 0.44, respectively. However, the MRI-AGCM3-2 display considerable improvement for the same region with KGE in their intermediate (KGE = 0.07) and high resolution (KGE = 0.61).

Some performance problems in simulating absolute temperature extremes may be due to climate sensitivity, e.g., the increase in temperature in response to cloud feedback and cloud-aerosol interactions (Collazo et al. 2022). Other authors point out that CMIP6 shows a higher subregional climate sensitivity than CMIP5 (Seneviratne and Hauser 2020; Zelinka et al. 2020). However, this is not reflected in the performance of the ESMs to simulate percentile-based indices since these are generally calculated for each day concerning a long-term reference period (Almazroui et al. 2021c; Collazo et al. 2022). Therefore, an increase in warm days from annual analyses does not necessarily imply warming for the very warmest days of the year (Almazroui et al. 2021c).

**Fig. 4:**
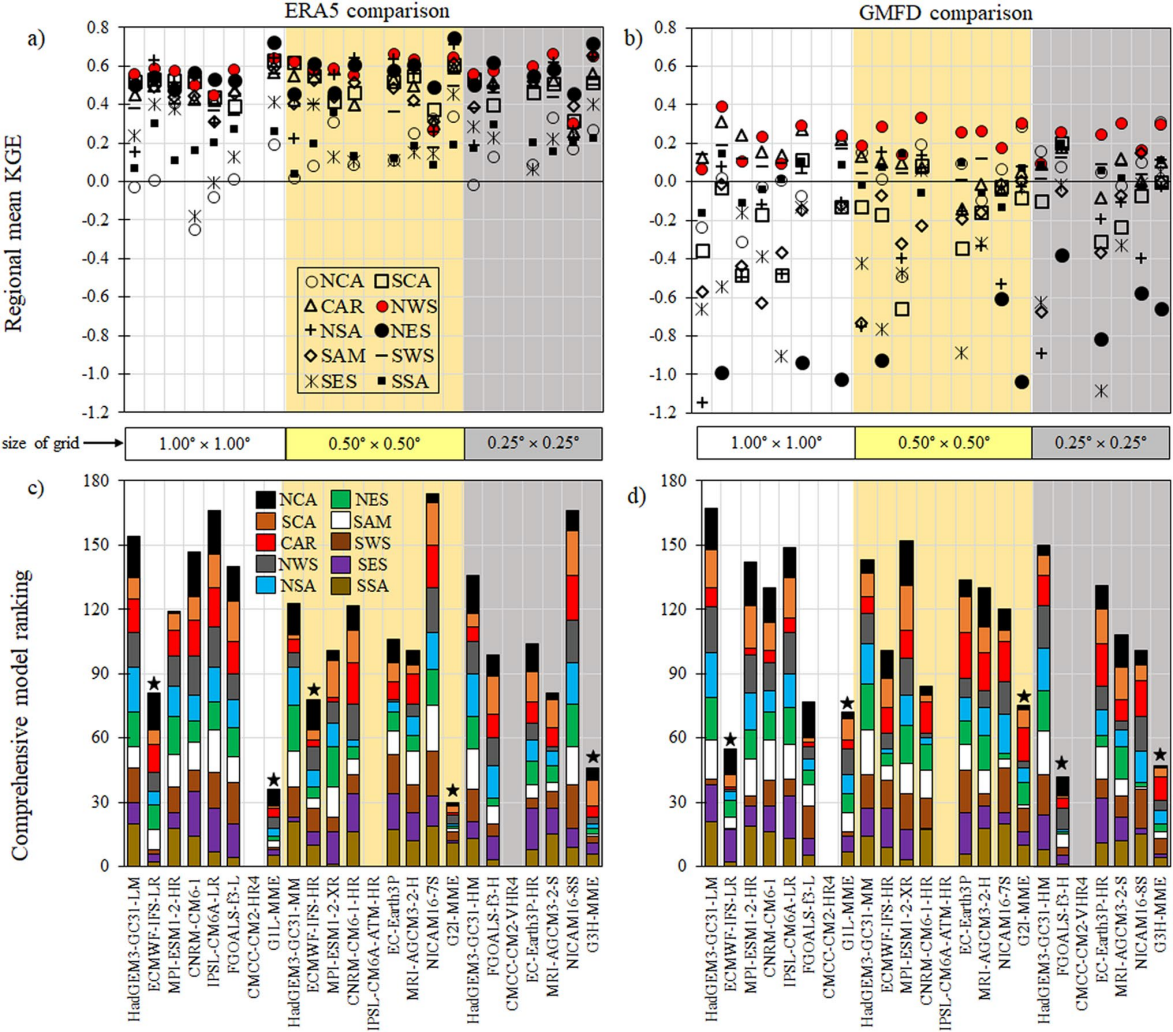
Comprehensive model ranking based on the regional mean KGE for all values in each extreme temperature indices between CMIP6 models and ERA5 (**a**, **c**) and GMFD (**b**, **d**) over the ten domains. The height of the color column in Figs. c and d represents the summation of each ranking. Thus, shorter columns indicate a better model or MMEs performance. White, yellow, and gray areas describe the G_1_L, G_2_I, G_3_H are the groups based on the resolution of the grid (sg) of the MME: low (≥ 0.8° sg ≤ 1.87°), intermediate (≥ 0.5° sg ≤ 0.7°), and high resolution (≥ 0.23° sg ≤ 0.35°), respectively. The symbols in (**a** and **b**) indicate a particular region shown in Fig. 1. The stars represent the 5 best performing ESMs or MMEs in (**c** and **d**). For comparison purposes in (**b**), values below − 1.2 were not plotted, such values were found for the NES region

Figure 4 shows an overall ranking used to select the model or MME that best represents the temperature indices in each region (Fig. 4a, b), where we considered the mean of KGE´s values in each extreme temperature index between CMIP6 models and ERA5 (a) and GMFD (b). In Fig. 4c, d, we observe that the consistently poorest performance for most indices is the NICAM16 and HadGEM3-GC31 families in their different horizontal resolutions. The best performance is obtained by using the G_2_I-MME, G_1_L-MME, and G_3_H-MME for the three resolutions, and for individual models, it is ECMWF-IFS-HR and ECMWF-IFS-LR. In this context, the MMEs improve the representation of temperature indices over most regions; however, this approach does not generate the best KGE values depending on the analyzed temperature index, region, and horizontal resolution. As noted in the individual performance model, our findings suggest that the resolution increase does not lead to systematic improvement of ESMs.

It is observed that the performance of the ESMs, simulating the temperature extremes, improves—albeit slightly—with the increase in horizontal resolution from each of the institutes. According to Roberts et al. (2019), Gutjahr et al. (2019), and Kodama et al. (2021), the improvements are attributed to reductions in the biases in the radiation components of the upper-atmosphere, adjustment to cloud forcing and, mainly, to the influence of the high resolution of the oceanic model coupled to the atmospheric model. This latter is particularly visible in the ECMWF models, where the coupled configuration showed a strong sensitivity to the increase in resolution of the NEMO (Nucleus for European Modeling of the Ocean) model, producing significant biases only in Australia and northern Europe (Roberts et al. 2018).

#### Trends in Temperature Indices

**Fig. 5:**
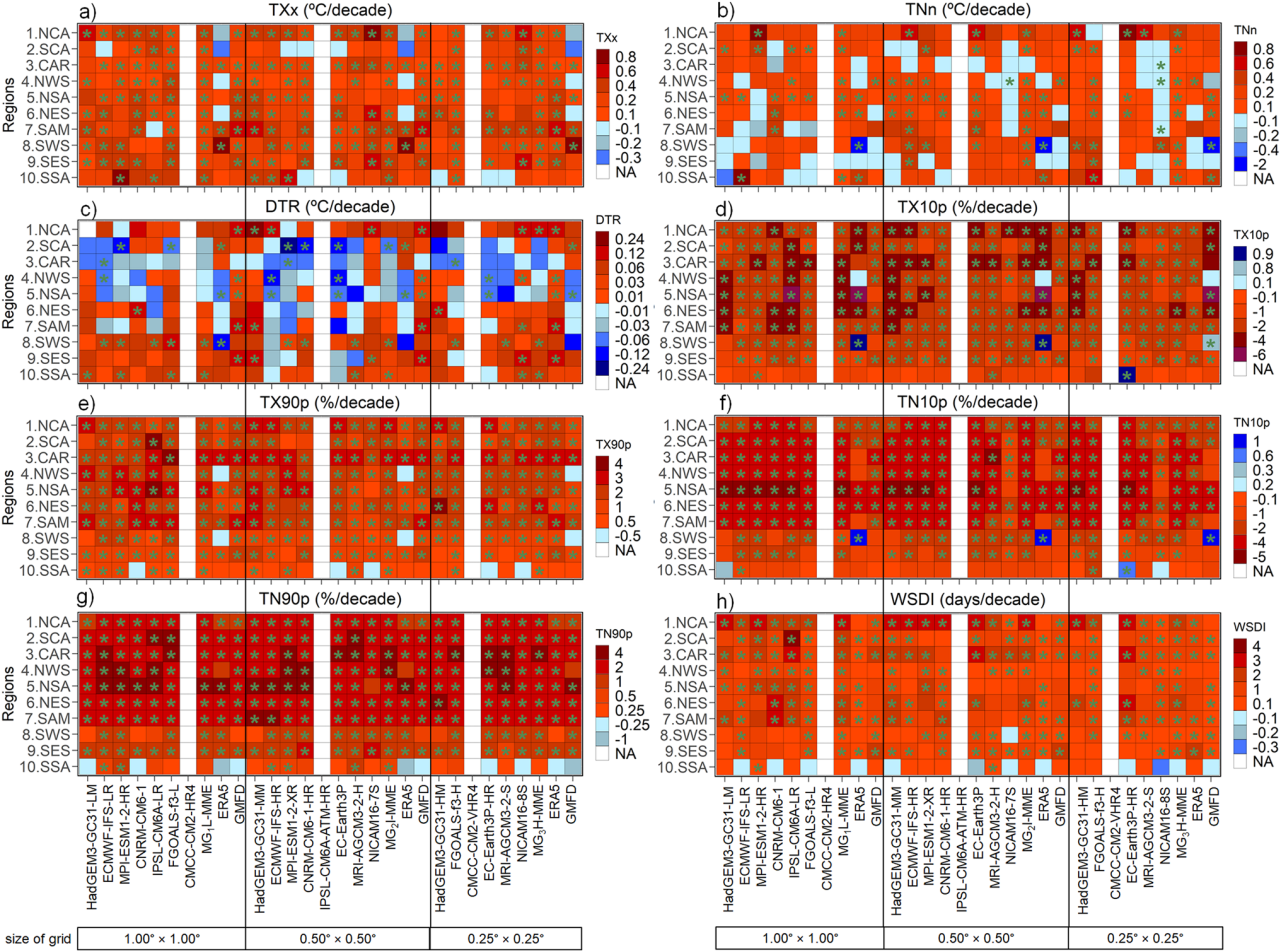
Decadal trends in temperature indices at the annual scale for individual ESMs, Multi-Models Ensembles (MMEs) and reference datasets during the 1981–2014 period. The NA (white boxes) indicates no temperature data available from the CMCC-CM2-HR4, IPSL-CM6-1-ATM-HR, and CMCC-CM2-VHR4 models. Boxes with significant trends at the 95% level have stars

Figure 5 shows the annual trends for temperature indices calculated for the observed datasets and ESMs during the 1981–2014 period. The warm days (TX90p), warm nights (TN90p), and warm spell duration (WSDI) indices show a positive trend over most Latin American regions for the three spatial resolutions groups and GMFD and ERA5 (Fig. 5e, g, h), corroborating the results found by Collazo et al. (2022). Though, in the SSA region, both observed datasets described no statistically significant negative trends for the TN90p and WSDI (only GMFD). Similarly, it was observed in the SWS and NWS regions for the TX90p (only GMFD).

The cold days/nights indices present decreasing trends over almost all regions and for all spatial resolution groups and observed datasets (Fig. 5d, f). The exception is in SWS and NWS regions, where positive trends are observed for these indices using the GMFD dataset.

The observational datasets exhibit high agreement around the decadal increase values of the hottest day index, excluding Central America (NCA and SCA), where the trend signals diverge. Using historical data set for 1961–2003 over SCA, Aguilar et al. (2005) find an increasingly significant trend of 0.3 °C per decade. The TNn is shown as an index of low statistical consensus between the observations over CAR and NES regions and the Amazon biome’s regions (SAM, NSA, NWS). This is reflected in significant differences between GMFD and ERA5 for the diurnal temperature range trends over the mentioned regions. Almeida et al. (2017) find general warming of the entire Brazilian Amazon region from meteorological station records; however, da Silva et al. (2019) concluded that there is still considerable uncertainty in the magnitudes and signals of the TNn and DTR trends due to the influence of continuous deforestation.

Regarding the temperature indices calculated from ESMs, the results show a general agreement for the TXx, TX10p, TX90p, TN10p, TN90p, and WSDI indices over most regions. However, SSA presents the greatest diversity of trend signals between TN90p and WSDI indices. Additionally, the SSA region exhibited in NICAM16 and EC-Earth3P models a decreasing trend for TX90p and an increasing trend for TN10p, contrary to the general pattern shown by the rest of the models and the observations. Similar behavior is found in Patagonia (SSA) by Rusticucci and Zazulie (2021) for TN10p during the austral summer (December-March) from CMIP5, while the trends agree with the other regions in the austral winter (June–August).

The DTR index displays high variability in trend signals between regions. On the one side, the tropical regions (SCA, CAR, NWS, NSA, and NES) tend to show a decreasing trend, coinciding with the GMFD values. On the other hand, the extratropical regions (NCA, SAM, SWS, SSA) show an increasing trend, like ERA5. This lack of the model’s ability to capture the DTR index can be due to the fact that it is strongly affected by land surface conditions, which are very heterogeneous within the model´s grid cells and are transitory in time; results that coincide with the findings of Avila-Diaz et al. (2020a).

Overall, these results indicate warmer conditions during the day and night, an increase in the duration of warm episodes over most of South America, and consistently a decrease in cold days and nights, which is in line with the others (Collazo et al. 2022; Gouveia et al. 2022). In general, the MMEs are adequate in representing the observed trends for each index in the regions of South America on an annual time scale, except for DTR, where the most remarkable inconsistencies are present. Moreover, our results suggest that there is no relationship between spatial resolution and trends since we find similar trend values between the high-, intermediate-, and low-resolution simulations for each ESM.

#### Performance Evaluation in Precipitation Indices

**Fig. 6:**
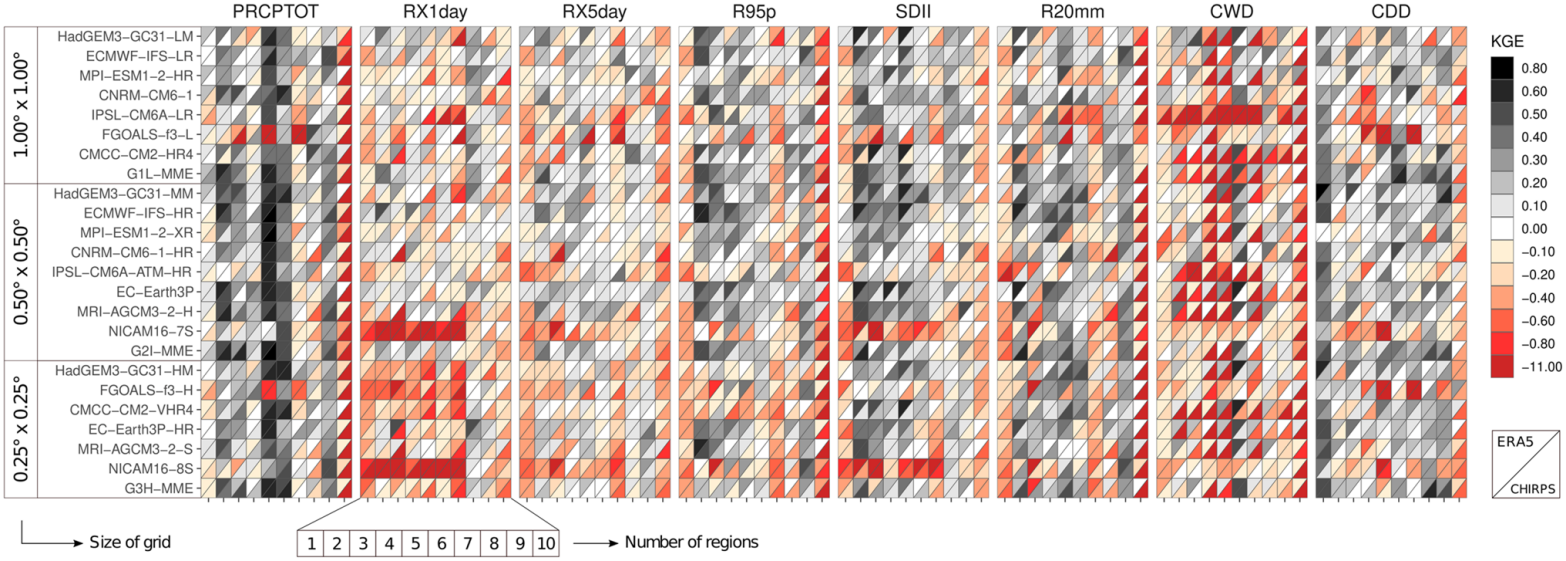
KGE Performance evaluation obtained of ESMs to estimate annual precipitation indices compared to ERA5 and CHIRPS datasets during 1980–2014. The number of regions are the ID according to Fig. 1:

**Fig. 7:**
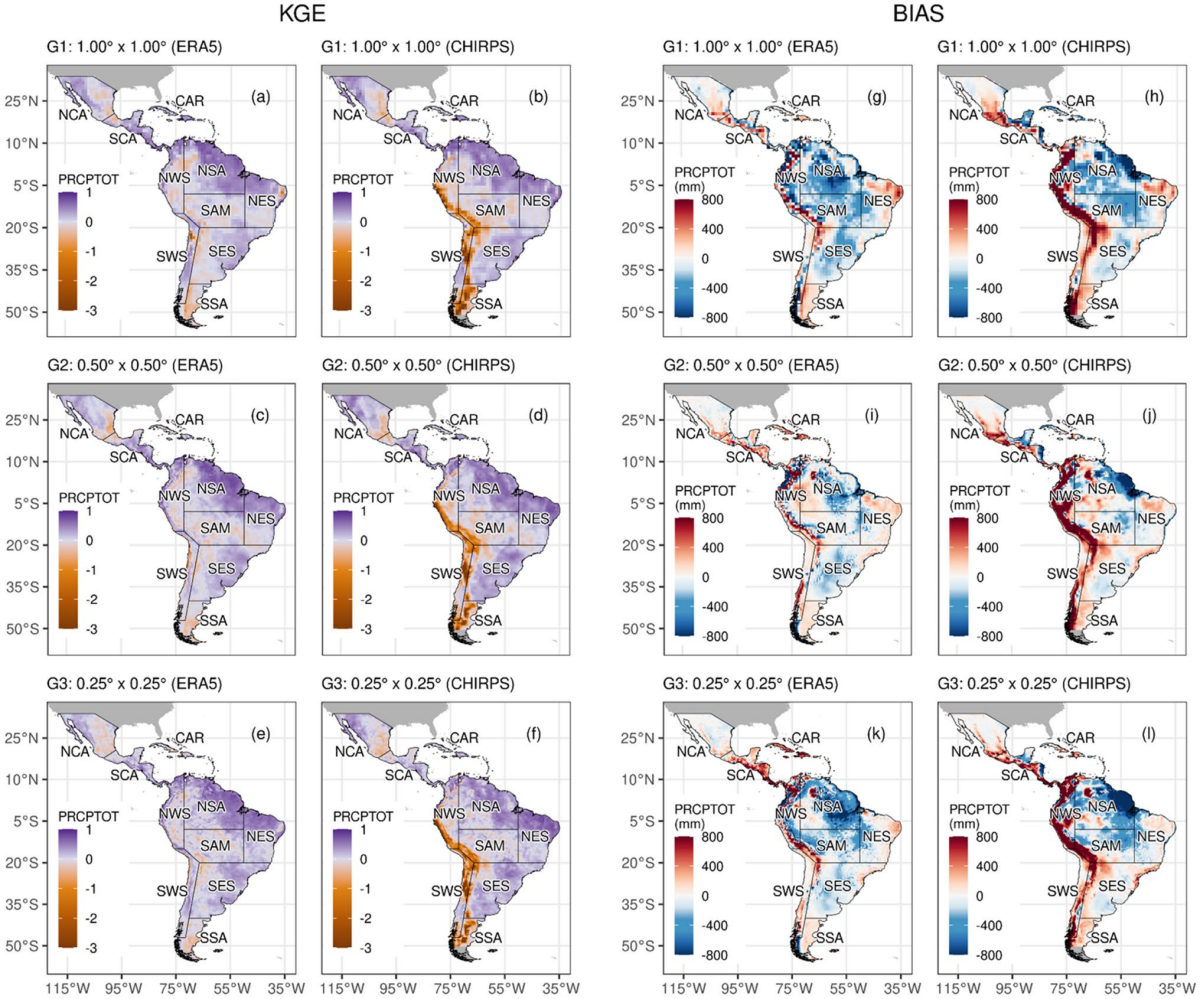
The KGE (**a**–**f**) and climatology bias (**g**–**l**) of multi-model ensemble (MME) for PRCPTOT during 1981–2014 compared between the ERA5 (left side) and  CHIRPS (right side). The G_1_L-MME, G_2_I-MME, G_3_H-MME are the groups based on the size of the grid (sg) of the MME: low (≥ 0.8° sg ≤ 1.87°), intermediate (≥ 0.5° sg ≤ 0.7°), and high resolution (≥ 0.23° sg ≤ 0.35°), respectively

The skill of the ESMs to simulate the extreme climate precipitation indices are shown in Fig. 6. For the sake of brevity, we selected the annual total wet-day precipitation (PRCPTOT; Fig. 6, first column) index to show the performance of MMEs in the three resolution groups (e.g., low, intermediate, and high; see also Fig. 7).

The KGE values of PRCPTOT tend to be more consistent with the ERA5 dataset than with CHIRPS (Figs. 6 and 7). Furthermore, the models compare poorly in most regions, with KGE values close to 0.0, except for SCA, NSA, NES, and SES regions with KGE values above 0.4. For instance, in these latter regions, MRI-AGCM3-2-H, EC-Earth3P, and CNRM-CM6-1 models show BR and RV close to 1 with a CORR coefficient above 0.45 (Fig. S2 shows the values of this KGE component). Notably, the FGOALS, in their low and intermediate horizontal resolution, display extremely negative KGE values for most regions. Finally, it should be noted that FGOALS-f3-L and FGOALS-H are not independent and agree on the sign and magnitude of the biases, showing dry biases for almost all regions (Fig. S2).

The ESMs display low performance over most regions in simulating intensity indices such as RX1day, RX5 day, R95p, and SDII (see columns second to fourth of Fig. 6). Especially FGOALS and NICAM16 families of models show insignificant correlation, wet biases, and KGE values above 0.0 for most regions in those intensity indices. However, on average, for RX1day, HadGEM3-GC31-LM and ECMWF-IFS-HR show the best relative performance, while for RX5day and R95p, the EC-Earth3P model shows the best results. The ESMs from HighResMIP continue to present difficulties in simulating the SDII, especially for NCA, SAM, SWS, SES, and SSA compared with the simulation from CMIP5/3 simulations (e.g., Sillmann et al. 2013). Moreover, we find that the performance of ESMs depends on the reference dataset used; a similar result is highlighted by recent studies (Akinsanola et al. 2021; Ngoma et al. 2021) using CMIP6 models. For instance, independent of horizontal resolution, when ERA5 and CHIRPS are used for comparison, the ESMs showed wet and dry bias, respectively.

The very heavy precipitation days (R20 mm; Fig. 6, sixth column) are not well captured in most models. However, EC-Earth3P has the best performance across most regions, with KGE values between 0.21 and 0.67, except for NCA, SAM, SWS, and SSA areas. In the case of the IPSL-CM6A and NICAM families, a relatively poor performance was observed in the CAR and SAM regions, with KGE values varying from − 2.88 to − 0.15.

Regarding the precipitation duration indices, the ESMs poorly represent consecutive wet days (CWD; Fig. 6, seventh column) against both reference datasets in almost all regions, except for the HadGEM3-GC31 model family, which shows the best performance in the NSA, NSE, and SAM regions (0.19 ≥ KGE ≤ 0.76). It is important to note that the IPSL-CM6A (− 3.55 ≥ KGE ≤ 0.30) and FGOALS-f3-L (− 0.55 ≥ KGE ≤ 0.21) families of models display the worst performance over the study region compared to ERA5 and CHIRPS. Moreover, the ESMs still exhibit a significant overestimation of CWD in all subregions except for SSA. This known drizzle bias has persisted since the ESMs generation from CMIP3 (Faye and Akinsanola 2022; Medeiros et al. 2022).

The ESMs capture the climate patterns of consecutive dry days (CDD; Fig. 6, eighth column) only over the NCA, with KGE values above 0.3, except for MPI-ESM1-2-HR, MPI-ESM1-2-XR, and NICAM16-7S. Notable good performance is obtained for the HadGEM3-GC31 family of models in the three different resolutions (0.11 ≥ KGE ≤ 0.82). Nevertheless, the FGOALS-f3 family of models presents the minimum negative values of KGE that vary between − 3.10 and − 0.43 over the northern part of South America (e.g., NSA, NWS, and SAM regions).

Significant uncertainty exists in estimating rainfall and evaluating extreme precipitation indices related not only to model sensibility in estimating daily rainfall but also to the thresholds defined in the indices and the regions considered. Therefore, the better performance for CDD and worse for CWD may be related to these features. For example, studies show that a threshold of daily rainfall less than < 1 mm for arid and desert climate characteristics can be considered adequate. Conversely, by considering a threshold of > 1 mm for CWD over regions of high convection activity and high daily precipitation variability, as the Amazon Basin (NSA and SAM) and the Choco region (in NWS) in Colombia, will generate that almost all days with rainfall will be considered over this threshold (Espinoza et al. 2019; Marengo et al. 2011; Villar et al. 2009).

**Fig. 8:**
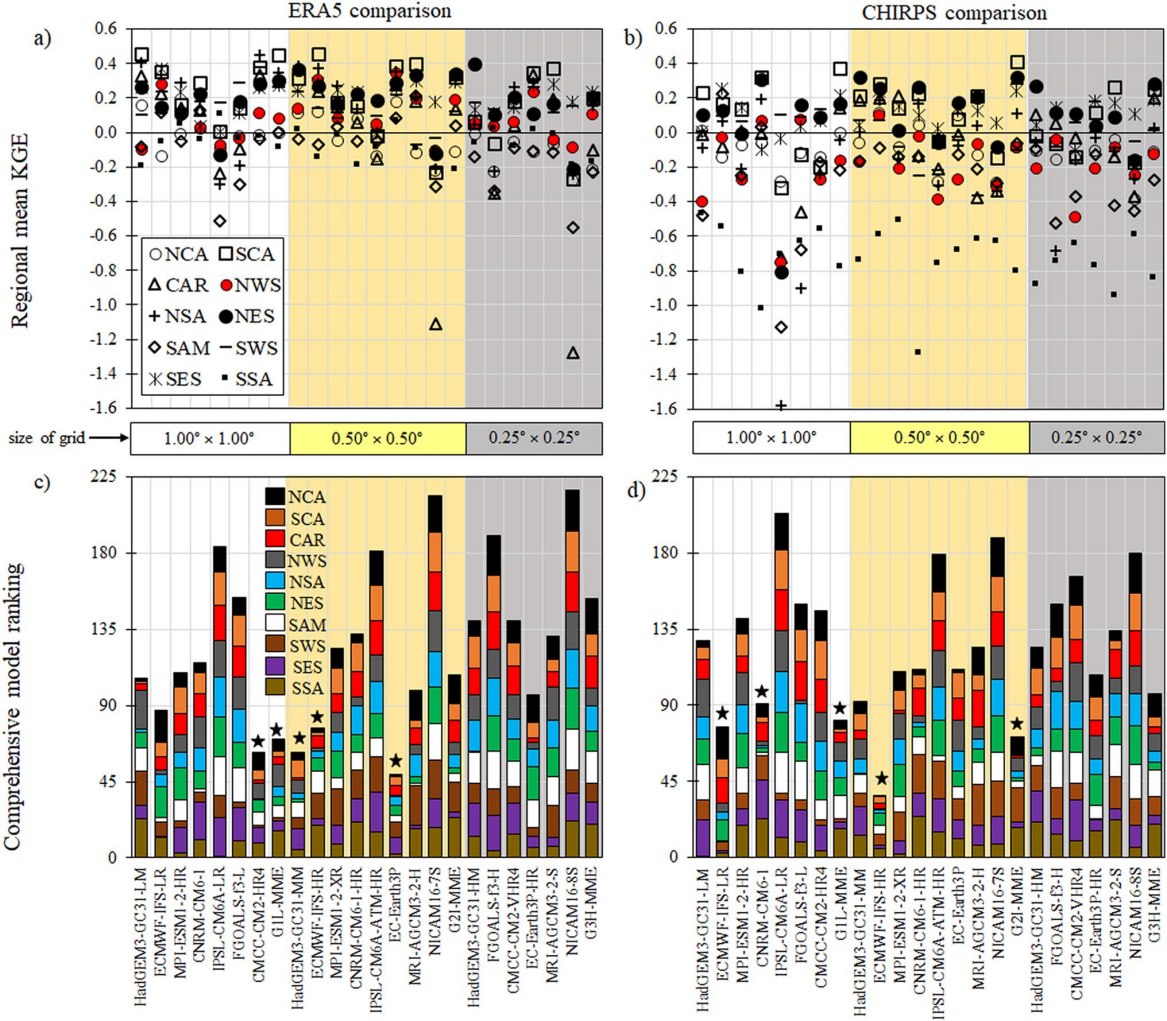
Comprehensive model ranking based on the regional mean of KGE for all precipitation indices between CMIP6 models and ERA5 (**a**, **c**) and CHIRPS (**b**, **d**) over the ten domains. The height of the color column in (**c** and **d**) represent the summation of each ranking. Thus, shorter columns indicate a better model or MMEs performance. White, yellow, and gray areas describe the G_1_L, G_2_I, G_3_H are the groups based on the resolution of the grid (sg) of the MME: low (0.8° ≤ sg ≤ 1.87°), intermediate (0.5° ≤ sg ≤ 0.7°), and high resolution (0.23° ≤ sg ≤ 0.35°), respectively. The symbols in (**a** and **b**) indicate a particular region shown in Fig. 1. The stars represent the 5 best performing ESMs or MMEs in (**c** and d)

Like the temperature indices (Sect. 3.1.1), we analyze the overall performance of individual models and MMEs. The best performance is found for EC-Earth3P, CMCC-CM2-HR4, HadGEM3-GC31-MM, G_1_L-MME, and ECMWF-IFS-HR when compared with ERA5 (Fig. 8a–c). However, when CHIRPS is used as a reference dataset, the best models are ECMWF-IFS-HR, G_2_I-MME, ECMWF-IFS-LR, G_1_L-MME, and CNRM-CM6-1 (Fig. 8b, d). For this reason, G_1_L-MME and G_2_I-MME, followed by ECMWF-IFS-HR, are good alternatives to capture climate precipitation extremes. Our results are consistent with Liang-Liang et al. (2022), who find the best relative performance for the HighResMIP multi-model ensemble and ECMWF-IFS-LR in capturing the precipitation frequency's climatological (1961–1990) distributions over central Asia.

The individual CMIP6 models and the MMEs presented relatively higher KGE values when compared with ERA5 than CHIRPS for most indices in the study areas. However, overall our results indicate no clear relationship between the increase in horizontal resolution and improved performance of the precipitation indices, which is coherent with the results of Bador et al. (2020), who show a global performance analysis of precipitation extremes with CMIP6 models. For example, when ERA5 is used as a reference dataset for the CDD index, the magnitudes of KGE for MMEs in their low, intermediate and high resolution are 0.42, 046, and 0.54 over the NCA region, respectively, and 0.49, 0.82, and 0.69 for the HadGEM3-GC31 models. Finally, the selection of the best models depends on the region, index, and reference dataset used.

When the resolution of the ESMs from each institute is compared individually, we find highly different results. In the case of the models of the CMCC, CNRM-CERFACS, EC-Earth-Consortium, IAP-CAS, NERC-MOHC, IPSL, MPI, MRI, and MIROC Institutes, the increase in resolution results in lower or almost equal performance, similar to Scoccimarro et al. (2022) and Liang-Liang et al. (2022). Only the ECMWF models result in a significant increase in performance coincident with an increase in spatial resolution (Bador et al. 2020). According to Roberts et al. (2018), a higher resolution in the ocean–atmosphere coupling improves the teleconnections associated with the precipitation events simulated by the ECMWF model.

#### Trends in Precipitation Indices

**Fig. 9:**
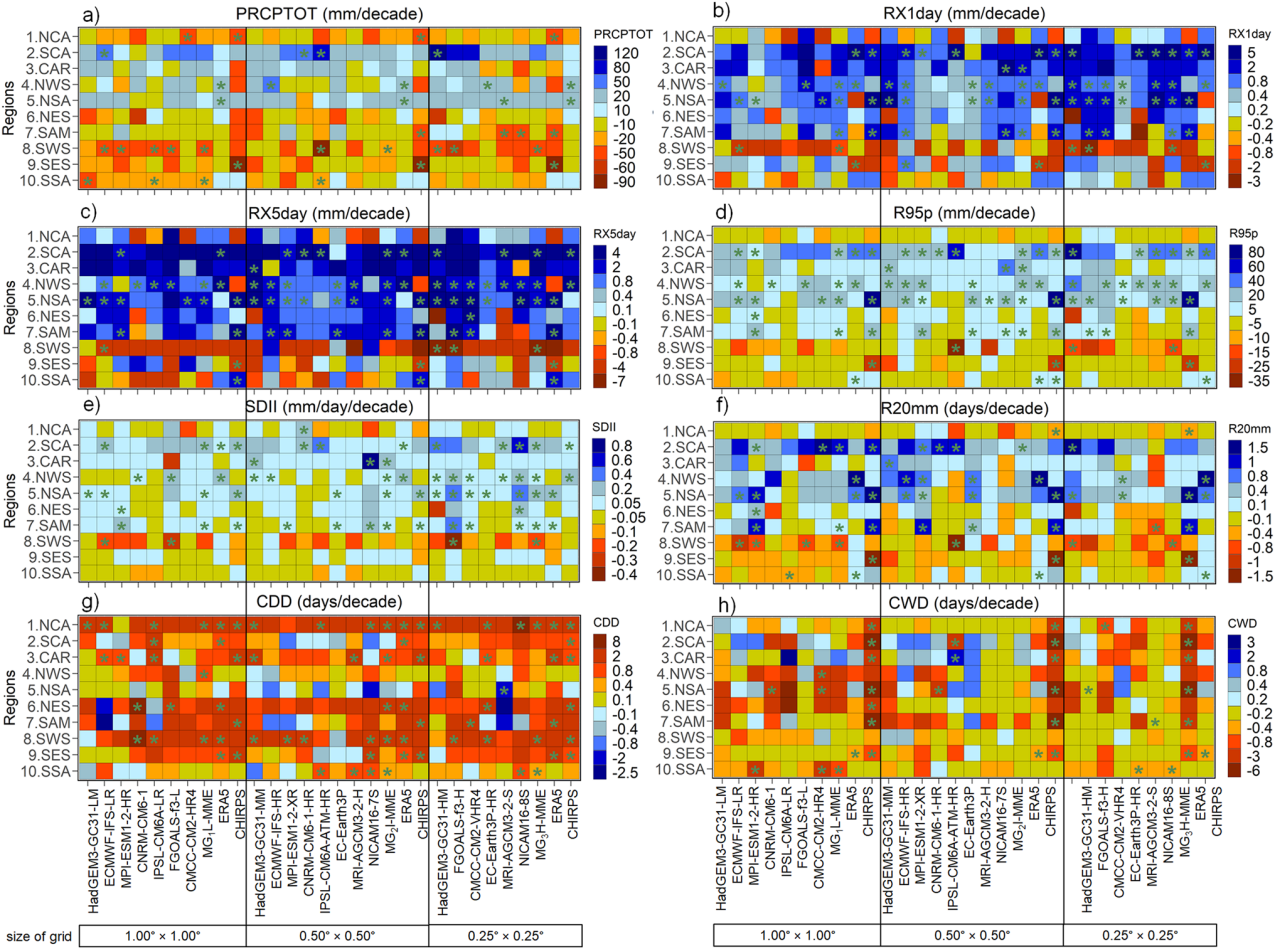
Decadal trends in precipitation indices at the annual scale for individual ESM, multi-model ensembles (MMEs) and reference datasets during the 1981–2014 period. Boxes with significant trends at the 95% level have stars

The annual precipitation trends computed for the reference datasets and the ESMs between 1981 and 2014 are shown in Fig. 9. The precipitation indices generally show different magnitudes and trend signals among regions and models when compared with temperature indices, indicating greater complexity. The PRCPTOT in ERA5 and GMFD datasets exhibit significantly mixed trends (i.e., positive and negative) in the CAR, NWS, and NES regions. Meanwhile, the SDII presents differences for these same regions, in addition to NCA, SAM, SWS, SES, and SSA. The PRCPTOT index only shows significant positive trends for CHIRPS in the NWS and NSA regions, which are consistent with the results of the MRI-AGCM3-2-S and EC-Earth3P-HR models in the 0.25° resolution grid.

In tropical regions (SCA, CAR, NWS, NSA, NES, and to a lesser extent SAM), models show strong agreement with observations regarding increases in RX1day, RX5day, R95p, and R20mm. In contrast, the extratropical regions show the greatest discrepancies with observed trends. This pattern persists in the results of the ESMs, showing greater variability in the trend signals in the extratropical regions than in the tropical ones. Bador et al. (2020) find, on the other hand, that in the extratropical regions of the northern hemisphere, the models agree better with the observations than in the tropical regions. This may be related to greater data records for calibrating ESMs across the northern hemisphere.

The increase of PRCPTOT in the NWS region is consistent with the increasing trends of RX1day, RX5day, R95p, SDII, and R20mm (Fig. 8b–f), which are also significant and consistent in several models at the three resolutions analyzed. In addition, significant positive trends for RX1day, RX5day, and R95p are observed in the SCA region for CHIRPS and ERA5 (Fig. 8b–d) at all three resolutions; however, only the 0.5° and 0.25° multi-models show significant positive trends. A larger number of 0.25° resolution models generally show better coherence with observed trends for RX1day and 0.5° resolution models for RX5day and R95p.

The CDD index shows significant positive trends in the NCA and SWS regions for CHIRPS and ERA5, which are consistent across most models at all three resolutions, including the MME in each group. The most considerable inconsistencies are present in CWD (Fig. 8h). Although ERA5 shows significant negative trends in most regions, except SWS, SES, and SSA, the models fail to represent such a trend; only the 1.0° resolution for CMCC-CM2-HR4 and CNRM-CM6-1 models showed consistencies in the NWS and NSA region. It can also be observed that the 1.0° resolution MME shows negative trends like those of ERA5; however, they are not significant.

Similar to the temperature indices of Bador et al. (2020), the increase in spatial resolution does not influence the values and signals of the precipitation trends, except for specific cases without statistical significance. Finally, the indices display a general increase in rainfall in regions of northern South America and southern Central America, while in NES, NCA, and southern regions of South America, rainfall rates decrease. However, it is also evident that some drought events in the NWS, NSA, and SAM regions (Amazon region) have also increased; yet the temperature indices' changes were more significant than the precipitation indices in these regions. On the other hand, the models and the observations show high consistency in increasing drought events in the NES region. All of the above align with previous studies (Almeida et al. 2017; da Silva et al. 2019; Olmo and Bettolli 2021; Solman et al. 2021; Medeiros and Oliveira 2022).

### Future Projections for the 2021–2050 Period Under Scenario SSP5-8.5

#### Temperature Projections

**Fig. 10:**
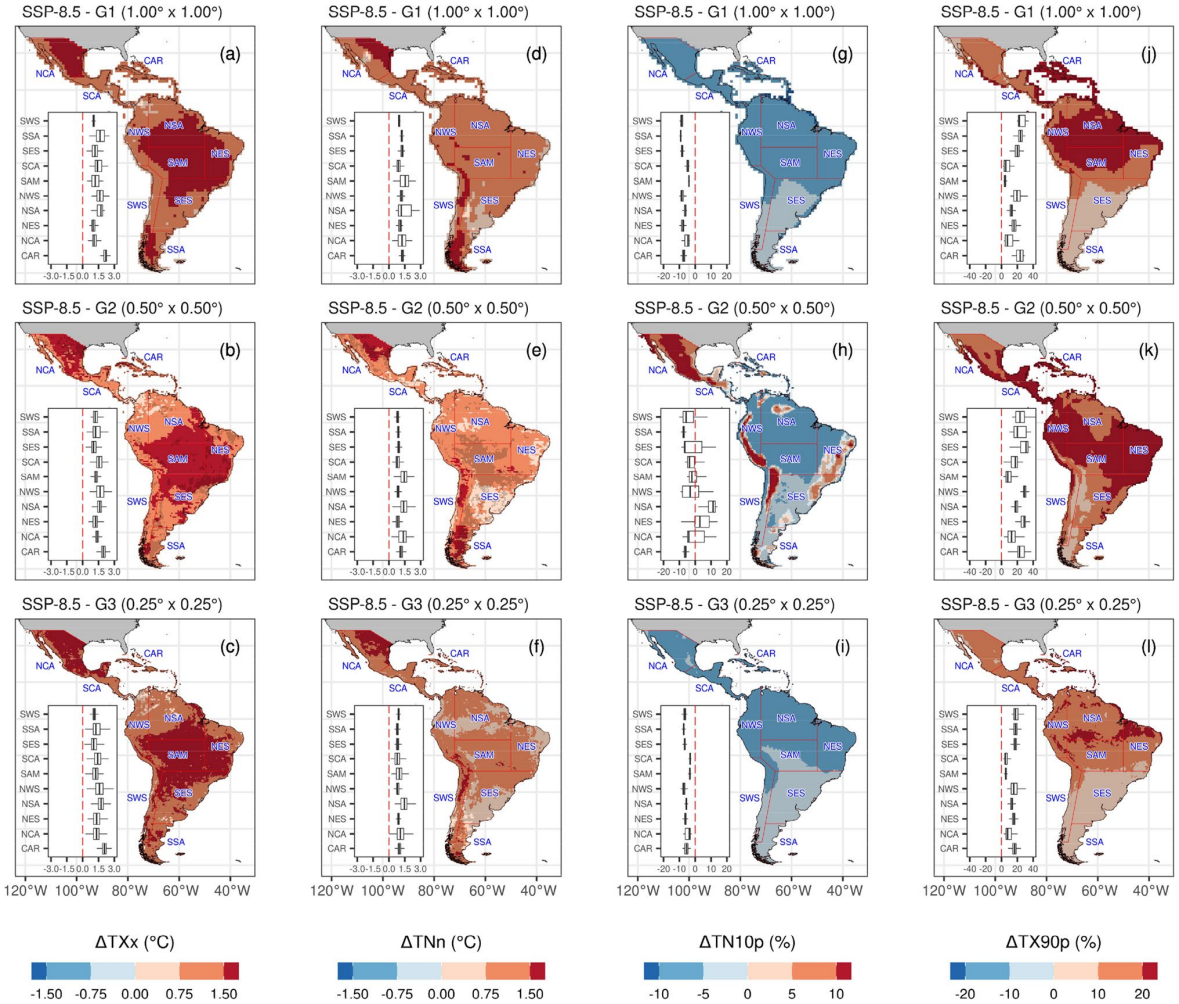
Future changes of multi-model ensemble in temperature extremes for TXx (**a**–**d**), TNn (**d**–**f**), TN10p (**g**–**i**), and TX90p (**j**–**l**) indices under SSP5-8.5 scenario for 2021–2050 relative to the reference period (1981–2010)

**Fig. 11:**
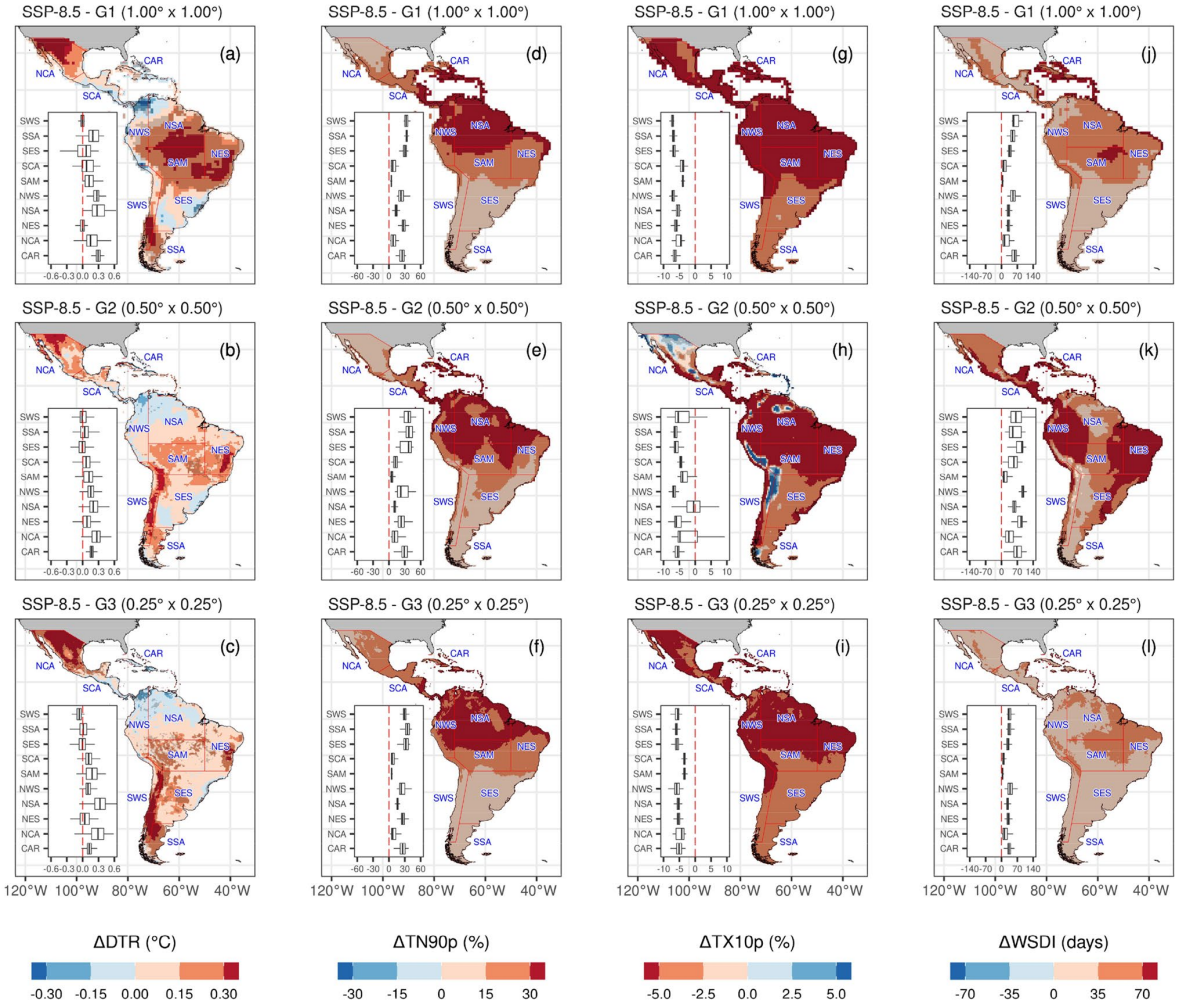
Future changes of multi-model ensemble in temperature extremes indices DTR (**a**–**c**), TN10p (**d**–**f**), TX10p (**g**–**i**) and WSDI (**j**–**l**) under SSP5-8.5 scenario for 2021–2050 relative to the reference period (1981–2010)

Figures 10 and 11 illustrate the regional and spatial changes in temperature indices for 2021–2050 compared to the baseline period (1981–2010) for each MME group. Following the trends from 1981 to 2014, future MME projections describe warmer conditions over most regions and resolutions. Though, between resolutions, a behavior change is observed in the spatial change over the MME-G_2_I compared to the other two MME groups. G_1_L-MME and G_3_H-MME show a consistent reduction of cold nights and days over all the regions, reaching the greatest magnitude near the Equator over NSA and NWS regions (< − 6.6% for TN10p and < − 5.7 for TX10p), and over the CAR region (< − 6.7% for TN10p and < − 5.9 for TX10p). However, TX10p shows an increase, mainly over Brazil’s Andes and sub-tropical coast. This increase is also noted for TN10p and spatially extends into the Sierra Madre in Mexico and Central America. Considering the mean of the regions at the intermediate resolution, TN10p has an increase of 4% days in the SCA region and more than 10% days in the NCA region. Noteworthy, this was not observed when comparing the trends from 1981 to 2014 (Fig. 5). This change detected in these regions deserves a more comprehensive explanation. Whereas MME-G_2_I includes the CNRM-CM6-1-HR, EC-Earth3P, HadGEM3-GC31-MM, MRI-AGCM3-2-H, and NICAM16-7S models, some of these are not included in the other two groups (see Table 2). Ortega et al. (2021) evaluate 33 models, MME for the CMIP6 and the best 6-models from CMIP6 in Central and South America, listing the best models representing the annual cycle and performance against ERA5 data. From the models in MME-G2I, only MIROC is included in this list of best models. However, even for the Andes, the same study describes that EC-Earth3-Veg and MRI-ESM2-0 have a good performance for temperature.

Absolute (TXx, TNn) and percentile-based threshold indices (TN90p and TX90p) increase over most regions and the three MME groups. TXx describes an increase that reaches nearly 2.0 °C over the SAM region, followed by NCA with an increase of 1.6 °C. TNn shows values greater than 1.5 °C over the north of NCA and the Pacific slope of the Andes within the three resolution groups. Nevertheless, in South America, the largest increase (> 1.4 °C) is found across the southeast region (SSA). Since the MMEs show poorer performance from 1981 to 2014 (Fig. 5), caution must be used when analyzing the results of this region. In the case of TN90p and TX90p, the three MME groups show spatially inconsistent results over South America, particularly over the Amazon Basin. In comparison, TN90p shows an increase of more than 20% of warm nights over the northeast Amazon Basin, with a spatial inconsistence in MME-G_2_I. TX90p describes a similar percentage increase over the central Amazon Basin, extending to other regions in MME-G_2_I: NWS, NES, SES, CAR, and SCA. Interestingly, the MME-G_3_H shows the greatest percentage change over the Amazon River channel and floodplain, which could be related to the thermal characteristics of the water compared to the surrounding landmass. WSDI, as the previous percentile-bases threshold indices, displays spatial inconsistencies over most of South America between the three resolution MME groups. This is more evident in the Amazon Basin, the Caribbean, and Central America. Unrealistic land–atmosphere interactions and misrepresentation of the Amazon evapotranspiration, at least for half of all CMIP5 models (Baker et al. 2021), which may also be found in CMIP6 models, could be one of the reasons for the spatial inconsistencies. While in the Caribbean and Central America region, this would be related to misrepresentation of land-oceanic interaction.

The diurnal temperature range illustrates contrasting changes among regions. An increase is observed over the three MMEs for NCA and SWS regions in the range of 0.2–0.3 °C, and yet it shows an increase over the Amazon Basin (SAM, NES, and NSA regions). This increase is more remarkable for the MME-G_1_L. Noteworthy, only for the DTR contrasting changes are observed when comparing the Caribbean and Pacific region of Central America (Durán-Quesada et al. 2020); more evident in the coarsest spatial resolution. The warmer conditions over most regions coincide with an increase in WSDI. However, its spatial representation varies in MME-G_2_I compared to the other two MMEs. While the NES region increases nearly 39 days and 50 days in the 2021–2050 period compared to 1981–2010 for the highest and lowest resolution, the intermediate resolution describes an increase of nearly 94 days. The highest increase over the MME-G_2_I is also evident for the rest of the regions, but it is highest for SCA and the northwest and northeast South America. Consider that these two indices had the lowest performance between the MMEs in most regions compared to the 1981–2014 period.

**Fig. 12:**
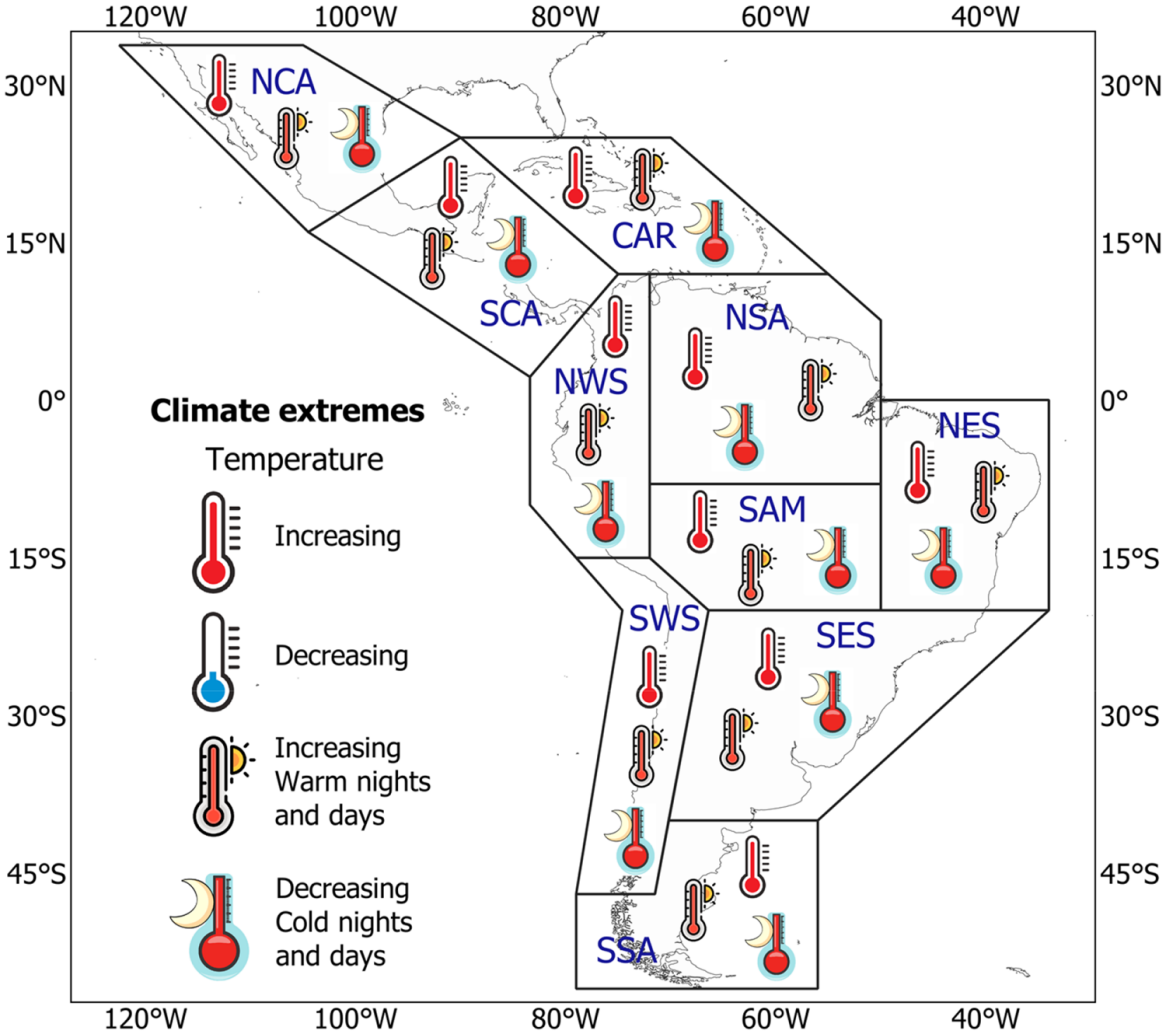
Summary of the projected changes in temperature climate extremes for each Latin America and Caribbean reference region for 2021–2050 under the SSP5-8.5 scenario

Overall, our results with the CMIP6-MME for all the regions and resolutions indicate a warmer future, which is consistent with the results described by the IPCC AR6 (IPCC 2021) and previous works on Latin America and the Caribbean (Almazroui et al. 2021b, a; Lovino et al. 2021; Ortega et al. 2021; Seneviratne et al. 2021). The summary of the main projected changes expected for temperature climate extremes is depicted in Fig. 12 and Table 3. Though, as observed for G_2_I-MME, spatial representations indicate a reduction of the models' performance in representing atmospheric conditions related to the diurnal temperature range and dry spell duration. The different magnitudes in MME across the three resolutions can be explained as they do not account for the same models in the ensemble mean calculation (Table 2). For instance, from G_2_I- MME and for the NCA, SCA, and CAR regions, Almazroui et al. (2021b) find that the largest surface air temperature negative bias, when compared to the Climatic Research Unit (CRU) (1995–2014), is the CNRM-CM6-1-HR, which is included in the lowest and intermediate horizontal resolution MME groups. On the other hand, the CRU dataset has shown a satisfactory performance compared to other temperature datasets, at least for the NCA and SCA regions (Cavazos et al. 2020).

**Table 3:** Projected changes over the 2021–2050 period in temperature indices relative to the reference period (1981–2010) for the Multi-Model Ensemble for each resolution group (G_1_L, G_2_I, G_3_H) over analysis regions under the SSP5-8.5 scenario

Group Resolution	Indice	Unit	NCA	SCA	CAR	NWS	NSA	NES	SAM	SWS	SES	SSA
G_1_L 1.0° × 1.0°	TXx	°C	1.63	1.03	1.04	1.17	1.66	1.65	**2.07**	1.12	1.47	1.2
	TNn	°C	1.47	1.05	0.97	1.23	1.21	1.16	1.27	1.24	0.9	**1.49**
	DTR	°C	**0.3**	− 0.01	− 0.01	0.03	0.19	0.27	0.29	0.18	0.08	0.14
	TN10p	% of days	− 6.34	− 7.89	− 8.61	− 8.22	**− 9.08**	− 8.19	− 7.33	− 5.27	− 4.74	− 3.98
	TN90p	% of days	13.63	28.07	33.17	29.56	**33.78**	24.24	24.49	8.57	8.55	4.45
	TX10p	% of days	− 5.48	− 6.17	**− 7.24**	− 6.64	− 6.92	− 6.94	− 6.37	− 5.16	− 4.11	− 3.9
	TX90p	% of days	12.76	16.17	**25.69**	19.85	23.43	19.51	22.97	9.6	6.94	4.57
	WSDI	days	31.33	32.08	**63.38**	38.38	49.22	50.26	54.97	20.65	13.9	5.35
G_2_I 0.5° × 0.5°	TXx	°C	1.59	1.2	1.25	1.06	1.31	1.66	**1.94**	1.37	1.58	1.25
	TNn	°C	**1.41**	0.83	0.81	0.93	0.89	0.89	1.15	1.3	0.84	1.42
	DTR	°C	**0.21**	0.07	0.02	− 0.02	0.03	0.16	0.17	0.25	0.07	0.11
	TN10p	% of days	**10.23**	4.08	− 3.93	− 1.75	− 6.5	− 2.39	− 5.3	− 0.23	− 1.79	− 0.97
	TN90p	% of days	11.22	23.56	35.55	32.84	**37.52**	26.5	29.42	12.13	12.82	5.76
	TX10p	% of days	− 0.61	− 5.02	− 3.62	− 4.61	− 5.73	**− 6.67**	− 4.88	− 2.14	− 3.99	− 3.06
	TX90p	% of days	18.85	27.21	24.2	28.11	22.69	**29.43**	24.08	13.63	16.45	9.31
	WSDI	days	57.2	80.15	67.08	78.68	58.36	**94.22**	69.44	37.38	49.97	19.38
G_3_H 0.25° × 0.25°	TXx	°C	1.72	1.33	1.09	1.09	1.33	1.59	**2.04**	1.29	1.42	1.2
	TNn	°C	**1.47**	0.9	0.91	0.85	0.8	0.85	1.02	1.09	0.8	0.98
	DTR	°C	**0.33**	0.03	− 0.07	− 0.01	0.01	0.12	0.13	0.27	0.12	0.17
	TN10p	% of days	− 5.61	− 6.25	− 6.71	− 6.6	**− 7.23**	− 7.12	− 5.5	− 4.51	− 3.4	− 3.39
	TN90p	% of days	15.98	26.5	30.29	31.63	**34.65**	25.24	25.63	9.11	7.68	5.19
	TX10p	% of days	− 5.33	− 5.35	− 5.93	− 5.74	**− 5.95**	− 5.73	− 5.09	− 4.73	− 3.47	− 3.38
	TX90p	% of days	12.83	15.58	19.54	17.24	**17.51**	15.91	16.13	9.26	5.75	5.56
	WSDI	days	27.16	30.37	**41.18**	28.46	35.35	38.53	33.69	17.23	9.4	4.6

Values in bold represent the highest change for each index

#### Precipitation Projections

**Fig. 13:**
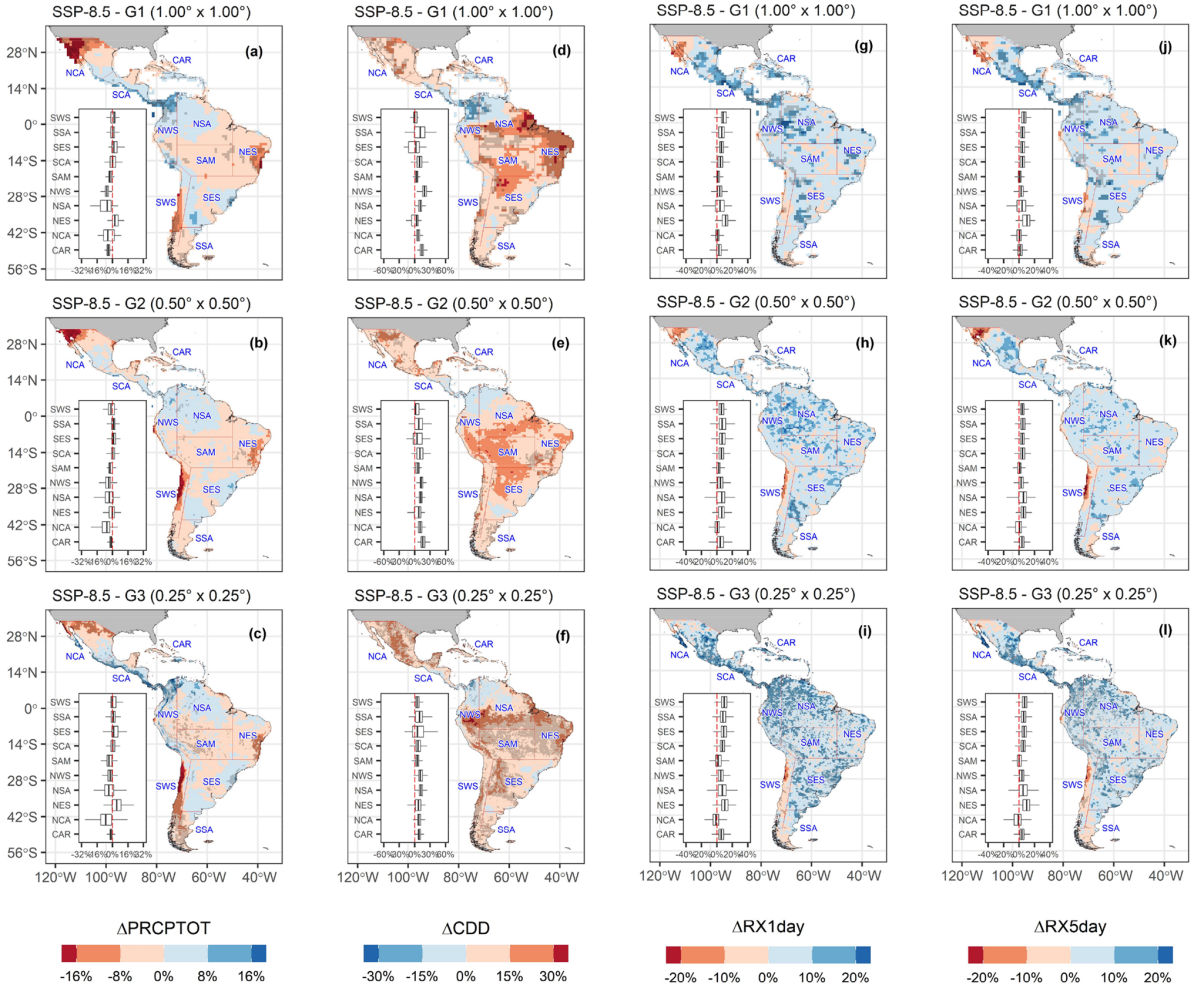
Future changes of multi-model ensemble in precipitation extremes indices PRCPTOT (**a**–**c**), CDD (**d**–**f**), RX1day (**g**–**i**), and RX5day (**j**–**l**) under SSP5-8.5 scenario for 2021–2050 relative to the reference period (1981–2010)

**Fig. 14:**
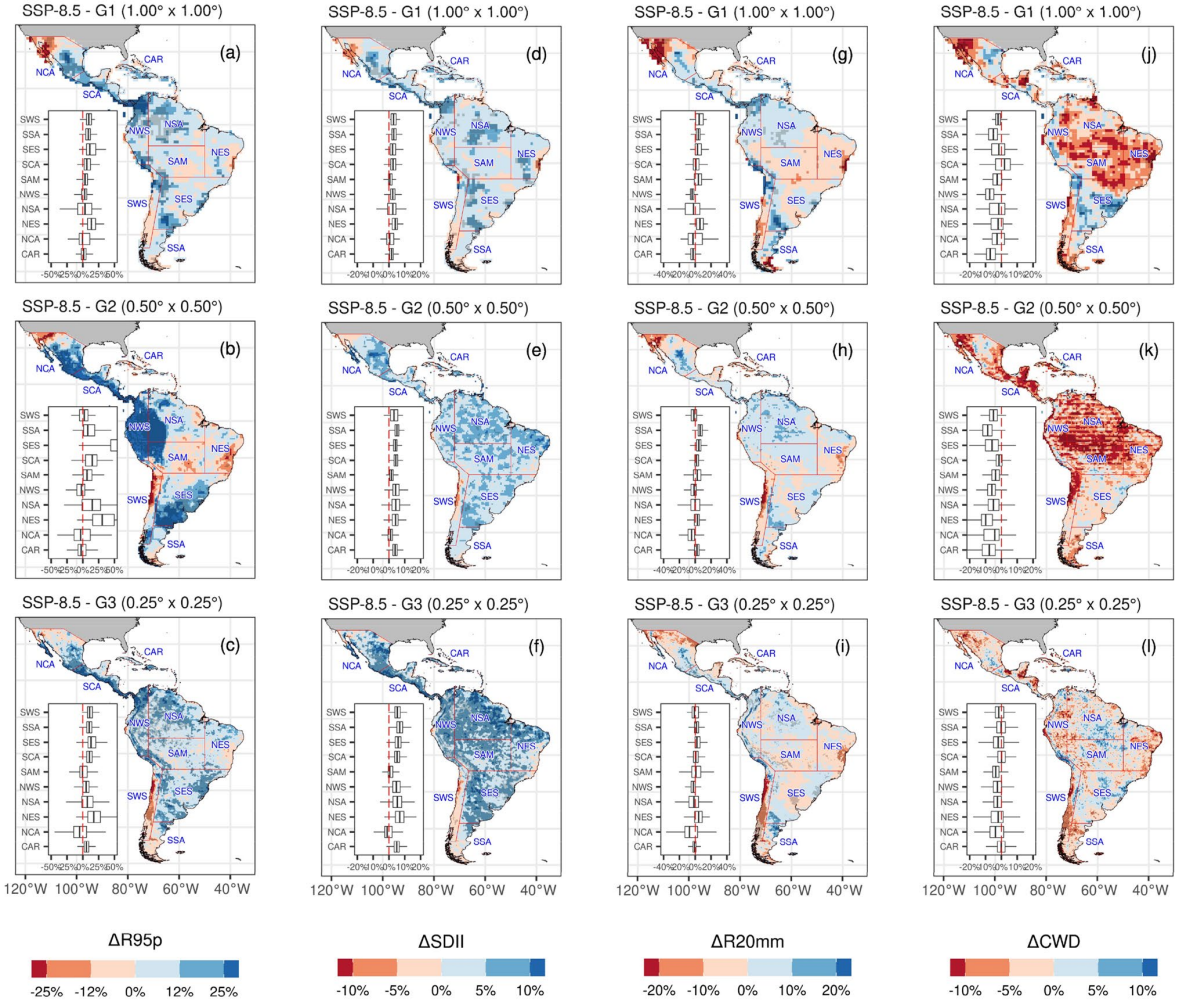
Future changes of multi-model ensemble in precipitation extremes R95p (**a**–**c**), SDII (**d**–**f**), R20mm (**g**–**i**), and CWD (**j**–**l**) indices under SSP5-8.5 scenario for 2021–2050 relative to the reference period (1981–2010)

Projected changes in precipitation indices relative to the 1981–2010 period are presented in Figs. 13 and 14. As depicted by Sillmann et al. (2013) and Avila-Diaz et al. (2020a, b), relative changes are expressed in percentage. Table 4 shows each region's changes for 2021–2050 relative to the reference period 1981–2010.

**Table 4:** Projected changes over the 2021–2050 period in precipitation indices relative to the reference period (1981–2010) for the Multi-Model Ensemble for each resolution group (G_1_L, G_2_I, G_3_H) over analysis regions under the SSP5-8.5 scenario

Group Resolution	Indices	Unit	NCA	SCA	CAR	NWS	NSA	NES	SAM	SWS	SES	SSA
G_1_L 1.0° × 1.0°	PRCPTOT	% of mm	**− 7.14**	2.77	0.74	2.43	0.01	− 6.20	− 4.20	− 4.04	0.33	− 2.48
	RX1day	% of mm	3.25	**10.43**	8.11	6.37	6.20	3.80	2.66	0.71	4.61	1.46
	RX5day	% of mm	2.86	**9.38**	6.67	4.89	4.66	2.71	1.37	0.08	4.56	0.73
	R95p	% of mm	2.22	**13.72**	9.16	13.46	8.44	2.08	1.57	2.95	7.61	3.55
	R20mm	% of days	− 4.11	**5.50**	4.83	4.35	3.75	− 4.36	− 2.46	1.18	1.80	2.46
	SDII	% of mm day^−1^	1.75	**3.82**	3.21	2.70	3.24	2.34	1.65	0.77	2.71	0.79
	CWD	% of days	− 2.39	− 3.14	− 1.59	− 1.96	− 5.30	**− 7.37**	− 6.50	− 1.31	0.88	− 2.52
	CDD	% of days	10.73	0.47	0.81	0.24	9.90	**19.24**	14.15	7.03	10.08	3.83
G_2_I 0.5° × 0.5°	PRCPTOT	% of mm	− 4.73	− 0.73	− 1.45	1.13	0.67	− 4.47	− 1.83	**− 7.70**	0.53	− 2.04
	RX1day	% of mm	4.56	5.89	5.58	7.17	**7.31**	4.43	4.64	− 0.60	5.79	2.36
	RX5day	% of mm	3.34	**5.66**	4.15	4.19	5.13	2.50	3.66	− 1.78	4.77	0.66
	R95p	% of mm	13.77	34.06	1.53	**66.10**	14.66	− 3.34	3.83	− 1.24	14.23	8.07
	R20mm	% of days	− 0.21	2.22	− 1.26	4.19	**6.08**	− 1.92	1.92	− 5.32	2.38	2.38
	SDII	% of mm day^−1^	4.22	4.06	3.26	3.88	**5.07**	4.49	3.87	0.57	4.37	1.78
	CWD	% of days	− 4.81	**− 9.46**	− 5.14	− 6.09	− 8.87	− 6.32	− 7.78	− 5.83	− 1.46	− 3.69
	CDD	% of days	11.61	6.19	3.64	5.57	7.76	13.29	**15.95**	10.39	10.23	5.57
G_3_H 0.25° × 0.25°	PRCPTOT	% of mm	− 3.37	**4.20**	1.26	2.70	0.89	− 2.98	− 1.54	− 7.27	0.35	− 3.20
	RX1day	% of mm	7.11	**10.08**	9.44	8.73	7.88	4.89	5.65	− 1.96	7.03	2.16
	RX5day	% of mm	5.49	**9.32**	7.15	6.55	5.79	3.44	3.83	− 1.90	5.30	0.59
	R95p	% of mm	7.62	**18.10**	11.90	15.22	10.49	4.94	6.00	− 3.69	10.64	1.53
	R20mm	% of days	− 1.57	3.71	0.20	**3.89**	1.65	− 2.99	− 0.90	− 5.06	1.02	1.54
	SDII	% of mm day^−1^	5.48	6.86	5.35	5.82	**6.92**	4.69	4.92	− 0.59	5.20	1.27
	CWD	% of days	− 2.31	− 2.62	− 0.73	− 1.45	− 0.14	− 2.46	0.03	− 2.39	0.19	**− 3.51**
	CDD	% of days	**11.95**	6.20	4.86	7.11	8.61	11.83	9.09	8.62	6.80	5.42

Values in bold represent the highest change for each index

The PRCPTOT index change depicts the highest decrease across resolution and regions over the Chilean coast (SWS), with magnitudes between − 7.7 and − 4.0% for the different resolution groups, respectively. These changes follow the significative negative trends (*p* < 0.05) found for the 1981–2014 period among the three MMEs, though this trend is not statistically significant for the observed datasets. A decrease is also found for NCA and NES: − 7.1% and − 6.2% for the lowest, − 4.7% and − 7.7% for the intermediate, and − 3.4% and − 3.0% for the highest resolution, respectively.

Absolute indices (RX1day and RX5day) show an increase over nearly all regions and between resolutions, with SCA and the CAR depicting the largest increase (more than 8%) for the lowest and highest resolutions. Conversely, SWS reaches − 2.0% for both indices at the intermediate and highest resolution. As for these two indices, SCA region also presents a relative change in the R95p index of 13.7%, 34.1%, and 18.1% from the lowest to the highest resolution, only surpassed by the NWS with 13.5%, 66.1%, and 15.2%, respectively. However, the highest value for both regions could be related to modeling issues, such as the spatial inconsistency in G_2_I-MME. At the same time, the SDII displays a general increase in all regions for the different MMEs, especially in the northern (NCA, SCA, and CAR) and central regions (NWS and NSA), with changes that reach up to 6.92%.

Finally, the spatial variability of CDD is consistent over the three resolutions, with an increase nearly over all regions. In South America, the largest increase is localized in the SAM and NES regions, which agrees with the results by Medeiros et al. (2022) with CMIP6 models. Over SAM, the change reaches nearly 16.0% for the intermediate resolution, while an increase of 19.2% is projected over NES for the same resolution. A similar increase, larger than 10%, is projected for NCA, SWS, and SES. A decrease over Panamá, Colombia, and Venezuela is described by the three MMEs, being this reduction more evident over the lowest resolution. On the other hand, the CWD index is projected to decrease over the Amazon Basin, NCA, and SWS, while it increases over the Andes and La Plata Basin for G_1_L-MME. In the case of the reduction in CWD projected by the intermediate resolution, this is larger than the other two resolutions. A decrease in the magnitude of the projected change in CWD in the Amazon from G1 and G2 resolutions to G3 is remarkable, going from a more pronounced decrease in CWD in G1 and G2 to a pattern close to zero in G3 (Fig. 14j, k, l and Table 4). We could expect that an increase in spatial resolution could improve the simulation of the interaction between surface and atmosphere, and consequently improve the representation of mesoscale meteorological systems, leading to an improvement in the simulation of precipitation. However, this hypothesis cannot be confirmed in our analyses, as the performance of the simulations did not improve with increasing resolution of the climate models (Fig. 8), and agrees with previous findings by Akinsanola et al. (2020) and Bador et al. (2020). Moreover, Almazroui et al. (2021a), analyzing monthly mean temperature and precipitation, found no clear systematic linkage between model performance and the magnitude of projected climate change. In this sense, further studies are needed to investigate this subject in more detail. Additional studies to investigate this in more detail, including an individual ESM assessment to understand how each model projects extreme precipitation climate indices, are encouraged.

**Fig. 15:**
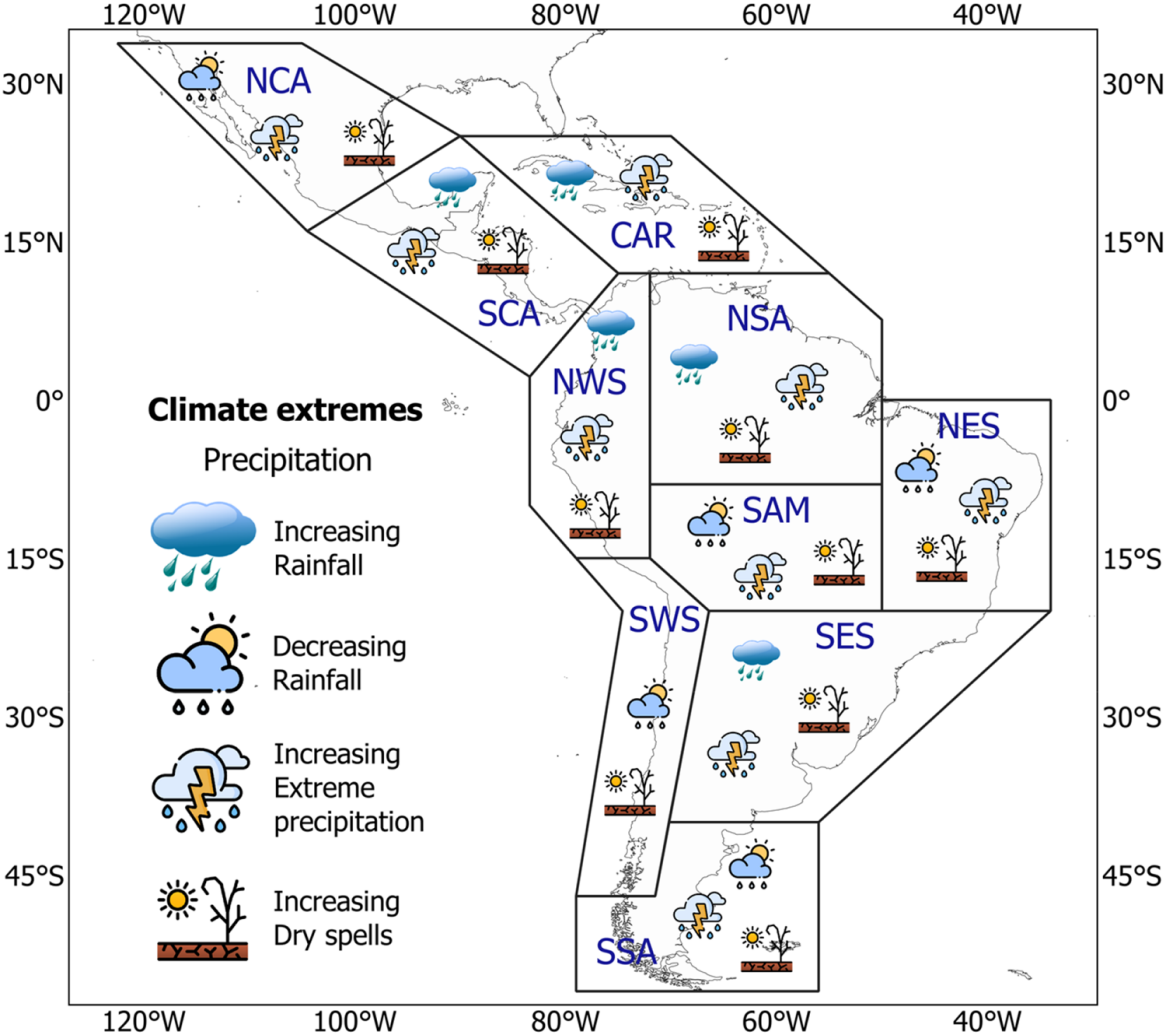
Summary of the projected changes in precipitation climate extremes for each Latin America and Caribbean reference region for the 2021–2050 period under the SSP5-8.5 scenario

In general, results show an increase in all precipitation indices in the future, except for a reduction in PRCPTOT, which is consistent with an increase in CDD. The summary of the main projected changes expected for precipitation climate extremes is depicted in Fig. 15. Studies that have found similar results (Ge et al. 2021; Medeiros and Oliveira 2022; Santos et al. 2019) conclude that this could imply a potential risk of intensified extreme rainfall, which would accentuate the vulnerability of various socioeconomic sectors, such as agriculture, water management, forests and, disasters preparedness.

Over the Amazon Basin, a deficient model representation of land–atmosphere interactions (Baker et al. 2021; Levine et al. 2016; Ruiz-Vásquez et al. 2020; Yin et al. 2013) could affect the estimation of extreme climate indices in ESMs (Avila-Diaz et al. 2020a). Further analysis is needed for the HighResMIP CMIP6 models, particularly for Central America and the Caribbean Islands, in which the land–ocean interaction between the narrow landmass and two oceans (Durán-Quesada et al. 2020; Herrera et al. 2020) is still a challenge for the ESMs.

In summary, the three MMEs describe an intensification of extreme rainfall events, which are spatially consistent between the resolutions (Fig. 15). The greatest changes are mostly located in the SCA region for the lowest and highest resolution. However, there are no strongly projected changes across the different indices in the SSA region. RX1day and RX5day precipitation amounts increase over almost all regions except SWS and the northern part of NCS. This increase is accompanied by a projected rise in the R95p index, which spatially agrees, at least for the G_3_H-MME.

## Discussion and Concluding Remarks

We assessed the performance of a sub-set of HighResMIP models, which are members of the CMIP6, in simulating daily temperature and precipitation climate extremes events over Latin America and the Caribbean region during 1981–2014. This was achieved by comparing three gridded datasets (ERA5, CHIRPS, and GMFD). Additionally, we evaluated the impact of the increase in the horizontal spatial resolution in the HighResMIP models in estimating extreme climate variability on a local/regional scale. Finally, the projected extreme temperature and precipitation changes for 2021–2050 under the Shared Socioeconomic Pathways SSP5-8.5 scenario were investigated.

Historical gridded datasets (reanalysis and satellite-based precipitation product) evaluated during the last few decades (1981–2014) show that, in general, for both temperature and precipitation indices, the ERA5 dataset displayed better results compared to GMFD and CHIRPS, respectively. However, it is worth mentioning that these results depend on the region and models analyzed. For example, in the regional ranking based on the average of the KGE values for the temperature indices, the ERA5 and GMFD datasets presented five models and/or ensembles with a better performance each. Four of these five were the same for both datasets (ECMWF-IFS-LR, G_1_L-MME, G_2_I-MME, and G_3_H-MME). As for the precipitation indices of the five models and/or ensembles with the best performance for ERA5 and CHIRPS, only two were the same for both datasets (ECMWF-IFS-HR and MME-G_1_L).

Regarding the three groups of horizontal resolutions used, we can conclude that there is no strong relationship between an increase in resolution and improved performance of the HighResMIP models, which is consistent with what was found by Bador et al. (2020) and Scoccimarro et al. (2022). Noteworthy, among the five best models for temperature indices, both for ERA5 and GMFD, two are part of the 1.00º × 1.00º resolution (ECMWF-IFS-LR and G_1_L-MME). As for the precipitation indices, three of the five best models compared to the ERA5 dataset are included in the 0.50º × 0.50º resolution (HadGEM3-GC31-MM, ECMWF-IFS-HR, and EC-Earth3P) and two in the 1.00º × 1.00º resolution (CMCC-CM2-HR4 and G_1_L-MME). While, for the CHIRPS dataset, these results are inverted, two at 0.50º × 0.50º resolution (ECMWF-IFS-HR and G_2_I-MME) and three at 1.00º × 1.00º resolution (ECMWF-IFS-LR, CNRM-CM6-1, and G_1_L-MME).

Multi-model ensemble means projections for the near future (2021–2050) indicate an intensification of the warming pattern accompanied by an increase in the extreme precipitation events under the SSP5-8.5 scenario in the three resolutions across most of the ten regions (Figs. 12 and 15). These patterns are strictly in line with the results described by others (AghaKouchak et al. 2020; Gulizia et al. 2022; Medeiros et al. 2022; Olmo et al. 2022). The intensification of temperature warm extreme events may increase heat stress vulnerability (Lapola et al. 2019). Likewise, the projected increase in the extreme precipitation climate indices elevates the risk of heavy rainfall and landslides and indicates that dry spells may be long-lasting in the near future due to climate change (Debortoli et al. 2017; Marengo et al. 2017; Medeiros and Oliveira, 2022). This is consistent with much of what we have experienced over the twentieth and early twenty-first centuries due to a strongly globalized, fossil-fueled society (Chen and Sun 2021; Riahi et al. 2017). However, some spatial inconsistencies exist when comparing the G_2_I-MME with the lowest and highest resolution groups. Therefore, a careful interpretation is needed when analyzing the effect of increasing resolution in future projections. Not all the models from the HighResMIP have climate projection information for all the spatial scales. For example, when evaluating the temperature indices, five models were available for the G_2_I-MME, four for the G_3_H-MME., and only three were used for the G_1_L-MME. For the precipitation indices, we used four, five, and six models for each MME from the lowest to the highest resolution, respectively. Despite this challenge, the spatial representation of percentage changes, at least for the precipitation indices, shows a consistent relationship among the three MME groups. We hypothesize that a misrepresentation of the temperature could be related to the greatest bias of the CNRM-CM6-1-HR described by Almazroui et al. (2021b). We also acknowledge that regions like Central America and the Caribbean require the finest spatial resolution since the width of their landmass and the diversity of phenomena modulate their natural extreme climate variability.

Earth System Models are essential for understanding the climate variability and atmospheric teleconnections that generate possible future scenarios to provide scientifically based decision tools for developing adaptation and mitigation plans. As the latest IPCC report emphasizes (IPCC 2022), these decision-support tools are critical to ensuring a livable future. This is nowhere more evident than in front-line communities across Latin America and the Caribbean, where vulnerability to extreme climate events is high (WMO 2021b). However, ESM models contain inherent uncertainties, and the complexity of phenomena related to extreme meteorological events across the region makes interpreting their outcomes a significant challenge for the climate research community. Furthermore, since local decision makers desire local projection information, continued improvements are needed in the HighResMIP simulations. One such study should utilize bias correction techniques of ESMs with observational reference datasets to provide robust climate projections for climate impact studies that require high horizontal resolution information (e.g., less than 0.25° or 25 km).

## Supplementary Information

Below is the link to the electronic supplementary material.

Supplementary file1 (DOCX 10427 KB)

## Data Availability

The author declares that all the data used in the research are available and without access restrictions.

## References

[CR1] Aerenson T, Tebaldi C, Sanderson B, Lamarque J-F (2018). Changes in a suite of indicators of extreme temperature and precipitation under 1.5 and 2 degrees warming. Environ Res Lett.

[CR2] AghaKouchak A, Chiang F, Huning LS, Love CA, Mallakpour I, Mazdiyasni O, Moftakhari H, Papalexiou SM, Ragno E, Sadegh M (2020). Climate extremes and compound hazards in a warming world. Annu Rev Earth Planet Sci.

[CR3] Aguilar E, Peterson T, Obando P, Frutos R, Retana J, Solera M, Soley J, García IG, Araujo RM, Santos AR, Valle VE, Brunet M, Aguilar L, Álvarez L, Bautista M, Castañón C, Herrera L, Ruano E, Sinay JJ, Sánchez E, Oviedo GIH, Obed F, Salgado JE, Vázquez JL, Baca M, Gutiérrez M, Centella C, Espinosa J, Martínez D, Olmedo B, Espinoza CEO, Núñez R, Haylock M, Benavides H, Mayorga R (2005). Changes in precipitation and temperature extremes in Central America and northern South America, 1961–2003. J Geophys Res.

[CR4] Akinsanola AA, Kooperman GJ, Pendergrass AG, Hannah WM, Reed KA (2020). Seasonal representation of extreme precipitation indices over the United States in CMIP6 present-day simulations. Environ Res Lett.

[CR5] Akinsanola AA, Ongoma V, Kooperman GJ (2021). Evaluation of CMIP6 models in simulating the statistics of extreme precipitation over Eastern Africa. Atmos Res.

[CR6] Almazroui M, Ashfaq M, Islam MN, Rashid IU, Kamil S, Abid MA, O’Brien E, Ismail M, Reboita MS, Sörensson AA, Arias PA, Alves LM, Tippett MK, Saeed S, Haarsma R, Doblas-Reyes FJ, Saeed F, Kucharski F, Nadeem I, Silva-Vidal Y, Rivera JA, Ehsan MA, Martínez-Castro D, Muñoz ÁG, Ali MA, Coppola E, Sylla MB (2021). Assessment of CMIP6 performance and projected temperature and precipitation changes over South America. Earth Syst Environ.

[CR7] Almazroui M, Islam MN, Saeed F, Saeed S, Ismail M, Ehsan MA, Diallo I, O’Brien E, Ashfaq M, Martínez-Castro D, Cavazos T, Cerezo-Mota R, Tippett MK, Gutowski WJ, Alfaro EJ, Hidalgo HG, Vichot-Llano A, Campbell JD, Kamil S, Rashid IU, Sylla MB, Stephenson T, Taylor M, Barlow M (2021). Projected changes in temperature and precipitation over the United States, Central America, and the Caribbean in CMIP6 GCMs. Earth Syst Environ.

[CR8] Almazroui M, Saeed F, Saeed S, Ismail M, Ehsan MA, Islam MN, Abid MA, O’Brien E, Kamil S, Rashid IU, Nadeem I (2021). Projected changes in climate extremes using CMIP6 simulations over SREX regions. Earth Syst Environ.

[CR9] Almeida CT, Oliveira-Júnior JF, Delgado RC, Cubo P, Ramos MC (2017). Spatiotemporal rainfall and temperature trends throughout the Brazilian Legal Amazon, 1973–2013. Int J Climatol.

[CR10] Ambrizzi T, Reboita M, da Rocha R, Llopart M (2019). The state-of-the-art and fundamental aspects of regional climate modeling in South America. Ann NY Acad Sci.

[CR11] Ávila Á, Guerrero F, Escobar Y, Justino F (2019). Recent precipitation trends and floods in the Colombian Andes. Water.

[CR12] Avila-Diaz A, Abrahão G, Justino F, Torres R, Wilson A (2020). Extreme climate indices in Brazil: evaluation of downscaled earth system models at high horizontal resolution. Clim Dyn.

[CR13] Avila-Diaz A, Benezoli V, Justino F, Torres R, Wilson A (2020). Assessing current and future trends of climate extremes across Brazil based on reanalyses and earth system model projections. Clim Dyn.

[CR14] Avila-Diaz A, Bromwich DH, Wilson AB, Justino F, Wang S-H (2021). Climate extremes across the North American Arctic in modern reanalyses. J Clim.

[CR15] Ayehu GT, Tadesse T, Gessesse B, Dinku T (2018). Validation of new satellite rainfall products over the Upper Blue Nile Basin, Ethiopia. Atmos Meas Tech.

[CR16] Bador M, Donat MG, Geoffroy O, Alexander LV (2018). Assessing the robustness of future extreme precipitation intensification in the CMIP5 ensemble. J Clim.

[CR17] Bador M, Boé J, Terray L, Alexander LV, Baker A, Bellucci A, Haarsma R, Koenigk T, Moine M, Lohmann K, Putrasahan DA, Roberts C, Roberts M, Scoccimarro E, Schiemann R, Seddon J, Senan R, Valcke S, Vanniere B (2020). Impact of higher spatial atmospheric resolution on precipitation extremes over land in global climate models. J Geophys Res Atmos.

[CR18] Baker JCA, Garcia-Carreras L, Buermann W, Castilho De Souza D, Marsham JH, Kubota PY, Gloor M, Coelho CAS, Spracklen DV (2021). Robust Amazon precipitation projections in climate models that capture realistic land-atmosphere interactions. Environ Res Lett.

[CR19] Balmaceda-Huarte R, Olmo ME, Bettolli ML, Poggi MM (2021). Evaluation of multiple reanalyses in reproducing the spatio-temporal variability of temperature and precipitation indices over southern South America. Int J Climatol.

[CR20] Ban N, Caillaud C, Coppola E, Pichelli E, Sobolowski S, Adinolfi M, Ahrens B, Alias A, Anders I, Bastin S, Belušić D, Berthou S, Brisson E, Cardoso RM, Chan SC, Christensen OB, Fernández J, Fita L, Frisius T, Gašparac G, Giorgi F, Goergen K, Haugen JE, Hodnebrog Ø, Kartsios S, Katragkou E, Kendon EJ, Keuler K, Lavin-Gullon A, Lenderink G, Leutwyler D, Lorenz T, Maraun D, Mercogliano P, Milovac J, Panitz H-J, Raffa M, Remedio AR, Schär C, Soares PMM, Srnec L, Steensen BM, Stocchi P, Tölle MH, Truhetz H, Vergara-Temprado J, de Vries H, Warrach-Sagi K, Wulfmeyer V, Zander MJ (2021). The first multi-model ensemble of regional climate simulations at kilometer-scale resolution, part I: evaluation of precipitation. Clim Dyn.

[CR21] Baxter S, Bell GD, Blake ES, Bringas FG, Camargo SJ, Chen L, Coelho CAS, Domingues R, Goldenberg SB, Goni G, Fauchereau N, Halpert MS, He Q, Klotzbach PJ, Knaff JA, L’Heureux M, Landsea CW, Lin I-I, Lorrey AM, Luo J-J, Magee AD, Pasch RJ, Pearce PR, Pezza AB, Rosencrans M, Trewin BC, Truchelut RE, Wang B, Wang H, Wood KM, Woolley J-M (2020). State of the climate in 2019. Bull Am Meteorol Soc.

[CR22] Beck H, Wood E, Pan M, Fisher C, Miralles D, van Dijk A, McVicar T, Adler R (2019). MSWEP V2 global 3-hourly 01° precipitation: methodology and quantitative assessment. Bull Am Meteorol Soc.

[CR23] Bozkurt D, Rojas M, Boisier JP, Valdivieso J (2018). Projected hydroclimate changes over Andean basins in central Chile from downscaled CMIP5 models under the low and high emission scenarios. Clim Change.

[CR24] Brown JR, Brierley CM, An S, Guarino M, Stevenson S, Williams CJR, Zhang Q, Zhao A, Abe-Ouchi A, Braconnot P, Brady EC, Chandan D, D’Agostino R, Guo C, LeGrande AN, Lohmann G, Morozova PA, Ohgaito R, O’ishi R, Otto-Bliesner BL, Peltier WR, Shi X, Sime L, Volodin EM, Zhang Z, Zheng W (2020). Comparison of past and future simulations of ENSO in CMIP5/PMIP3 and CMIP6/PMIP4 models. Clim past.

[CR25] Campozano L, Célleri R, Trachte K, Bendix J, Samaniego E (2016). Rainfall and cloud dynamics in the Andes: a southern Ecuador case study. Adv Meteorol.

[CR26] Cavazos T, Luna-Niño R, Cerezo-Mota R, Fuentes-Franco R, Méndez M, Pineda Martínez LF, Valenzuela E (2020). Climatic trends and regional climate models intercomparison over the CORDEX-CAM (Central America, Caribbean, and Mexico) domain. Int J Climatol.

[CR27] Ceron W, Toshie Kayano M, Andreoli RV, Avila A, Canchala T, Francés F, Ayes Rivera I, Alfonso-Morales W, Ferreira de Souza RA, Carvajal-Escobar Y (2020). Streamflow intensification driven by the Atlantic multidecadal oscillation (AMO) in the Atrato river basin, Northwestern Colombia. Water.

[CR28] Cerón WL, Molina-Carpio J, Ayes Rivera I, Andreoli RV, Kayano MT, Canchala T (2020). A principal component analysis approach to assess CHIRPS precipitation dataset for the study of climate variability of the La Plata Basin, Southern South America. Nat Hazards.

[CR29] Cerón WL, Kayano MT, Andreoli RV, Avila-Diaz A, Ayes I, Freitas ED, Martins JA, Souza RAF (2021). Recent intensification of extreme precipitation events in the La Plata Basin in Southern South America (1981–2018). Atmos Res.

[CR30] Chaney N, Sheffield J, Villarini G, Wood E (2014). Development of a high-resolution gridded daily meteorological dataset over sub-Saharan Africa: Spatial analysis of trends in climate extremes. J Clim.

[CR31] Changnon SA, Pielke RA, Changnon D, Sylves RT, Pulwarty R (2000). Human factors explain the increased losses from weather and climate extremes. Bull Am Meteorol Soc.

[CR32] Chen H, Sun J (2021). Anthropogenic influence has increased climate extreme occurrence over China. Sci Bull.

[CR33] Collazo S, Barrucand M, Rusticucci M (2022). Evaluation of CMIP6 models in the representation of observed extreme temperature indices trends in South America. Clim Change.

[CR34] Collins M, Knutti R, Arblaster J, Dufresne J, Fichefet T, Friedlingstein P, Gao X, Gutowski W, Johns T, Krinner G, Shongwe M, Tebaldi C, Weaver A, Wehner M, (2016) Long-term climate change: projections, commitments and irreversibility. In: Intergovernmental Panel on Climate Change (ed) Climate change 2013—the physical science basis. Cambridge University Press, Cambridge, pp 1029–1136. 10.1017/CBO9781107415324.024

[CR35] Condom T, Martínez R, Pabón JD, Costa F, Pineda L, Nieto JJ, López F, Villacis M (2020). Climatological and hydrological observations for the South American Andes: in situ stations, satellite, and reanalysis data sets. Front Earth Sci.

[CR36] Contractor S, Donat MG, Alexander LV, Ziese M, Meyer-Christoffer A, Schneider U, Rustemeier E, Becker A, Durre I, Vose RS (2020). Rainfall Estimates on a Gridded Network (REGEN)—a global land-based gridded dataset of daily precipitation from 1950 to 2016. Hydrol Earth Syst Sci.

[CR37] Cornes R, Jones P (2013). How well does the ERA-Interim reanalysis replicate trends in extremes of surface temperature across Europe?. J Geophys Res Atmos.

[CR38] Croitoru A-E, Piticar A, Burada DC (2016). Changes in precipitation extremes in Romania. Quat Int.

[CR39] da Silva PE, Santos e Silva CM, Spyrides MHC, de Andrade LMB (2019). Precipitation and air temperature extremes in the Amazon and northeast Brazil. Int J Climatol.

[CR40] de Lima J, Alcântara C (2019). Comparison between ERA Interim/ECMWF, CFSR, NCEP/NCAR reanalysis, and observational datasets over the eastern part of the Brazilian Northeast Region. Theor Appl Climatol.

[CR41] de Medeiros FJ, de Oliveira CP, Santos e Silva CM, de Araújo JM (2020). Numerical simulation of the circulation and tropical teleconnection mechanisms of a severe drought event (2012–2016) in Northeastern Brazil. Clim Dyn.

[CR42] de los Skansi MM, Brunet M, Sigró J, Aguilar E, Arevalo G, Bentancur OJ, Castellón G, Correa A, Jácome H, Malheiros R, Oria C, Pasten AM, Sallons S, Villaroel J, Martínez R, Alexander LV, Jones PDD, Arevalo Groening JA, Bentancur OJ, Castellón Geier YR, Correa Amaya RL, Jácome H, Malheiros Ramos A, Oria Rojas C, Pasten AM, Sallons Mitro S, Villaroel Jiménez C, Martínez R, Alexander LV, Jones PDD (2013). Warming and wetting signals emerging from analysis of changes in climate extreme indices over South America. Glob Planet Change.

[CR43] Debortoli NS, Camarinha PIM, Marengo JA, Rodrigues RR (2017). An index of Brazil’s vulnerability to expected increases in natural flash flooding and landslide disasters in the context of climate change. Nat Hazards.

[CR44] Demory ME, Berthou S, Fernández J, Sørland SL, Brogli R, Roberts MJ, Beyerle U, Seddon J, Haarsma R, Schär C, Buonomo E, Christensen OB, Ciarlo JM, Fealy R, Nikulin G, Peano D, Putrasahan D, Roberts CD, Senan R, Steger C, Teichmann C, Vautard R (2020). European daily precipitation according to EURO-CORDEX regional climate models (RCMs) and high-resolution global climate models (GCMs) from the High-Resolution Model Intercomparison Project (HighResMIP). Geosci Model Dev.

[CR45] Denis B, Laprise R, Caya D, Côté J (2002). Downscaling ability of one-way nested regional climate models: the Big-Brother Experiment. Clim Dyn.

[CR46] Depsky N, Pons D (2021). Meteorological droughts are projected to worsen in Central America’s dry corridor throughout the 21st century. Environ Res Lett.

[CR47] Diaconescu EP, Gachon P, Laprise R (2015). On the remapping procedure of daily precipitation statistics and indices used in regional climate model evaluation. J Hydrometeorol.

[CR48] Domínguez-Castro F, Reig F, Vicente-Serrano SM, Aguilar E, Peña-Angulo D, Noguera I, Revuelto J, van der Schrier G, El Kenawy AM (2020). A multidecadal assessment of climate indices over Europe. Sci Data.

[CR49] Donat M, Alexander L, Yang H, Durre I, Vose R, Caesar J (2013). Global land-based datasets for monitoring climatic extremes. Bull Am Meteorol Soc.

[CR50] Donat MG, Alexander LV, Herold N, Dittus AJ (2016). Temperature and precipitation extremes in century-long gridded observations, reanalyses, and atmospheric model simulations. J Geophys Res Atmos.

[CR51] Dosio A, Jones RG, Jack C, Lennard C, Nikulin G, Hewitson B (2019). What can we know about future precipitation in Africa? Robustness, significance and added value of projections from a large ensemble of regional climate models. Clim Dyn.

[CR52] Dunn RJH, Alexander LV, Donat MG, Zhang X, Bador M, Herold N, Lippmann T, Allan R, Aguilar E, Barry AA, Brunet M, Caesar J, Chagnaud G, Cheng V, Cinco T, Durre I, Guzman R, Htay TM, Wan Ibadullah WM, Bin Ibrahim MKI, Khoshkam M, Kruger A, Kubota H, Leng TW, Lim G, Li-Sha L, Marengo J, Mbatha S, McGree S, Menne M, Milagros Skansi M, Ngwenya S, Nkrumah F, Oonariya C, Pabon-Caicedo JD, Panthou G, Pham C, Rahimzadeh F, Ramos A, Salgado E, Salinger J, Sané Y, Sopaheluwakan A, Srivastava A, Sun Y, Timbal B, Trachow N, Trewin B, Schrier G, Vazquez-Aguirre J, Vasquez R, Villarroel C, Vincent L, Vischel T, Vose R, Bin Hj Yussof MN (2020). Development of an updated global land in situ-based data set of temperature and precipitation extremes: HadEX3. J Geophys Res Atmos.

[CR53] Durán-Quesada AM, Sorí R, Ordoñez P, Gimeno L (2020). Climate perspectives in the Intra-Americas seas. Atmosphere (basel).

[CR54] Espinoza JC, Ronchail J, Marengo JA, Segura H (2019). Contrasting North-South changes in Amazon wet-day and dry-day frequency and related atmospheric features (1981–2017). Clim Dyn.

[CR55] Espinoza J-C, Marengo JA, Schongart J, Jimenez JC (2022). The new historical flood of 2021 in the Amazon River compared to major floods of the 21st century: atmospheric features in the context of the intensification of floods. Weather Clim Extrem.

[CR56] Fan X, Miao C, Duan Q, Shen C, Wu Y (2020). The performance of CMIP6 versus CMIP5 in simulating temperature extremes over the global land surface. J Geophys Res Atmos.

[CR57] Faye A, Akinsanola AA (2022). Evaluation of extreme precipitation indices over West Africa in CMIP6 models. Clim Dyn.

[CR58] Funk C, Peterson P, Landsfeld M, Pedreros D, Verdin J, Shukla S, Husak G, Rowland J, Harrison L, Hoell A, Michaelsen J (2015). The climate hazards infrared precipitation with stations—a new environmental record for monitoring extremes. Sci Data.

[CR59] Ge F, Zhu S, Luo H, Zhi X, Wang H (2021). Future changes in precipitation extremes over Southeast Asia: insights from CMIP6 multi-model ensemble. Environ Res Lett.

[CR60] Giorgi F (2005). Climate change prediction. Clim Change.

[CR61] Giorgi F, Francisco R (2001) Uncertainties in the prediction of regional climate change. In: Global change and protected areas. pp 127–139

[CR62] Gouveia CD, Rodrigues Torres R, Marengo JA, Avila-Diaz A (2022). Uncertainties in projections of climate extremes indices in South America via Bayesian inference. Int J Climatol.

[CR63] Gulizia CN, Raggio GA, Camilloni IA, Saurral RI (2022). Changes in mean and extreme climate in southern South America under global warming of 1.5 °C, 2 °C, and 3 °C. Theor Appl Climatol.

[CR64] Gupta HV, Kling H, Yilmaz KK, Martinez GF (2009). Decomposition of the mean squared error and NSE performance criteria: implications for improving hydrological modelling. J Hydrol.

[CR65] Gutjahr O, Putrasahan D, Lohmann K, Jungclaus JH, Von Storch JS, Brüggemann N, Haak H, Stössel A (2019). Max Planck Institute Earth System Model (MPI-ESM1.2) for the High-Resolution Model Intercomparison Project (HighResMIP). Geosci Model Dev.

[CR66] Haarsma RJ, Roberts MJ, Vidale PL, Catherine A, Bellucci A, Bao Q, Chang P, Corti S, Fučkar NS, Guemas V, Von Hardenberg J, Hazeleger W, Kodama C, Koenigk T, Leung LR, Lu J, Luo JJ, Mao J, Mizielinski MS, Mizuta R, Nobre P, Satoh M, Scoccimarro E, Semmler T, Small J, Von Storch JS (2016). High resolution model intercomparison project (HighResMIP v1.0) for CMIP6. Geosci Model Dev.

[CR67] Harris I, Osborn TJ, Jones P, Lister D (2020). Version 4 of the CRU TS monthly high-resolution gridded multivariate climate dataset. Sci Data.

[CR68] Heidinger H, Carvalho L, Jones C, Posadas A, Quiroz R (2018). A new assessment in total and extreme rainfall trends over central and southern Peruvian Andes during 1965–2010. Int J Climatol.

[CR69] Herrera DA, Mendez-Tejeda R, Centella-Artola A, Martínez-Castro D, Ault T, Delanoy R (2020). Projected hydroclimate changes on Hispaniola Island through the 21st Century in CMIP6 Models. Atmosphere (basel).

[CR70] Hersbach H, Bell B, Berrisford P, Hirahara S, Horányi A, Muñoz-Sabater J, Nicolas J, Peubey C, Radu R, Schepers D, Simmons A, Soci C, Abdalla S, Abellan X, Balsamo G, Bechtold P, Biavati G, Bidlot J, Bonavita M, Chiara G, Dahlgren P, Dee D, Diamantakis M, Dragani R, Flemming J, Forbes R, Fuentes M, Geer A, Haimberger L, Healy S, Hogan RJ, Hólm E, Janisková M, Keeley S, Laloyaux P, Lopez P, Lupu C, Radnoti G, Rosnay P, Rozum I, Vamborg F, Villaume S, Thépaut J (2020). The ERA5 global reanalysis. Q J R Meteorol Soc.

[CR71] IPCC (2022) Climate change 2022: mitigation of climate change. In: Contribution of working group III to the sixth assessment report of the intergovernmental panel on climate change, Cambridge. ed. UK and New York. 10.1017/9781009157926

[CR72] Iturbide M, Gutiérrez JM, Alves LM, Bedia J, Cimadevilla E, Cofiño A, Cerezo-Mota R, Di Luca A, Faria SH, Gorodetskaya I, Hauser M, Herrera S, Hewitt H, Hennessy K, Jones R, Krakovska S, Manzanas R, Marínez-Castro D, Narisma GT, Nurhati I, Pinto I, Seneviratne S, van den Hurk B, Vera C (2020). Earth Syst Sci Data Discuss.

[CR73] Jiao D, Xu N, Yang F, Xu K (2021). Evaluation of spatial-temporal variation performance of ERA5 precipitation data in China. Sci Rep.

[CR74] Kim Y-H, Min S-K, Zhang X, Sillmann J, Sandstad M (2020). Evaluation of the CMIP6 multi-model ensemble for climate extreme indices. Weather Clim Extrem.

[CR75] Kitoh A, Endo H (2016). Changes in precipitation extremes projected by a 20-km mesh global atmospheric model. Weather Clim Extrem.

[CR76] Kling H, Fuchs M, Paulin M (2012). Runoff conditions in the upper Danube basin under an ensemble of climate change scenarios. J Hydrol.

[CR77] Kodama C, Ohno T, Seiki T, Yashiro H, Noda AT, Nakano M, Yamada Y, Roh W, Satoh M, Nitta T, Goto D, Miura H, Nasuno T, Miyakawa T, Chen YW, Sugi M (2021). The Nonhydrostatic ICosahedral Atmospheric Model for CMIP6 HighResMIP simulations (NICAM16-S): experimental design, model description, and impacts of model updates. Geosci Model Dev.

[CR78] Lapola DM, Braga DR, Di Giulio GM, Torres RR, Vasconcellos MP (2019). Heat stress vulnerability and risk at the (super) local scale in six Brazilian capitals. Clim Change.

[CR79] Lehmann J, Coumou D, Frieler K (2015). Increased record-breaking precipitation events under global warming. Clim Change.

[CR80] Lehner F, Deser C, Maher N, Marotzke J, Fischer EM, Brunner L, Knutti R, Hawkins E (2020). Partitioning climate projection uncertainty with multiple large ensembles and CMIP5/6. Earth Syst Dyn.

[CR81] Levine PA, Randerson JT, Swenson SC, Lawrence DM (2016). Evaluating the strength of the land–atmosphere moisture feedback in Earth system models using satellite observations. Hydrol Earth Syst Sci.

[CR82] Liang-Liang L, Jian L, Ru-Cong Y (2022). Evaluation of CMIP6 HighResMIP models in simulating precipitation over Central Asia. Adv Clim Chang Res.

[CR83] Libonati R, Geirinhas JL, Silva PS, Russo A, Rodrigues JA, Belém LBC, Nogueira J, Roque FO, DaCamara CC, Nunes AMB, Marengo JA, Trigo RM (2022). Assessing the role of compound drought and heatwave events on unprecedented 2020 wildfires in the Pantanal. Environ Res Lett.

[CR84] Liebmann B, Allured D (2006). Daily precipitation grids for South America. Bull Am Meteorol Soc.

[CR85] Lovino MA, Pierrestegui MJ, Müller OV, Berbery EH, Müller GV, Pasten M (2021). Evaluation of historical CMIP6 model simulations and future projections of temperature and precipitation in Paraguay. Clim Change.

[CR86] Lun Y, Liu L, Cheng L, Li X, Li H, Xu Z (2021). Assessment of GCMs simulation performance for precipitation and temperature from CMIP5 to CMIP6 over the Tibetan Plateau. Int J Climatol.

[CR87] Marengo JA, Tomasella J, Alves LM, Soares WR, Rodriguez DA (2011). The drought of 2010 in the context of historical droughts in the Amazon region. Geophys Res Lett.

[CR91] Marengo JA, Chou SC, Torres RR, Giarolla A, Alves L, Lyra A (2014) Climate change in Central and South America: recent trends, future projections, and impacts on regional agriculture (No. 73). Copenhagen

[CR88] Marengo JA, Torres RR, Alves LM (2017). Drought in Northeast Brazil—past, present, and future. Theor Appl Climatol.

[CR89] Marengo JA, Ambrizzi T, Barreto N, Cunha AP, Ramos AM, Skansi M, Molina Carpio J, Salinas R (2021). The heat wave of October 2020 in central South America. Int J Climatol.

[CR90] Marengo JA, Cunha AP, Cuartas LA, Deusdará Leal KR, Broedel E, Seluchi ME, Michelin CM, De Praga Baião CF, Chuchón Ângulo E, Almeida EK, Kazmierczak ML, Mateus NPA, Silva RC, Bender F (2021). Extreme drought in the Brazilian Pantanal in 2019–2020: characterization, causes, and impacts. Front Water.

[CR92] McPhillips LE, Chang H, Chester MV, Depietri Y, Friedman E, Grimm NB, Kominoski JS, McPhearson T, Méndez-Lázaro P, Rosi EJ, Shafiei Shiva J (2018). Defining extreme events: a cross-disciplinary review. Earth’s Futur.

[CR93] Medeiros FJ, Oliveira CP (2022). Assessment of dry and heavy rainfall days and their projected changes over Northeast Brazil in Coupled Model Intercomparison Project Phase 6 models. Int J Climatol.

[CR94] Medeiros FJ, Oliveira C, Avila-Diaz A (2022). Evaluation of extreme precipitation climate indices and their projected changes for Brazil: From CMIP3 to CMIP6. Weather Clim Extrem.

[CR95] Mistry M (2019). A high-resolution global gridded historical dataset of climate extreme indices. Data.

[CR96] Mysiak J, Torresan S, Bosello F, Mistry M, Amadio M, Marzi S, Furlan E, Sperotto A (2018). Climate risk index for Italy. Phil Trans R Soc A.

[CR97] Na Y, Fu Q, Kodama C (2020). Precipitation probability and its future changes from a global cloud-resolving model and CMIP6 simulations. J Geophys Res Atmos.

[CR98] Nagy GJ, Leal Filho W, Azeiteiro UM, Heimfarth J, Verocai JE, Li C (2018). An assessment of the relationships between extreme weather events, vulnerability, and the impacts on human wellbeing in Latin America. Int J Environ Res Public Health.

[CR99] Nakaegawa T, Kitoh A, Murakami H, Kusunoki S (2014). Annual maximum 5-day rainfall total and maximum number of consecutive dry days over Central America and the Caribbean in the late twenty-first century projected by an atmospheric general circulation model with three different horizontal resolutions. Theor Appl Climatol.

[CR100] Nashwan MS, Shahid S (2019). A novel framework for selecting general circulation models based on the spatial patterns of climate. Int J Climatol.

[CR101] Naumann G, Podestá G, Marengo JA, Luterbacher J, Bavera D, Arias Muñoz C, Barbosa P, Cammalleri C, Chamorro L, Cuartas LA, de Jager A, Escobar C, Hidalgo C, Mazzeschi M, Leal de Moraes OL, McCormick N, Maetens W, Magni D, Masante D, Seluchi ME, de los Milagros Skansi M, Spinoni J, Toreti A (2021) The 2019–2021 extreme drought episode in La Plata Basin. 10.2760/773

[CR102] Ngoma H, Wen W, Ayugi B, Babaousmail H, Karim R, Ongoma V (2021). Evaluation of precipitation simulations in CMIP6 models over Uganda. Int J Climatol.

[CR103] Nogueira SMC, Moreira MM, Lordelo MMV (2018). Evaluating precipitation estimates from Eta, TRMM and CHRIPS data in the South-Southeast Region of Minas Gerais State - Brazil. Remote Sens.

[CR104] O’Neill BC, Tebaldi C, van Vuuren DP, Eyring V, Friedlingstein P, Hurtt G, Knutti R, Kriegler E, Lamarque J-F, Lowe J, Meehl GA, Moss R, Riahi K, Sanderson BM (2016). The scenario model intercomparison project (ScenarioMIP) for CMIP6. Geosci Model Dev.

[CR105] Olmo ME, Bettolli ML (2021). Extreme daily precipitation in southern South America: statistical characterization and circulation types using observational datasets and regional climate models. Clim Dyn.

[CR106] Olmo ME, Weber T, Teichmann C, Bettolli ML (2022). Compound events in South America using the CORDEX-CORE ensemble: current climate conditions and future projections in a global warming scenario. J Geophys Res Atmos.

[CR107] Ongoma V, Chen H, Gao C, Nyongesa AM, Polong F (2018). Future changes in climate extremes over Equatorial East Africa based on CMIP5 multimodel ensemble. Nat Hazards.

[CR108] Ortega G, Arias PA, Villegas JC, Marquet PA, Nobre P (2021). Present-day and future climate over central and South America according to <scp>CMIP5</scp> / <scp>CMIP6</scp> models. Int J Climatol.

[CR109] Pabón-Caicedo JD, Arias PA, Carril AF, Espinoza JC, Borrel LF, Goubanova K, Lavado-Casimiro W, Masiokas M, Solman S, Villalba R (2020). Observed and projected hydroclimate changes in the Andes. Front Earth Sci.

[CR110] Pascale S, Kapnick SB, Delworth TL, Hidalgo HG, Cooke WF (2021). Natural variability vs forced signal in the 2015–2019 Central American drought. Clim Change.

[CR111] Pearson K (1895). VII. Note on regression and inheritance in the case of two parents. Proc R Soc London.

[CR112] Reyer CPO, Adams S, Albrecht T, Baarsch F, Boit A, Canales Trujillo N, Cartsburg M, Coumou D, Eden A, Fernandes E, Langerwisch F, Marcus R, Mengel M, Mira-Salama D, Perette M, Pereznieto P, Rammig A, Reinhardt J, Robinson A, Rocha M, Sakschewski B, Schaeffer M, Schleussner CF, Serdeczny O, Thonicke K (2017). Climate change impacts in Latin America and the Caribbean and their implications for development. Reg Environ Chang.

[CR113] Riahi K, van Vuuren DP, Kriegler E, Edmonds J, O’Neill BC, Fujimori S, Bauer N, Calvin K, Dellink R, Fricko O, Lutz W, Popp A, Cuaresma JC, Samir KC, Leimbach M, Jiang L, Kram T, Rao S, Emmerling J, Ebi K, Hasegawa T, Havlik P, Humpenöder F, Da Silva LA, Smith S, Stehfest E, Bosetti V, Eom J, Gernaat D, Masui T, Rogelj J, Strefler J, Drouet L, Krey V, Luderer G, Harmsen M, Takahashi K, Baumstark L, Doelman JC, Kainuma M, Klimont Z, Marangoni G, Lotze-Campen H, Obersteiner M, Tabeau A, Tavoni M (2017). The shared socioeconomic pathways and their energy, land use, and greenhouse gas emissions implications: an overview. Glob Environ Chang.

[CR114] Rivera JA, Arnould G (2020). Evaluation of the ability of CMIP6 models to simulate precipitation over Southwestern South America: climatic features and long-term trends (1901–2014). Atmos Res.

[CR115] Rivera JA, Marianetti G, Hinrichs S (2018). Validation of CHIRPS precipitation dataset along the Central Andes of Argentina. Atmos Res.

[CR116] Roberts CD, Senan R, Molteni F, Boussetta S, Mayer M, Keeley SPE (2018). Climate model configurations of the ecmwf integrated forecasting system (ecmwf-ifs cycle 43r1) for highresmip. Geosci Model Dev.

[CR117] Roberts MJ, Baker A, Blockley EW, Calvert D, Coward A, Hewitt HT, Jackson LC, Kuhlbrodt T, Mathiot P, Roberts CD, Schiemann R, Seddon J, Vannière B, Luigi Vidale P (2019). Description of the resolution hierarchy of the global coupled HadGEM3-GC3.1 model as used in CMIP6 HighResMIP experiments. Geosci Model Dev.

[CR118] Roberts MJ, Camp J, Seddon J, Vidale PL, Hodges K, Vanniere B, Mecking J, Haarsma R, Bellucci A, Scoccimarro E, Caron LP, Chauvin F, Terray L, Valcke S, Moine MP, Putrasahan D, Roberts C, Senan R, Zarzycki C, Ullrich P (2020). Impact of model resolution on tropical cyclone simulation using the HighResMIP-PRIMAVERA multimodel ensemble. J Clim.

[CR119] Ruiz-Vásquez M, Arias PA, Martínez JA, Espinoza JC (2020). Effects of Amazon basin deforestation on regional atmospheric circulation and water vapor transport towards tropical South America. Clim Dyn.

[CR120] Rusticucci M, Zazulie N (2021). Attribution and projections of temperature extreme trends in South America based on CMIP5 models. Ann N Y Acad Sci.

[CR121] Santos M, Fragoso M, Santos J (2017). Regionalization and susceptibility assessment to daily precipitation extremes in mainland Portugal. Appl Geogr.

[CR122] Santos M, Fonseca A, Fragoso M, Santos JA (2019). Recent and future changes of precipitation extremes in mainland Portugal. Theor Appl Climatol.

[CR123] Schiemann R, Athanasiadis P, Barriopedro D, Doblas-Reyes F, Lohmann K, Roberts MJ, Sein DV, Roberts CD, Terray L, Vidale PL (2020). Northern Hemisphere blocking simulation in current climate models: evaluating progress from the Climate Model Intercomparison Project Phase 5 to 6 and sensitivity to resolution. Weather Clim Dyn.

[CR124] Scoccimarro E, Peano D, Gualdi S, Bellucci A, Lovato T, Fogli PG, Navarra A (2022). Extreme events representation in CMCC-CM2 standard and high-resolution general circulation models. Geosci Model Dev.

[CR126] Seneviratne SI, Hauser M (2020). Regional climate sensitivity of climate extremes in CMIP6 versus CMIP5 multimodel ensembles. Earth’s Futur.

[CR125] Seneviratne SI, Zhang X, Adnan M, Badi W, Dereczynski C, Di Luca A, Ghosh S, Iskandar I, Kossin J, Lewis S, Otto F, Pinto I, Satoh M, Vicente-Serrano SM, Wehner M, Zhou B (2021) Weather and Climate Extreme Events in a Changing Climate. In: Climate Change 2021: The Physical Science Basis. Contribution of Working Group I to the Sixth Assessment Report of the Intergovernmental Panel on Climate Change. In: Masson-Delmotte V, Zhai P, Pirani A, Connors SL, Péan C, Berger S, Caud N, Chen Y, Goldfarb L, Gomis MI, Huang M, Leitzell K, Lonnoy E, Matthews JBR, Maycock TK, Waterfield T, Yelekçi O, Yu R, Zhou B (eds) Climate change 2021: the physical science basis. Contribution of working group I to the sixth assessment report of the intergovernmental panel on climate change. p 366

[CR127] Sheffield J, Goteti G, Wood EF (2006). Development of a 50-year high-resolution global dataset of meteorological forcings for Land surface modeling. J Clim.

[CR128] Shultz JM, Berg RC, Kossin JP, Burkle F, Maggioni A, Pinilla Escobar VA, Castillo MN, Espinel Z, Galea S (2021). Convergence of climate-driven hurricanes and COVID-19: the impact of 2020 hurricanes Eta and Iota on Nicaragua. J Clim Chang Heal.

[CR129] Sillmann J, Kharin VV, Zhang X, Zwiers FW, Bronaugh D (2013). Climate extremes indices in the CMIP5 multimodel ensemble: Part 1. Model evaluation in the present climate. J Geophys Res Atmos.

[CR130] Silveira CDS, Vasconcelos Junior FDC, De Souza Filho FDA, Guimarães SO, Marcos Junior AD, Dos Reis GNL, Porto VC (2019). Performance evaluation of AR5-CMIP5 models for the representation of seasonal and multi-annual variability of precipitation in Brazilian hydropower sector basins under RCP8.5 scenario. Hydrol Sci J.

[CR131] Solman S (2013). Regional climate modeling over South America: a review. Adv Meteorol.

[CR132] Solman SA, Bettolli ML, Doyle ME, Olmo ME, Feijoo M, Martinez D, Blázquez J, Balmaceda Huarte R (2021). Evaluation of multiple downscaling tools for simulating extreme precipitation events over Southeastern South America: a case study approach. Clim Dyn.

[CR133] Srivastava A, Grotjahn R, Ullrich PA (2020). Evaluation of historical CMIP6 model simulations of extreme precipitation over contiguous US regions. Weather Clim Extrem.

[CR134] Stewart IT, Maurer EP, Stahl K, Joseph K (2022). Recent evidence for warmer and drier growing seasons in climate sensitive regions of Central America from multiple global datasets. Int J Climatol.

[CR135] Sun Q, Miao C, Duan Q, Ashouri H, Sorooshian S, Hsu K (2018). A review of global precipitation data sets: data sources, estimation, and intercomparisons. Rev Geophys.

[CR136] Tarek M, Brissette FP, Arsenault R (2020). Evaluation of the ERA5 reanalysis as a potential reference dataset for hydrological modelling over North America. Hydrol Earth Syst Sci.

[CR137] Thibeault JM, Seth A (2014). Changing climate extremes in the Northeast United States: observations and projections from CMIP5. Clim Change.

[CR138] Thorarinsdottir TL, Sillmann J, Haugen M, Gissibl N, Sandstad M (2019). Evaluation of CMIP5 and CMIP6 simulations of historical surface air temperature extremes using proper evaluation methods. Environ Res Lett.

[CR139] Valverde MC, Marengo JA (2014). Extreme Rainfall Indices in the Hydrographic Basins of Brazil. Open J Mod Hydrol.

[CR140] Vannière B, Roberts M, Vidale PL, Hodges K, Demory ME, Caron LP, Scoccimarro E, Terray L, Senan R (2020). The moisture budget of tropical cyclones in HighResMIP models: large-scale environmental balance and sensitivity to horizontal resolution. J Clim.

[CR141] Vichot-Llano A, Martinez-Castro D, Giorgi F, Bezanilla-Morlot A, Centella-Artola A (2021). Comparison of GCM and RCM simulated precipitation and temperature over Central America and the Caribbean. Theor Appl Climatol.

[CR142] Villar JCE, Ronchail J, Guyot JL, Cochonneau G, Naziano F, Lavado W, de Oliveira E, Pombosa R, Vauchel P (2009). Spatio-temporal rainfall variability in the Amazon basin countries (Brazil, Peru, Bolivia, Colombia, and Ecuador). Int J Climatol.

[CR143] Wang K, Clow GD (2020). The diurnal temperature range in CMIP6 models: climatology, variability, and evolution. J Clim.

[CR144] Wehner MF (2020). Characterization of long period return values of extreme daily temperature and precipitation in the CMIP6 models: Part 2, projections of future change. Weather Clim Extrem.

[CR145] Wilson AB, Avila-Diaz A, Oliveira L, Zuluga CF, Mark B (2022). Climate extremes and their impacts on agriculture across the Eastern Corn Belt Region of the U.S.. Weather Clim Extrem.

[CR146] WMO (2022) State of the Climate in Latin America and the Caribbean 2021

[CR147] World Meteorological Organization (WMO) (2021a) State of the Global Climate 2020 (WMO-No. 1264)

[CR148] World Meteorological Organization (WMO) (2021b) State of the Climate in Latin America and the Caribbean (WMO-No. 1272)

[CR149] Yang X, Yu X, Wang Y, He X, Pan M, Zhang M, Liu Y, Ren L, Sheffield J (2020). The optimal multimodel ensemble of bias-corrected CMIP5 climate models over China. J Hydrometeorol.

[CR150] Yin L, Fu R, Shevliakova E, Dickinson RE (2013). How well can CMIP5 simulate precipitation and its controlling processes over tropical South America?. Clim Dyn.

[CR151] You Q, Jiang Z, Wang D, Pepin N, Kang S (2018). Simulation of temperature extremes in the Tibetan Plateau from CMIP5 models and comparison with gridded observations. Clim Dyn.

[CR152] Zelinka MD, Myers TA, McCoy DT, Po-Chedley S, Caldwell PM, Ceppi P, Klein SA, Taylor KE (2020). Causes of higher climate sensitivity in CMIP6 models. Geophys Res Lett.

[CR153] Zilli M, Carvalho L, Liebmann B, Silva Dias M (2017). A comprehensive analysis of trends in extreme precipitation over southeastern coast of Brazil. Int J Climatol.

[CR154] Zuluaga CF, Avila-Diaz A, Justino FB, Wilson AB (2021). Climatology and trends of downward shortwave radiation over Brazil. Atmos Res.

[CR155] Zuluaga CF, Avila-Diaz A, Justino FB, Martins FR, Ceron WL (2022). The climate change perspective of photovoltaic power potential in Brazil. Renew Energy.

