# Supplementary material for: Current and Future Climate Extremes Over Latin America and Caribbean: Assessing Earth System Models from High Resolution Model Intercomparison Project (HighResMIP)
Source: Earth Syst Environ. 2022 Dec 19;7(1):99–130. doi: 10.1007/s41748-022-00337-7 (PMC9762667; doi:10.1007/s41748-022-00337-7)
Supplement: Supplementary file 1 — Supplementary file1 (DOCX 10427 KB) [file 41748_2022_337_MOESM1_ESM.docx]

**SUPPLEMENTARY MATERIAL**

**CURRENT AND FUTURE CLIMATE EXTREMES OVER LATIN AMERICA AND CARIBBEAN: ASSESSING EARTH SYSTEM MODELS FROM HIGH RESOLUTION MODEL INTERCOMPARISON PROJECT (HighResMIP)**

Alvaro Avila-Diaz^1,2^, Roger Rodrigues Torres^1^, Cristian Felipe Zuluaga^3^, Wilmar L. Cerón^4^, Lais Oliveira^3^, Victor Benezoli^3^, Irma Ayes Rivera^5^, Jose Antonio Marengo^6^, Aaron B. Wilson^7,8^ Felipe Medeiros^9^

^1^Natural Resources Institute, Universidade Federal de Itajubá, Itajubá, MG, Brazil

^2^Universidad de Ciencias Aplicadas y Ambientales - UDCA, Bogota, Colombia

^3^Department of Agricultural Engineering, Universidade Federal de Viçosa, Viçosa, MG, Brazil

^4^Department of Geography, Faculty of Humanities, Universidad del Valle, Cali 760032, Colombia

^5^International Center for Tropical Agriculture (CIAT), Tegucigalpa, Honduras

^6^National Center for Monitoring and Early Warning of Natural Disasters - CEMADEN, São Jose dos Campos, Brazil.

^7^Byrd Polar and Climate Research Center, The Ohio State University, Columbus, Ohio, U.S.A.

^8^Department of Extension, College of Food, Agricultural, and Environmental Sciences, The Ohio State University, Columbus, Ohio, U.S.A

^9^Graduate Program in Climate Sciences, Federal University of Rio Grande do Norte, Natal, RN, Brazil

Correspondence to Alvaro Avila-Diaz (+57 3155779561): [alvaroaviladiaz@outlook.com](mailto:alvaroaviladiaz@outlook.com)

[**Fig. S1.** KGE components values in the performance evaluation of temperature indices of CMIP6 models compared to ERA5 and CHIRPS over the 1981–2014 period. a) Correlation coefficient (CORR; The asterisks indicate significant correlation at the 95% level), b) bias ratio (BR), and c) relative variability (RV). The number of regions displayed in Fig. 1. 3](#_Toc93919469)

[**Fig. S2.** KGE components values in the performance evaluation of precipitation indices of CMIP6 models compared to ERA5 and CHIRPS over the 1981–2014 period. a) Correlation coefficient (CORR; The asterisks indicate significant correlation at the 95% level), b) bias ratio (BR), and c) relative variability (RV). The number of regions displayed in Fig. 1. 4](#_Toc93919470)

[**Fig. S3.** The KGE (a-f) and climatology bias (g-l) of multi-model ensemble (MME) for the TNn during 1981-2014 compared between the ERA5 (left side) and GMFD (right side). The G_1_L-MME, G_2_I-MME, G_3_H-MME are the groups based on size of grid (sg) of the MME: low (≥0.8° sg ≤1.87°), intermediate (≥0.5° sg ≤0.7°), and high resolution (≥0.23° sg ≤0.35°), respectively. 5](#_Toc93919471)

[**Fig. S4.** The KGE (a-f) and climatology bias (g-l) of multi-model ensemble (MME) for the DTR during 1981-2014 compared between the ERA5 (left side) and GMFD (right side). The G_1_L-MME, G_2_I-MME, G_3_H-MME are the groups based on size of grid (sg) of the MME: low (≥0.8° sg ≤1.87°), intermediate (≥0.5° sg ≤0.7°), and high resolution (≥0.23° sg ≤0.35°), respectively. 6](#_Toc93919472)

[**Fig. S5.** The KGE (a-f) and climatology bias (g-l) of multi-model ensemble (MME) for the TN10p during 1981-2014 compared between the ERA5 (left side) and GMFD (right side). The G_1_L-MME, G_2_I-MME, G_3_H-MME are the groups based on size of grid (sg) of the MME: low (≥0.8° sg ≤1.87°), intermediate (≥0.5° sg ≤0.7°), and high resolution (≥0.23° sg ≤0.35°), respectively. 7](#_Toc93919473)

[**Fig. S6.** The KGE (a-f) and climatology bias (g-l) of multi-model ensemble (MME) for the TN90p during 1981-2014 compared between the ERA5 (left side) and GMFD (right side). The G_1_L-MME, G_2_I-MME, G_3_H-MME are the groups based on size of grid (sg) of the MME: low (≥0.8° sg ≤1.87°), intermediate (≥0.5° sg ≤0.7°), and high resolution (≥0.23° sg ≤0.35°), respectively. 8](#_Toc93919474)

[**Fig. S7.** The KGE (a-f) and climatology bias (g-l) of multi-model ensemble (MME) for the TX10p during 1981-2014 compared between the ERA5 (left side) and GMFD (right side). The G_1_L-MME, G_2_I-MME, G_3_H-MME are the groups based on size of grid (sg) of the MME: low (≥0.8° sg ≤1.87°), intermediate (≥0.5° sg ≤0.7°), and high resolution (≥0.23° sg ≤0.35°), respectively. 9](#_Toc93919475)

[**Fig. S8.** The KGE (a-f) and climatology bias (g-l) of multi-model ensemble (MME) for the TX90p during 1981-2014 compared between the ERA5 (left side) and GMFD (right side). The G_1_L-MME, G_2_I-MME, G_3_H-MME are the groups based on size of grid (sg) of the MME: low (≥0.8° sg ≤1.87°), intermediate (≥0.5° sg ≤0.7°), and high resolution (≥0.23° sg ≤0.35°), respectively. 10](#_Toc93919476)

[**Fig. S9.** The KGE (a-f) and climatology bias (g-l) of multi-model ensemble (MME) for the RX1day during 1981-2014 compared between the ERA5 (left side) and GMFD (right side). The G_1_L-MME, G_2_I-MME, G_3_H-MME are the groups based on size of grid (sg) of the MME: low (≥0.8° sg ≤1.87°), intermediate (≥0.5° sg ≤0.7°), and high resolution (≥0.23° sg ≤0.35°), respectively. 11](#_Toc93919477)

[**Fig. S10.** The KGE (a-f) and climatology bias (g-l) of multi-model ensemble (MME) for the RX5day during 1981-2014 compared between the ERA5 (left side) and GMFD (right side). The G_1_L-MME, G_2_I-MME, G_3_H-MME are the groups based on size of grid (sg) of the MME: low (≥0.8° sg ≤1.87°), intermediate (≥0.5° sg ≤0.7°), and high resolution (≥0.23° sg ≤0.35°), respectively. 12](#_Toc93919478)

[**Fig. S11.** The KGE (a-f) and climatology bias (g-l) of multi-model ensemble (MME) for the R95p during 1981-2014 compared between the ERA5 (left side) and GMFD (right side). The G_1_L-MME, G_2_I-MME, G_3_H-MME are the groups based on size of grid (sg) of the MME: low (≥0.8° sg ≤1.87°), intermediate (≥0.5° sg ≤0.7°), and high resolution (≥0.23° sg ≤0.35°), respectively. 13](#_Toc93919479)

[**Fig. S12.** The KGE (a-f) and climatology bias (g-l) of multi-model ensemble (MME) for the SDII during 1981-2014 compared between the ERA5 (left side) and GMFD (right side). The G_1_L-MME, G_2_I-MME, G_3_H-MME are the groups based on size of grid (sg) of the MME: low (≥0.8° sg ≤1.87°), intermediate (≥0.5° sg ≤0.7°), and high resolution (≥0.23° sg ≤0.35°), respectively. 14](#_Toc93919480)

[**Fig. S13.** The KGE (a-f) and climatology bias (g-l) of multi-model ensemble (MME) for the CWD during 1981-2014 compared between the ERA5 (left side) and GMFD (right side). The G_1_L-MME, G_2_I-MME, G_3_H-MME are the groups based on size of grid (sg) of the MME: low (≥0.8° sg ≤1.87°), intermediate (≥0.5° sg ≤0.7°), and high resolution (≥0.23° sg ≤0.35°), respectively. 15](#_Toc93919481)

[**Fig. S14.** The KGE (a-f) and climatology bias (g-l) of multi-model ensemble (MME) for the CDD during 1981-2014 compared between the ERA5 (left side) and GMFD (right side). The G_1_L-MME, G_2_I-MME, G_3_H-MME are the groups based on size of grid (sg) of the MME: low (≥0.8° sg ≤1.87°), intermediate (≥0.5° sg ≤0.7°), and high resolution (≥0.23° sg ≤0.35°), respectively. 16](#_Toc93919482)


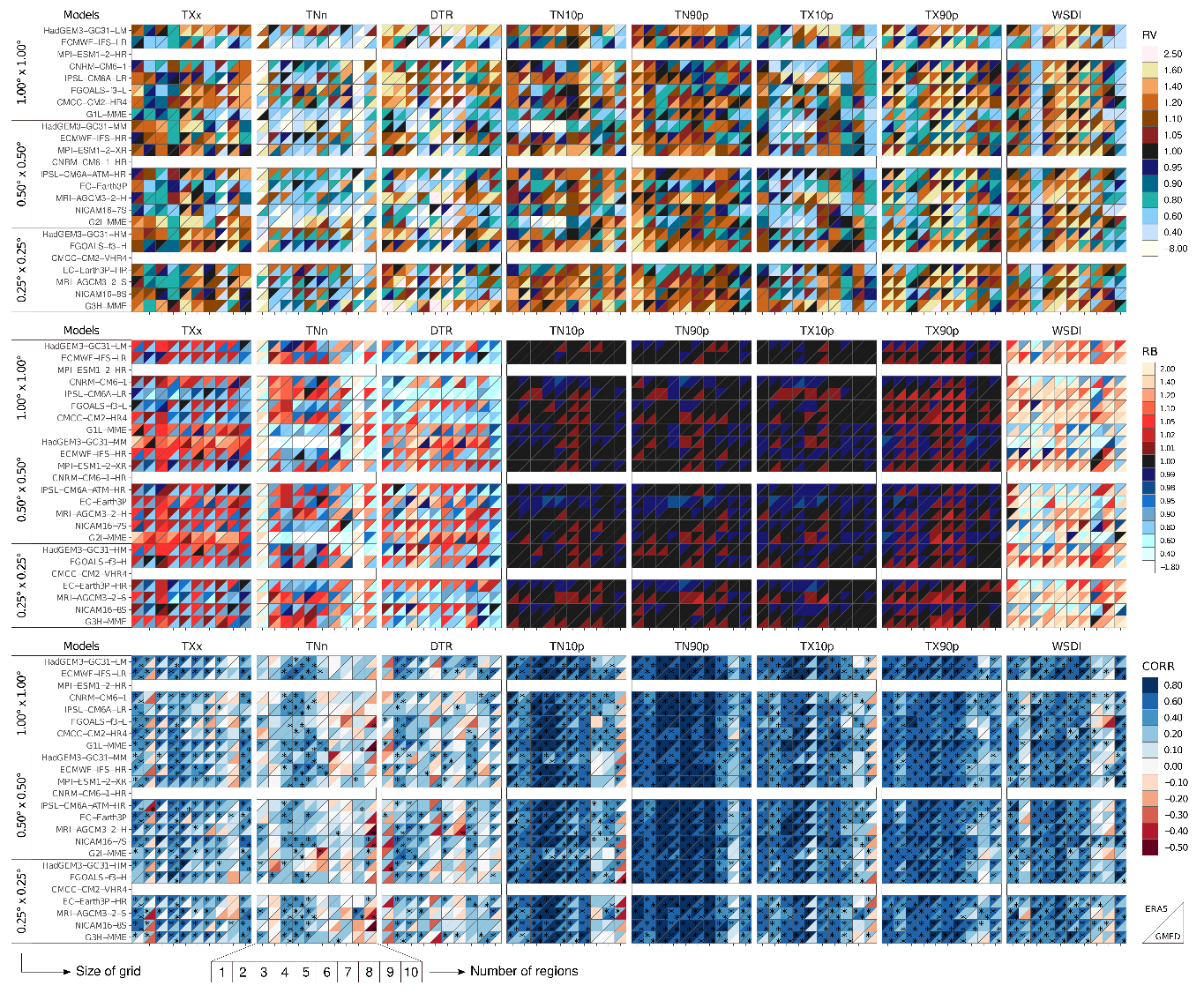


Fig. S1. KGE components values in the performance evaluation of temperature indices of CMIP6 models compared to ERA5 and CHIRPS over the 1981–2014 period. a) Correlation coefficient (CORR; The asterisks indicate significant correlation at the 95% level), b) bias ratio (BR), and c) relative variability (RV). The number of regions displayed in Fig. 1.


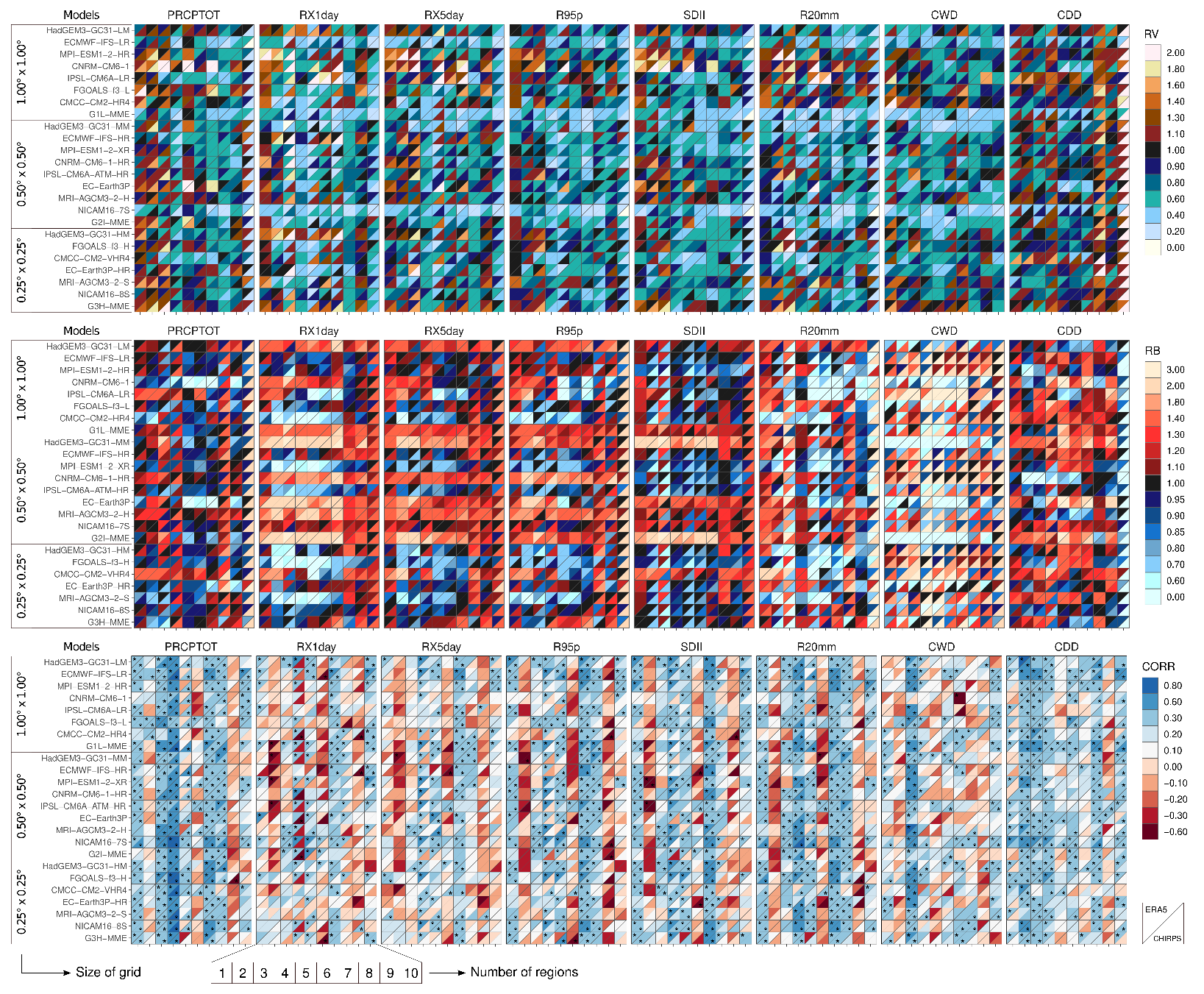


Fig. S2. KGE components values in the performance evaluation of precipitation indices of CMIP6 models compared to ERA5 and CHIRPS over the 1981–2014 period. a) Correlation coefficient (CORR; The asterisks indicate significant correlation at the 95% level), b) bias ratio (BR), and c) relative variability (RV). The number of regions displayed in Fig. 1.

#
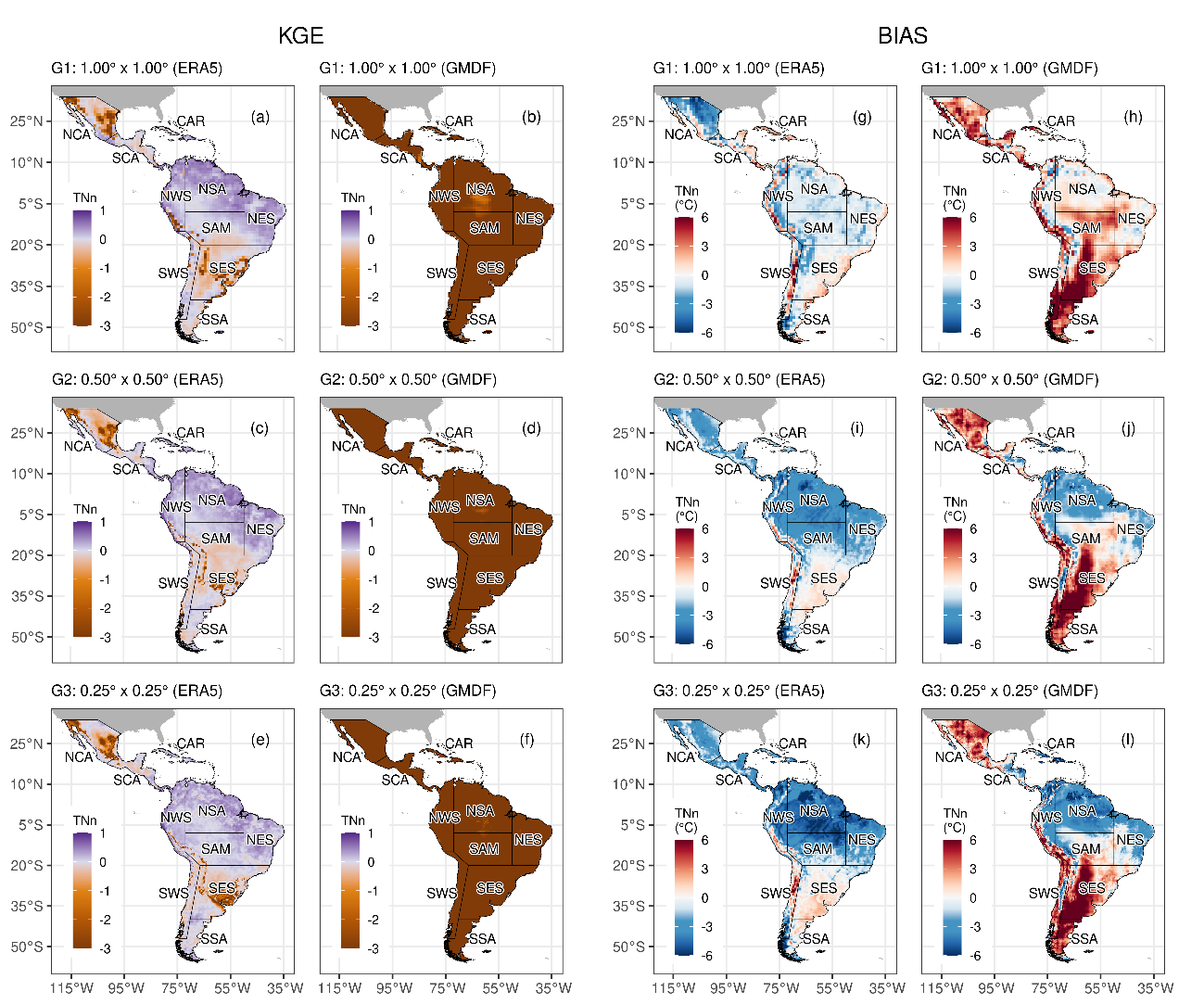


Fig. S3 The KGE (a-f) and climatology bias (g-l) of multi-model ensemble (MME) for the TNn during 1981-2014 compared between the ERA5 (left side) and GMFD (right side). The G_1_L-MME, G_2_I-MME, G_3_H-MME are the groups based on size of grid (sg) of the MME: low (≥0.8° sg ≤1.87°), intermediate (≥0.5° sg ≤0.7°), and high resolution (≥0.23° sg ≤0.35°), respectively.


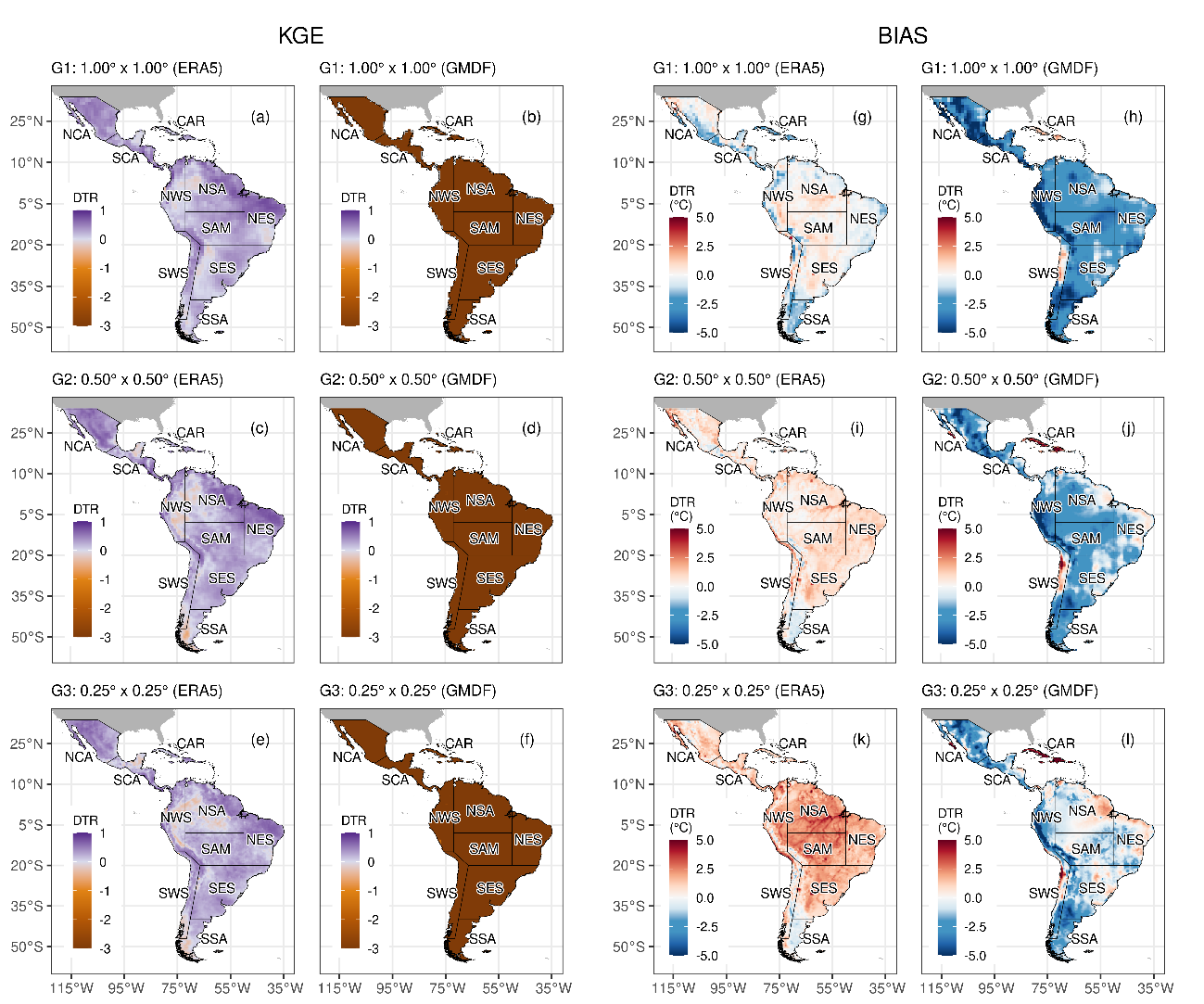


Fig. S4 The KGE (a-f) and climatology bias (g-l) of multi-model ensemble (MME) for the DTR during 1981-2014 compared between the ERA5 (left side) and GMFD (right side). The G_1_L-MME, G_2_I-MME, G_3_H-MME are the groups based on size of grid (sg) of the MME: low (≥0.8° sg ≤1.87°), intermediate (≥0.5° sg ≤0.7°), and high resolution (≥0.23° sg ≤0.35°), respectively.


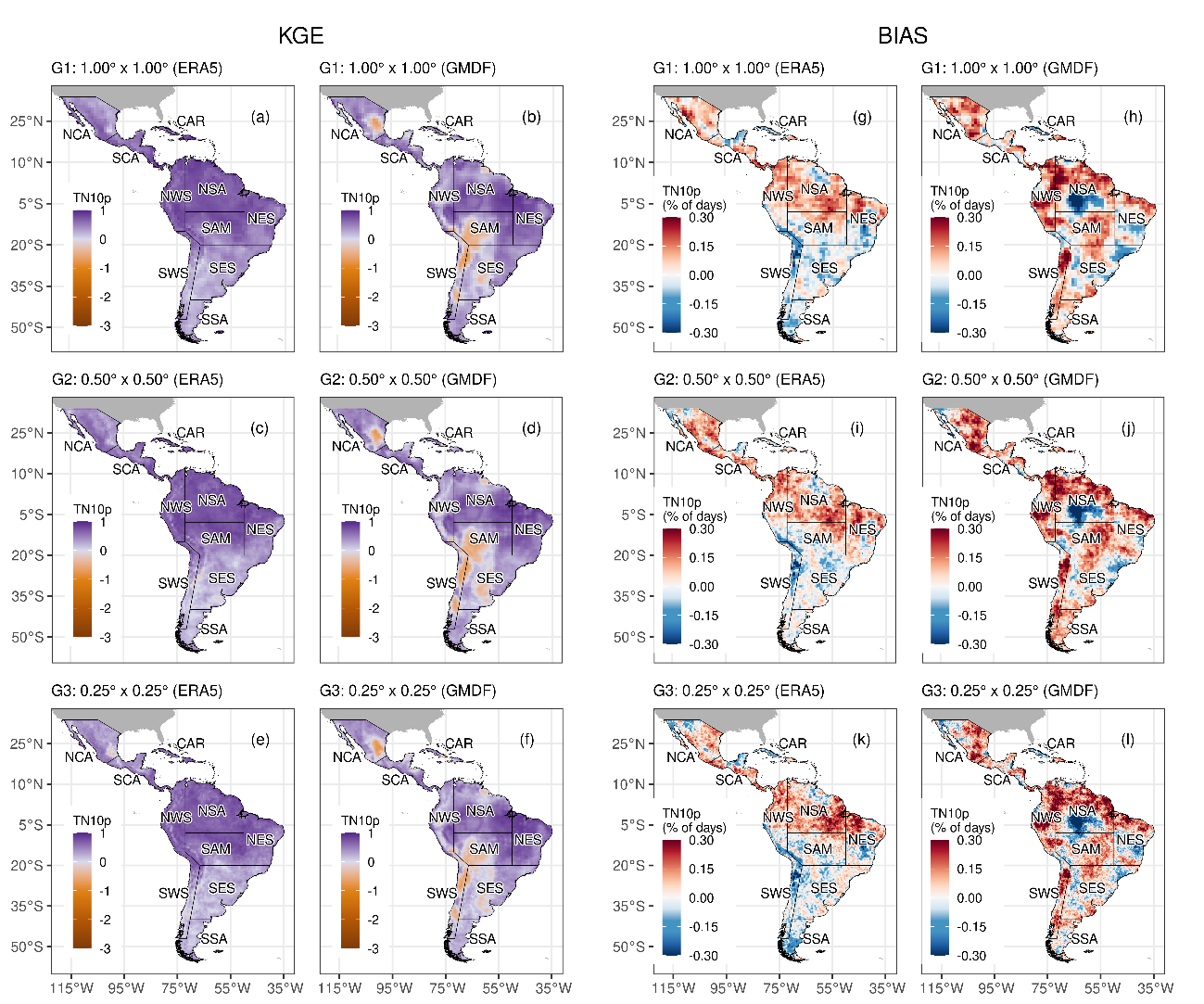


Fig. S5 The KGE (a-f) and climatology bias (g-l) of multi-model ensemble (MME) for the TN10p during 1981-2014 compared between the ERA5 (left side) and GMFD (right side). The G_1_L-MME, G_2_I-MME, G_3_H-MME are the groups based on size of grid (sg) of the MME: low (≥0.8° sg ≤1.87°), intermediate (≥0.5° sg ≤0.7°), and high resolution (≥0.23° sg ≤0.35°), respectively.


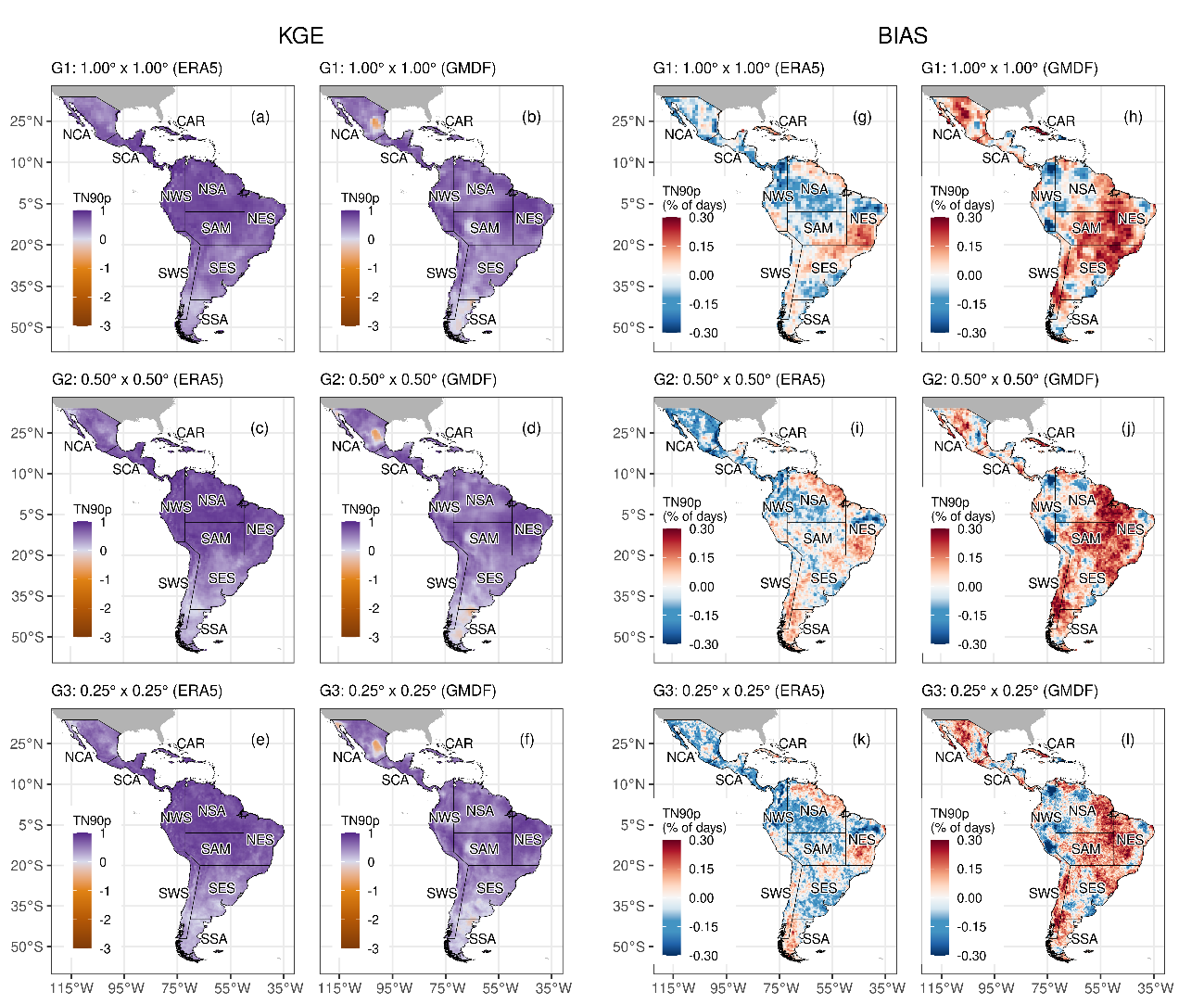


Fig. S6 The KGE (a-f) and climatology bias (g-l) of multi-model ensemble (MME) for the TN90p during 1981-2014 compared between the ERA5 (left side) and GMFD (right side). The G_1_L-MME, G_2_I-MME, G_3_H-MME are the groups based on size of grid (sg) of the MME: low (≥0.8° sg ≤1.87°), intermediate (≥0.5° sg ≤0.7°), and high resolution (≥0.23° sg ≤0.35°), respectively.


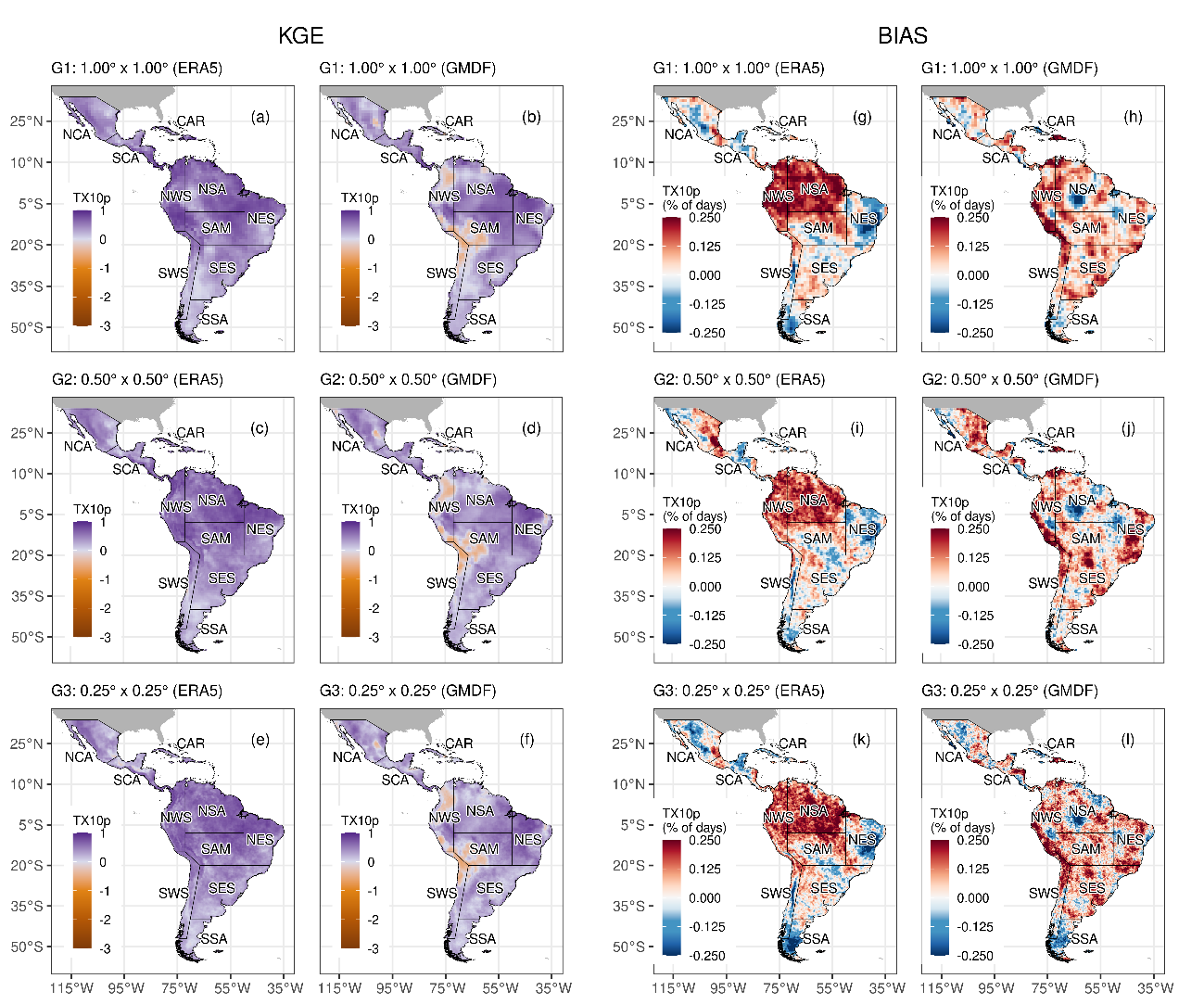


Fig. S7 The KGE (a-f) and climatology bias (g-l) of multi-model ensemble (MME) for the TX10p during 1981-2014 compared between the ERA5 (left side) and GMFD (right side). The G_1_L-MME, G_2_I-MME, G_3_H-MME are the groups based on size of grid (sg) of the MME: low (≥0.8° sg ≤1.87°), intermediate (≥0.5° sg ≤0.7°), and high resolution (≥0.23° sg ≤0.35°), respectively.


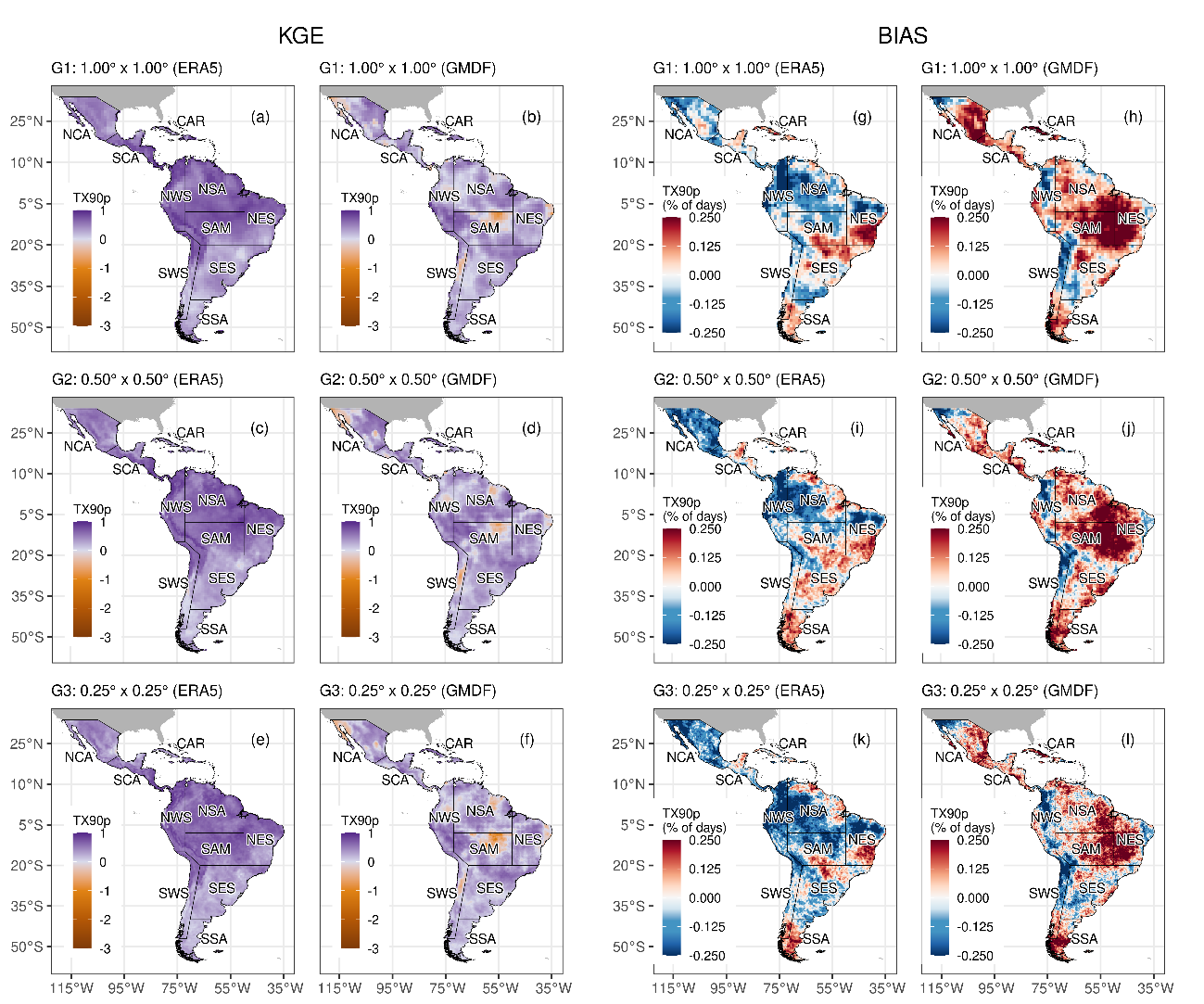


Fig. S8 The KGE (a-f) and climatology bias (g-l) of multi-model ensemble (MME) for the TX90p during 1981-2014 compared between the ERA5 (left side) and GMFD (right side). The G_1_L-MME, G_2_I-MME, G_3_H-MME are the groups based on size of grid (sg) of the MME: low (≥0.8° sg ≤1.87°), intermediate (≥0.5° sg ≤0.7°), and high resolution (≥0.23° sg ≤0.35°), respectively.


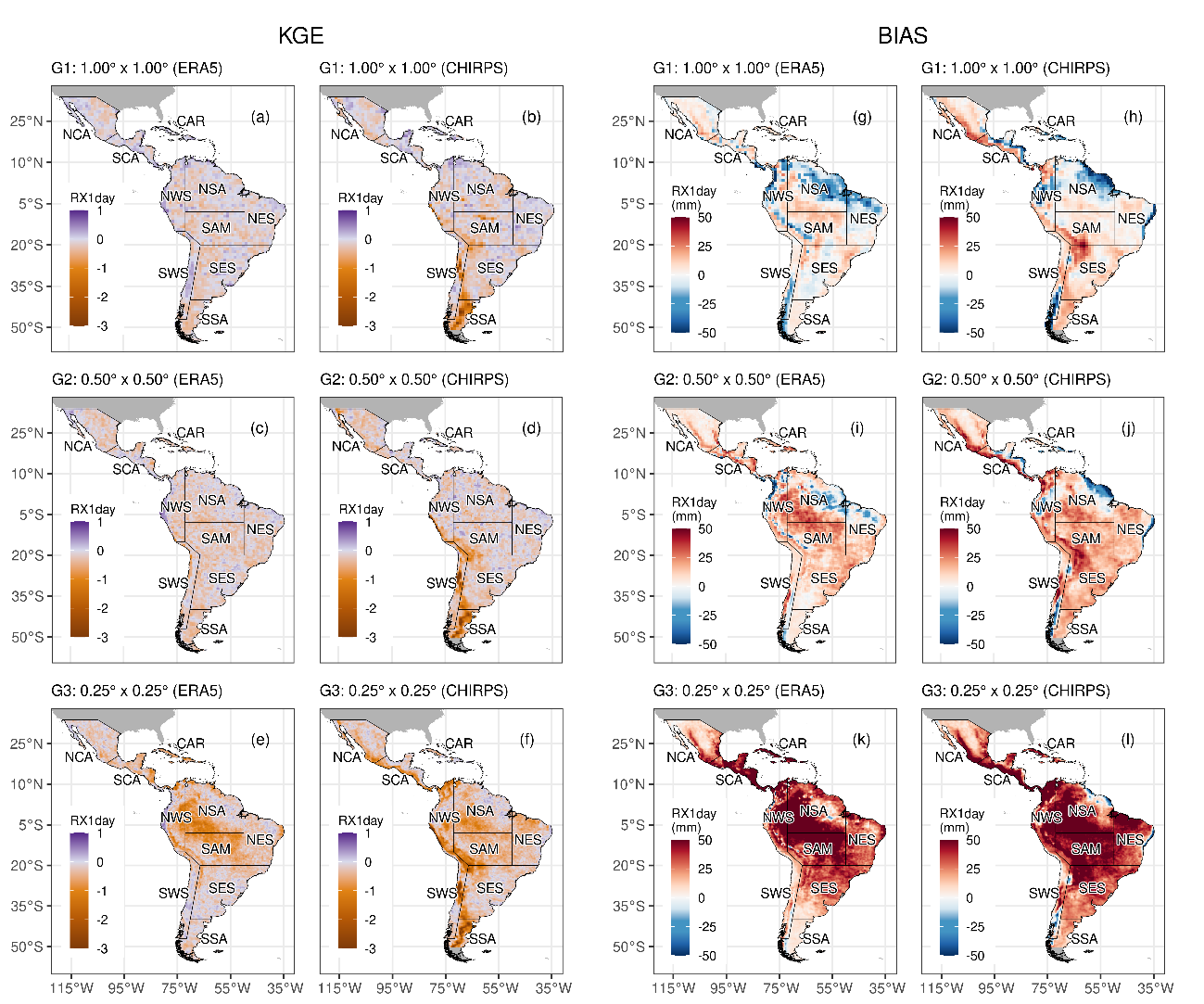


Fig. S9 The KGE (a-f) and climatology bias (g-l) of multi-model ensemble (MME) for the RX1day during 1981-2014 compared between the ERA5 (left side) and GMFD (right side). The G_1_L-MME, G_2_I-MME, G_3_H-MME are the groups based on size of grid (sg) of the MME: low (≥0.8° sg ≤1.87°), intermediate (≥0.5° sg ≤0.7°), and high resolution (≥0.23° sg ≤0.35°), respectively.


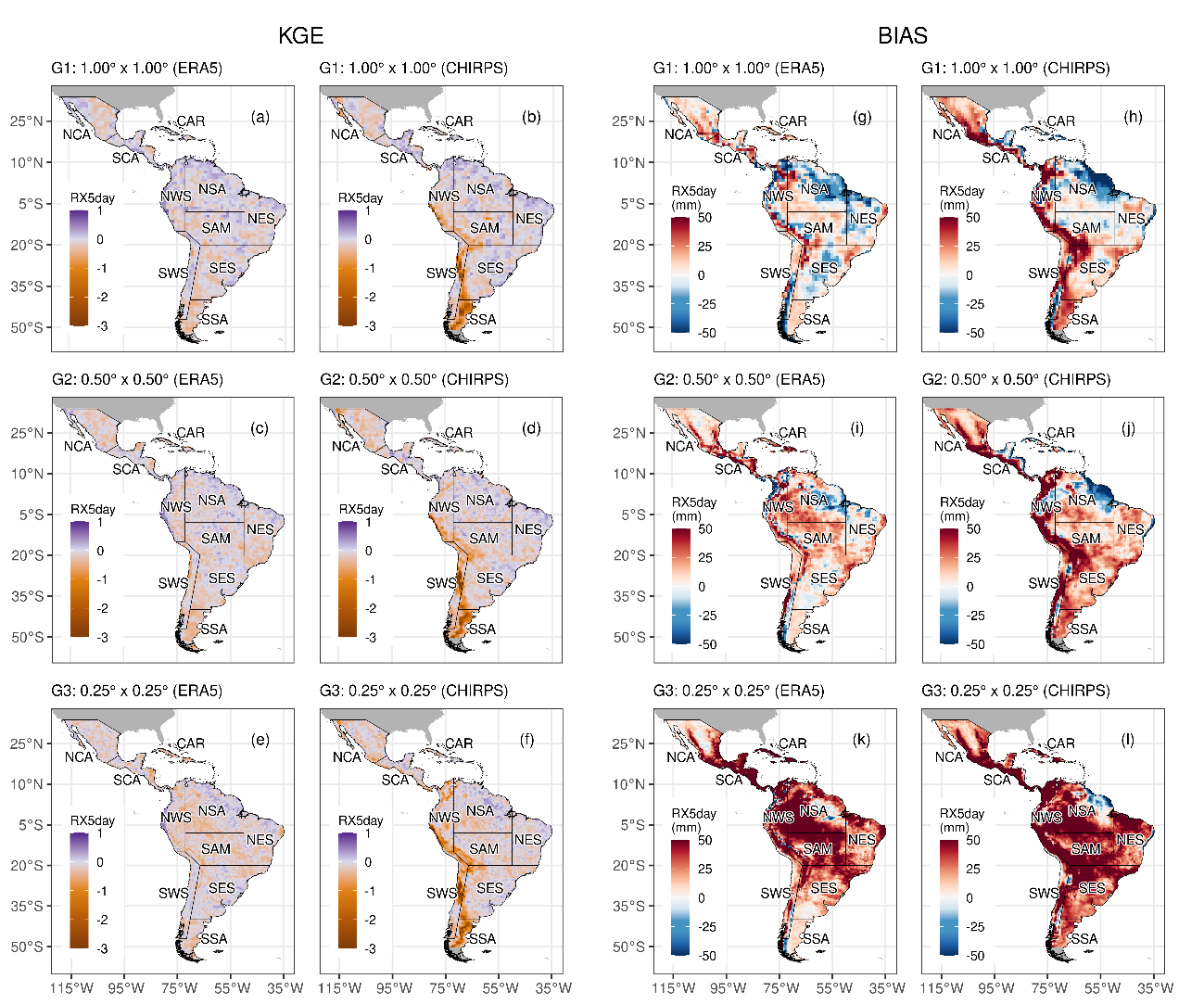


Fig. S10 The KGE (a-f) and climatology bias (g-l) of multi-model ensemble (MME) for the RX5day during 1981-2014 compared between the ERA5 (left side) and GMFD (right side). The G_1_L-MME, G_2_I-MME, G_3_H-MME are the groups based on size of grid (sg) of the MME: low (≥0.8° sg ≤1.87°), intermediate (≥0.5° sg ≤0.7°), and high resolution (≥0.23° sg ≤0.35°), respectively.


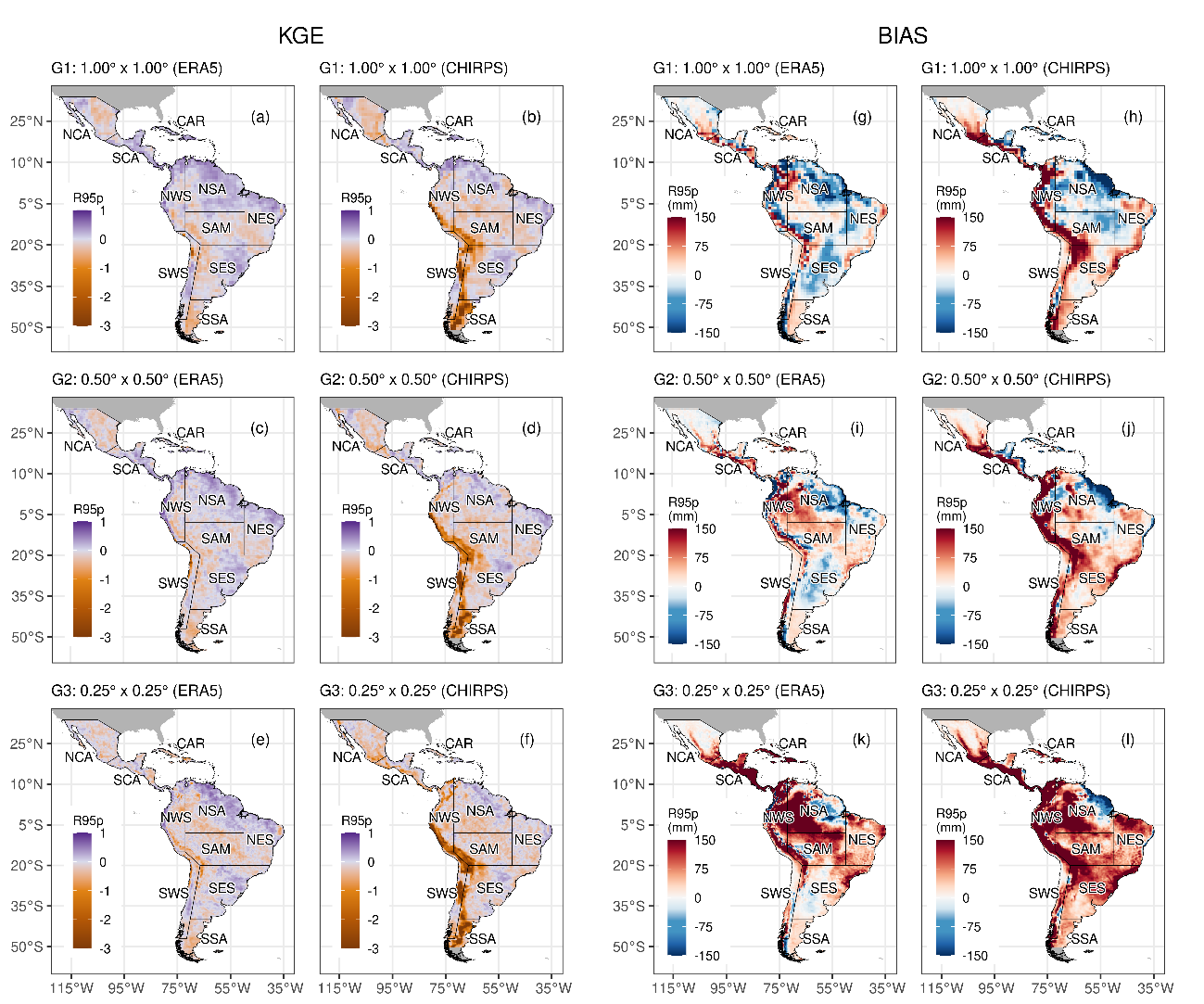


Fig. S11 The KGE (a-f) and climatology bias (g-l) of multi-model ensemble (MME) for the R95p during 1981-2014 compared between the ERA5 (left side) and GMFD (right side). The G_1_L-MME, G_2_I-MME, G_3_H-MME are the groups based on size of grid (sg) of the MME: low (≥0.8° sg ≤1.87°), intermediate (≥0.5° sg ≤0.7°), and high resolution (≥0.23° sg ≤0.35°), respectively.


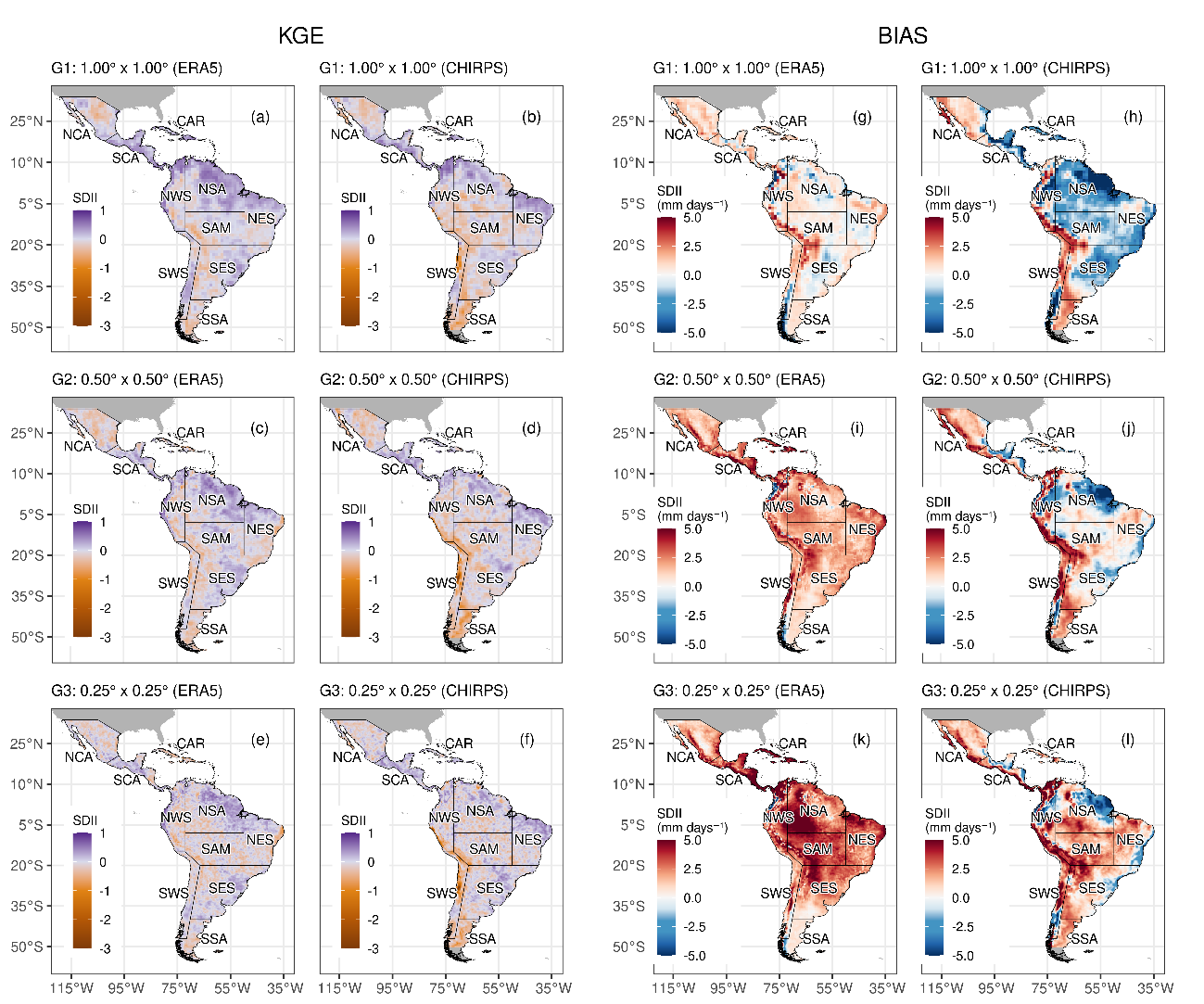


Fig. S12 The KGE (a-f) and climatology bias (g-l) of multi-model ensemble (MME) for the SDII during 1981-2014 compared between the ERA5 (left side) and GMFD (right side). The G_1_L-MME, G_2_I-MME, G_3_H-MME are the groups based on size of grid (sg) of the MME: low (≥0.8° sg ≤1.87°), intermediate (≥0.5° sg ≤0.7°), and high resolution (≥0.23° sg ≤0.35°), respectively.


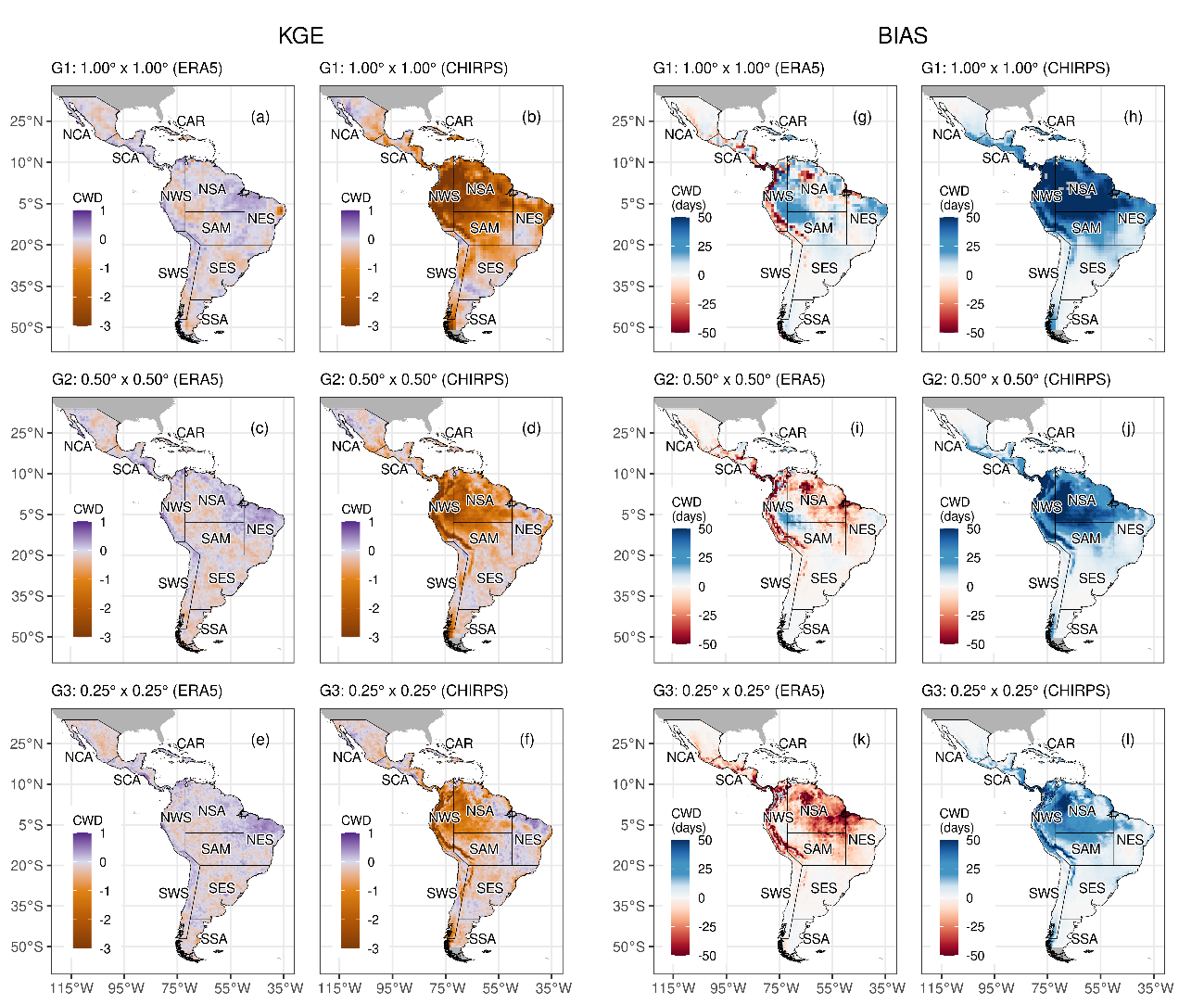


Fig. S13 The KGE (a-f) and climatology bias (g-l) of multi-model ensemble (MME) for the CWD during 1981-2014 compared between the ERA5 (left side) and GMFD (right side). The G_1_L-MME, G_2_I-MME, G_3_H-MME are the groups based on size of grid (sg) of the MME: low (≥0.8° sg ≤1.87°), intermediate (≥0.5° sg ≤0.7°), and high resolution (≥0.23° sg ≤0.35°), respectively.


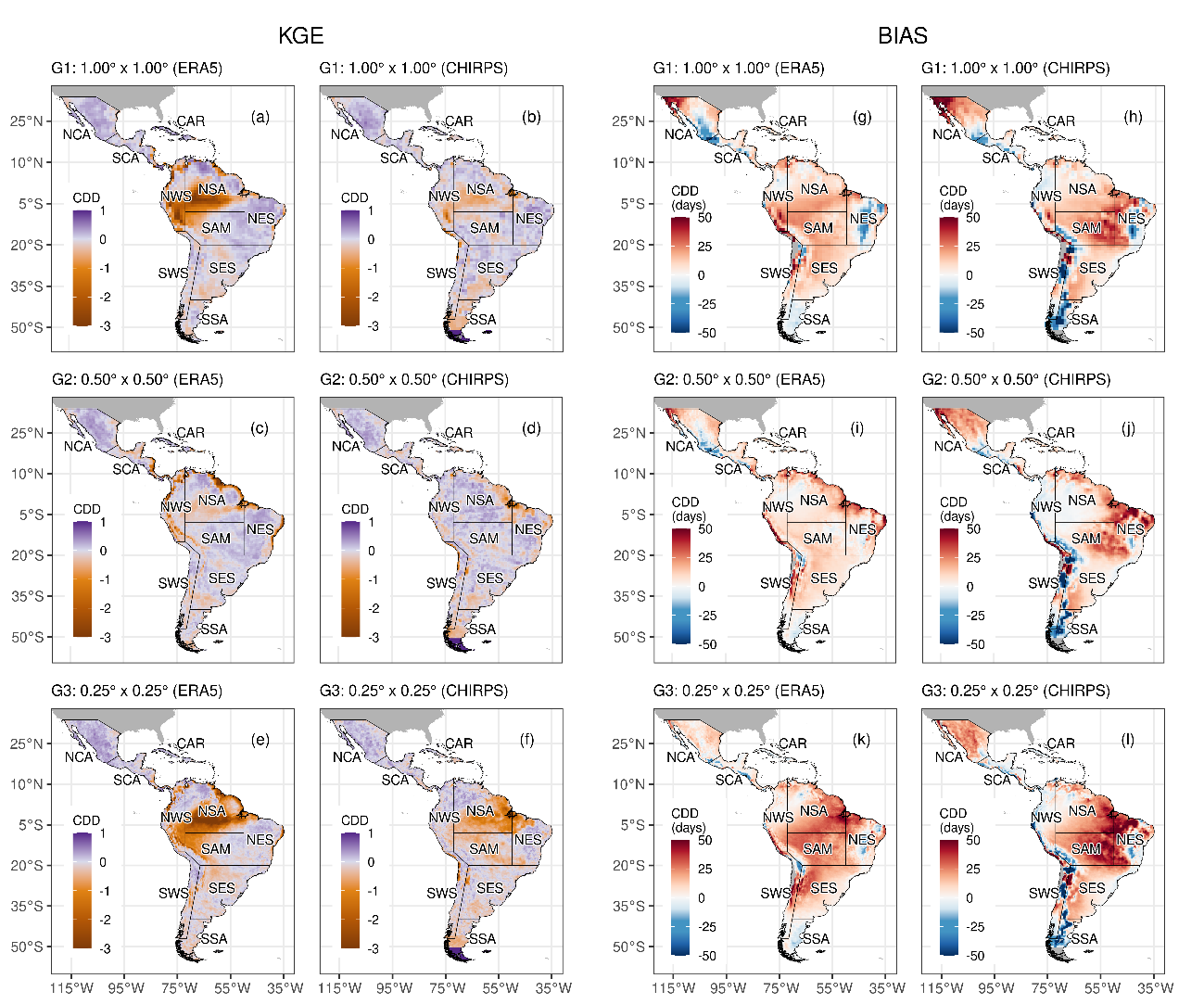


Fig. S14 The KGE (a-f) and climatology bias (g-l) of multi-model ensemble (MME) for the CDD during 1981-2014 compared between the ERA5 (left side) and GMFD (right side). The G_1_L-MME, G_2_I-MME, G_3_H-MME are the groups based on size of grid (sg) of the MME: low (≥0.8° sg ≤1.87°), intermediate (≥0.5° sg ≤0.7°), and high resolution (≥0.23° sg ≤0.35°), respectively.
